# Human brain gene expression profiles of the cathepsin V and cathepsin L cysteine proteases, with the PC1/3 and PC2 serine proteases, involved in neuropeptide production

**DOI:** 10.1016/j.heliyon.2018.e00673

**Published:** 2018-07-03

**Authors:** Sonia Podvin, Aneta Wojnicz, Vivian Hook

**Affiliations:** a Skaggs School of Pharmacy and Pharmaceutical Sciences, Division of Pharmaceutical Sciences, University of California San Diego, La Jolla, California, USA; b School of Medicine, Dept. of Neurosciences, University of California San Diego, La Jolla, California, USA; c School of Medicine, Dept. of Pharmacology, University of California San Diego, La Jolla, California, USA

**Keywords:** Neuroscience

## Abstract

Proteases are required to generate active peptide neurotransmitters, known as neuropeptides, from pro-neuropeptides. Model animal systems have recently illustrated roles for the cathepsin V (CTSV) and cathepsin L (CTSL) cysteine proteases, combined with the serine proteases PC1/3 (PCSK1) and PC2 (PCSK2), and exopeptidases in the production of neuropeptides. There is notable interest in the human-specific cathepsin V gene which is not present in rodent and other animal models used in prior studies of neuropeptide production. A gap in the field is knowledge of the human brain gene expression patterns of these neuropeptide-producing protease systems. Therefore, the goal of this study was to characterize the expression profiles of these pro-neuropeptide processing proteases in human brain. Quantitative gene expression microarray data for 169 human brain regions was obtained from the Allen Institute Human Brain Atlas resource, analyzed as log_2_ of gene expression intensity normalized to the mean of human genes (21,245 genes) expressed in human brain. These proteases had log_2_ values of 2–12, indicating expression levels above the average of all genes in the human brain, with varying expression levels among the 169 brain regions. CTSV and CTSL displayed moderate to high expression values of 1.9–8.6 and 7.1–10.6, respectively. Interestingly, CTSV and CTSL showed high expression in white matter composed of myelinated axons, consistent with the knowledge that neuropeptide production occurs in axons within transported neuropeptide secretory vesicles to nerve terminals. PCSK1 had a broad range of moderate to very high expression with log_2_ of 2–12. PCSK2 had somewhat lower expression levels than PCSK1. The exopeptidase genes RNPEP, CTSH, and CPE each showed fairly even levels of expression throughout the brain, with CPE displaying high expression. The prevalence of these processing proteases throughout human brain regions, including areas rich in neuropeptides such as hypothalamus, is consistent with their roles for neuropeptide production. Further, proenkephalin and NPY precursors, substrates of CTSV and CTSL shown in prior model animal studies, were co-expressed with CTSV and CTSL. These data demonstrate that the human brain expresses the neuropeptide-producing cysteine and serine proteases, with exopeptidases, throughout a multitude of brain regions.

## 1 Introduction

Neuropeptides are essential as neurotransmitters and neurohumoral effectors for cell-cell communication in the brain and neuroendocrine systems (Krieger et al., 1983; Palkovits, 2002; Kastin, 2006). Neuropeptides represent the largest class of neurotransmitters, and function together with the small classical neurotransmitter molecules (Siegel et al., 1999). Diverse neuropeptides are expressed in virtually all brain regions, and they participate in a wealth of brain functions. For example, the opioid neuropeptides enkephalin, β-endorphin, and dynorphin regulate analgesia and are important in chronic pain conditions (Inturrisi, 2002; Charbogne et al., 2014; Podvin et al., 2016). Galanin regulates cognition in normal and neurodegeneration conditions (Steiner et al., 2011; Counts et al., 2001). Corticotropin releasing hormone (CRH) participates in stress responses (Krohg et al., 2008; Shekhar et al., 2005). Diverse neuropeptides participate in brain functions in health and in neurological diseases.

**Fig. 1:**
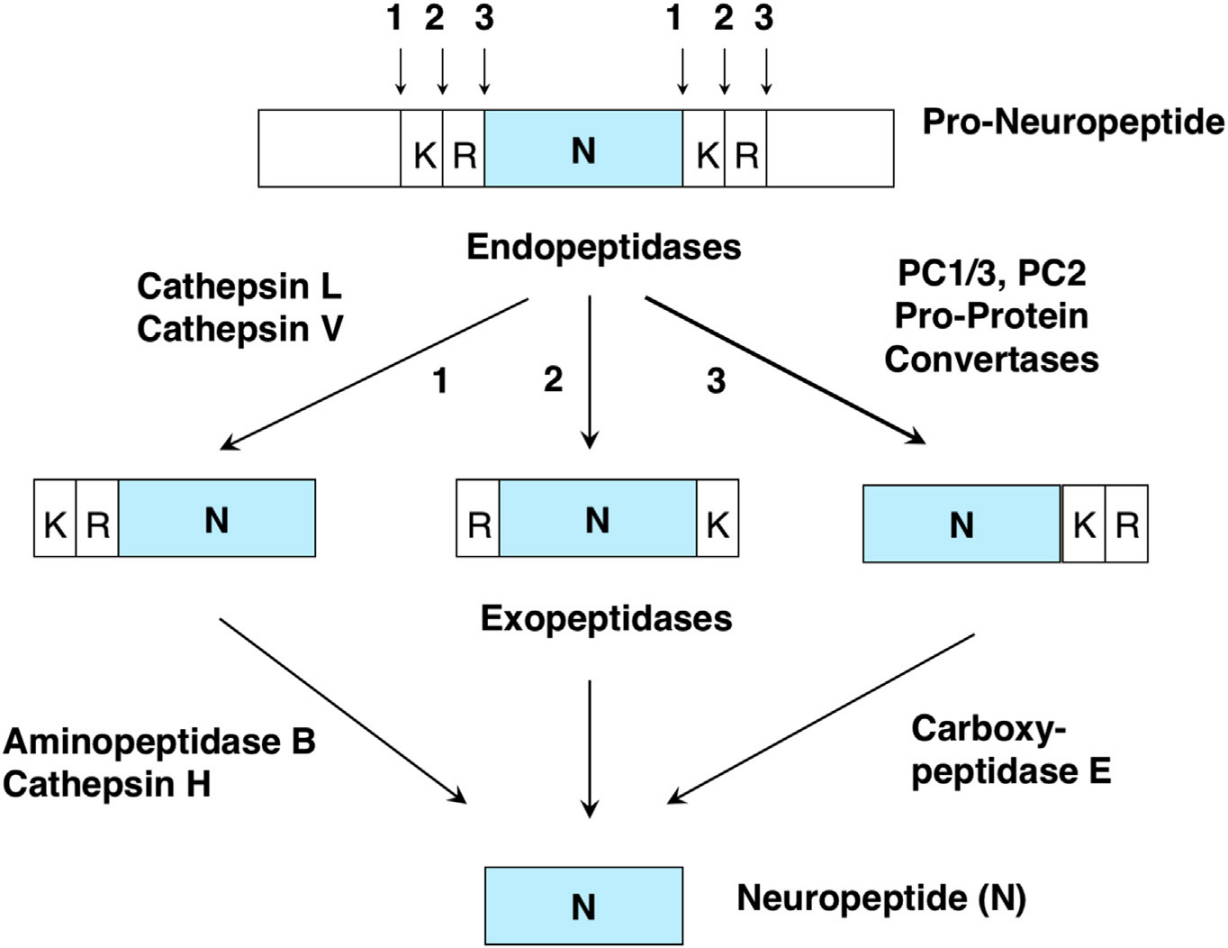
Cysteine and serine protease pathways for neuropeptide production. Cysteine and serine subtilisin-like protease pathways are involved in neuropeptide production from pro-neuropeptide precursors (9–11). The cathepsin V (CTSV) and cathepsin L (CTSL) cysteine proteases cleave pro-neuropeptides at paired basic residues. Resultant peptide intermediates require removal of N-terminal basic residues by aminopeptidase B (RNPEP) or cathepsin H (CTSH), and removal of C-terminal basic residues by carboxypeptidase E (CPE). The cathepsin V cysteine protease functions in human cells for neuropeptide production, but not in rodent or other mammalian species previously used for studies of neuropeptide-producing proteases (28–33) since these species do not have the cathepsin V gene (23–26). The serine protease pathway consists of the subtilisin-like pro-protein convertases (PC) PC1/3 and PC2 (PCSK1 and PCSK2, respectively) that cleave at paired basic residues. Resultant peptide intermediates require removal of C-terminal basic residues by CPE.

Neuropeptides are synthesized as pro-neuropeptide precursors that require proteolytic processing to generate the small active neuropeptides of ∼3–40 residues in length (Fig. 1) (Hook et al., 2008; Seidah and Prat, 2012). Recent studies have demonstrated the novel role of the human cathepsin V cysteine protease in the production of enkephalin and NPY neuropeptides in human cells (Funkelstein et al., 2012). Gene silencing of cathepsin V reduces production of enkephalin by more than 80% in human neuroblastoma cells, and expression of cathepsin V increases production of enkephalin and NPY. Cathepsin V cleaves proenkephalin at paired basic residue processing sites and is localized in secretory vesicles, the main organelle where neuropeptide biosynthesis occurs. Notably, cathepsin V is expressed by the human genome, but not by rodent, bovine, and other non-human mammalian species studied as model systems of proteases for neuropeptide biosynthesis (Zhou et al., 2015; Adachi et al., 1998; Bromme et al., 1999). These findings demonstrate participation of human cathepsin V in neuropeptide production.

The mammalian cathepsin L gene is most closely related to cathepsin V among the cysteine cathepsin family members (Zhou et al., 2015; Adachi et al., 1998; Bromme et al., 1999). Studies in model rodent and bovine species have shown that cathepsin L participates in generating the opioid family of neuropeptides (Hook et al., 2008; Hook and Bandeira, 2015), as well as NPY, CCK, and galanin neuropeptides from their respective pro-neuropeptides (Funkelstein et al., 2008a;Funkelstein et al., 2008b; Beinfeld et al., 2009; Lu et al., 2012). These studies have validated of the role of cathepsin L in neuropeptide production as demonstrated by cathepsin L gene knockout in mice, which results in significant reductions of (Met)-enkephalin, dynorphin A, and β-endorphin opioid neuropeptides, as well as NPY, CCK, and galanin in brain. Cathepsin L is present in secretory vesicles, where it cleaves pro-neuropeptides at dibasic residues. In light of the presence of the human-specific CTSV and CTSL homologue genes in the human genome, it is of interest to compare gene expression patterns of CTSV to CTSL in the human brain. Potential differences in human brain expression patterns between cathepsin V and L could potentially be related to human specific mechanisms of pro-neuropeptide processing.

Following cathepsin V and cathepsin L processing of pro-neuropeptides, the resultant peptide intermediates are further processed to remove N- and C-terminal basic residues by aminopeptidase B (RNPEP) (Hwang et al., 2007; Hwang and Hook, 2008) or cathepsin H (CTSH) (Lu et al., 2012), and carboxypeptidase E (CPE) (Hook et al., 2008; Fricker, 1988), respectively, to generate mature neuropeptides. These findings support the roles of the cathepsin V and cathepsin L cysteine protease pathway composed of the endopeptidases with the exopeptidases RNPEP, CTSH, and CPE, for neuropeptide production.

In addition, approaches of gene knockout, expression, and biochemical characterization have provided much evidence for the established roles of the subtilisin-like pro-protein convertase (PC) proteases PC1/3 and PC2 in neuropeptide production (Hook et al., 2008; Seidah and Prat, 2012). The PC1/3 and PC2 serine protease pathway includes CPE for production of mature neuropeptides. The roles of PC1/3, PC2, and CPE for neuropeptide production has been demonstrated by peptidomics studies in gene knockout mice that lack each of these proteases (Wardman et al., 2010; Zhang et al., 2008, 2010).

To gain understanding of how the human brain expresses the cysteine (CTSV and CTSL) and serine (PCSK1 and PCSK2) protease pathway components, with exopeptidases (CPE, CTSH, and CPE), this study characterized the gene expression profiles of these proteases in 169 brain regions. Quantitative microarray gene expression data for this study was obtained from the Allen Human Brain Atlas resource, an open access database of all genes expressed in human brain (Hawrylycz et al., 2012; Sunkin et al., 2013). Protease gene expression was also compared with that of pro-neuropeptide substrates of cathepsin V, proenkephalin and proneuropeptide Y, demonstrated in neuronal cultures (Funkelstein et al., 2012), as examples of pro-neuropeptides that could be processed by these proteases in human brain. Results demonstrated similarities and differences in the gene expression profiles of these neuropeptide-producing proteases represented by the cysteine proteases cathepsin V and cathepsin L, the serine proteases PC1/3 and PC2, along with the exopeptidases aminopeptidase B, cathepsin H, and carboxypeptidase E. Results demonstrate that the human brain displays patterns of neuropeptide-producing cysteine and serine protease pathway expression throughout the brain, which supports their hypothesized role in human neuropeptide production.

## 2 Materials and methods

### 2.1 Allen Human Brain Atlas resource for gene expression data for adult neurotypical brain tissues

**Fig. 2:**
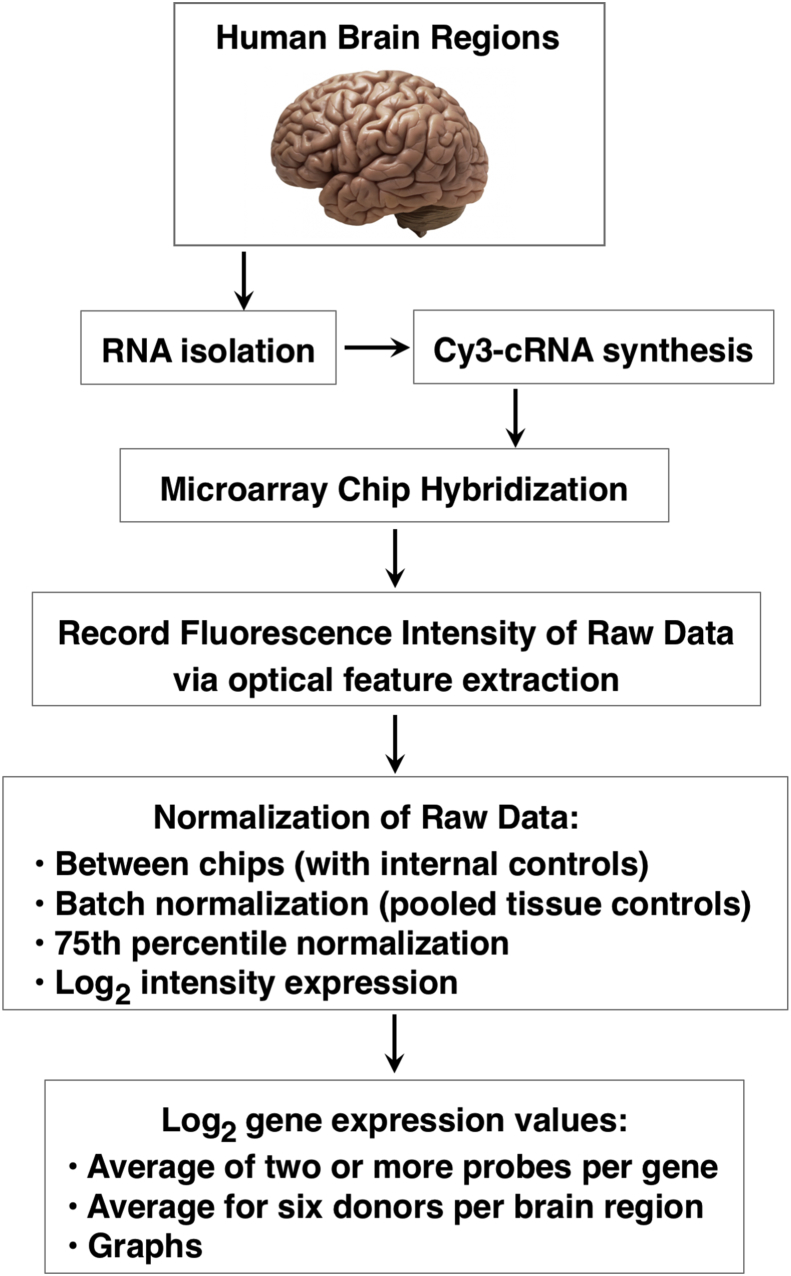
Human brain gene expression analyses by microarrays and data normalization. Gene expression data for 169 human brain regions was achieved by microarrays conducted by the Allen Human Brain Atlas resource as described in the methods. Neurotypical brains (from six donors) were dissected, RNA was isolated, and cRNA-Cy3 was synthesized for microaray chip hybridzation. The chip hybridization fluorescence raw data was obtained by optical feature extraction. This data was normalized between chips by internal controls, subjected to batch normalization with pooled tissue controls. Data were normalized as log_2_ expression intensity values where the 75th percentile of expression was approximately log = 5, and the 95th percentile of expression was approximately log_2_ = 7, calculated as described by Rizzo et al., (2014) (40). Log_2_ expression values presented in graphs of this study are log_2_ expression values that are averages ± MAD (median absolute deviation) for six donor brains (n = 6).

This study utilized the Allen Human Brain Atlas resource which integrates anatomic and genomic information (Allen Human Brain Atlas, http://www.brain-map.org/) Hawrylycz et al., 2012; Sunkin et al., 2013 for quantitative gene expression data of human genes, including the neuropeptide-producing proteases and several pro-neuropeptides of this study. These data were achieved by the gene expression microarray workflow illustrated in Fig. 2. Microarray data provided gene expression analyses for 169 brain regions from six adult neurotypical brains. All samples were collected only after obtaining informed consent from deceased's next-of-kin. These brains were collected by tissue repositories of the NICHD Brain and Tissue Bank at the Univ. of Maryland and the UC Irvine Psychiatry Brain Donor Program with IRB approval. All tissues were from adults with absence of brain injury or disease, epilepsy, drug/alcohol dependency, more than one hour on a ventilator, positive for infectious diseases, prion disease, chronic renal failure, cancer, or time since death of greater than 30 hours. Furthermore, post-mortem microscopic and macroscopic evaluation was conducted to confirm normal neuroanatomy. The six adult donors providing brain tissues for the microarray analyses were 24, 31, 39, 49, 55, and 57 years old, as described in the technical white paper #1 “Case Qualification and Donor Profiles” by the Allen Brain Atlas (http://human.brain-map.org/, ‘Documentation’ tab) (documents are also provided in supplemental information). The six donors consisted of five males and one female. It is realized that a larger portion of females would be desirable, but the extensive criteria to find brain tissues appropriate for the extensive RNA microarray analyses of ∼21,245 human genes may have been a limiting factor in identifying quality tissues for this extensive human brain study. Accordingly, this donor group as n = 6 can be described as a pilot cohort. It will be of interest in future studies to examine the novel findings from this study in larger groups of brain samples.

### 2.2 RNA preparation for microarrays

Fresh frozen brain tissues were dissected manually or by laser-capture microdissection for RNA purification, resulting 169 human brain regions collected for analyses. For manual dissection, 50–200 mg tissue per region was excised from frozen samples, and RNA was isolated using TRIZOL reagent (Thermofisher Scientific). Only samples with RIN (RNA integrity number) of >6.0 were used for the study [39]. Laser-capture microdissection (LMD) was conducted by defining anatomical structural boundaries, based on Nissl staining, to guide LMD with a Leica LMD6000 system. Captured tissue (36 mm^2^) was subjected to RNA isolation by MELT method (multi-enzymatic liquefaction of tissue) (protocol by Ambion company). RNA integrity number (RIN) (Schroeder et al., 2006) was utilized to set the RNA integrity criteria of >6.0. Total RNA samples were converted to Cy3-labeled cRNA using the Agilent ultra-low input quick amplification kit.

### 2.3 Microarray analyses of gene expression

**Fig. 3:**
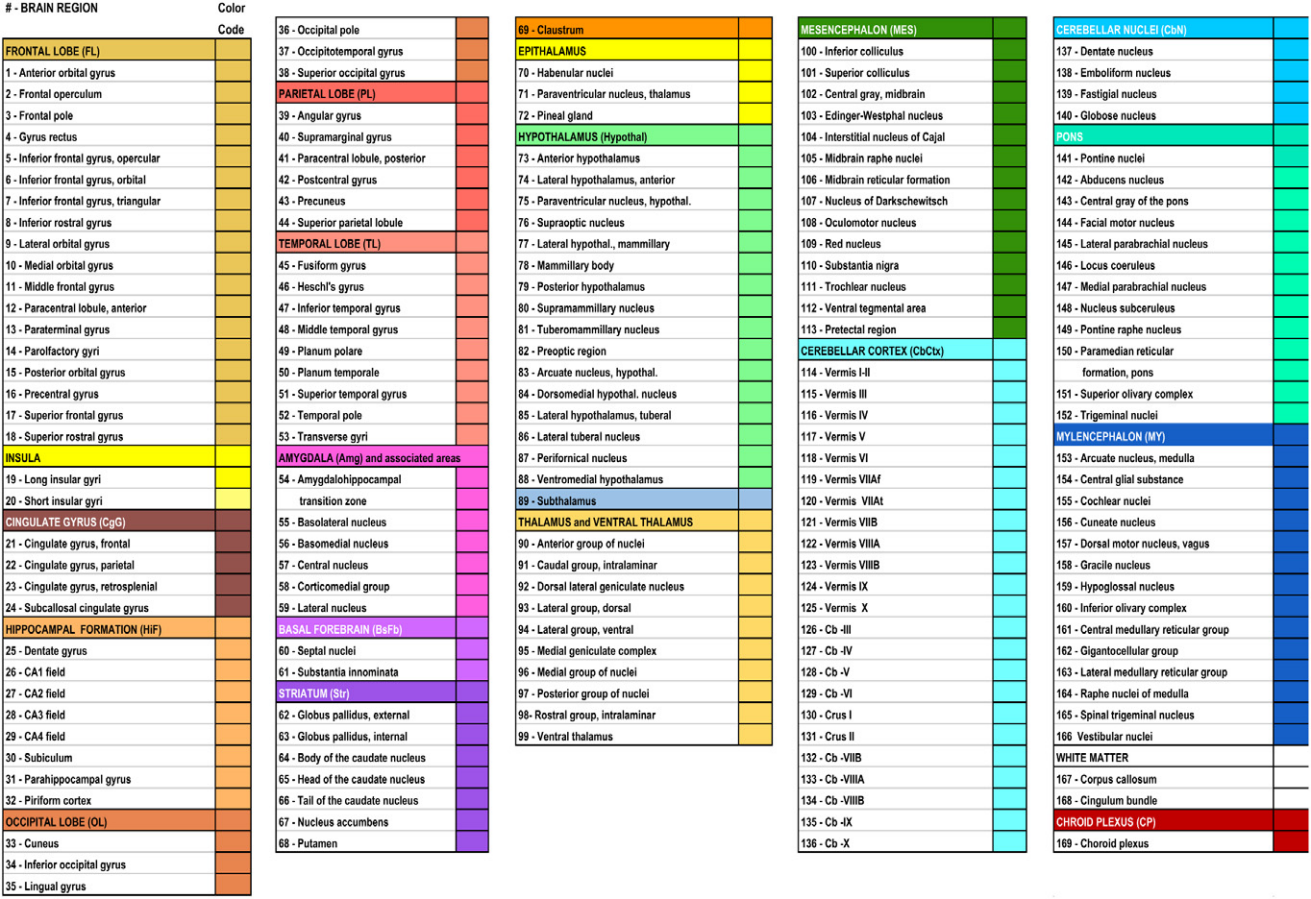
169 Human brain regions. 169 human brain regions are listed numbered #1–169, named according to main region (color coded) and subregions. These color-coded brain regions are used in the graphs to display gene expression values for the neuropeptide-producing protease genes (Figs. 4, 5, and 6) and the pro-neuropeptide genes of PENK and NPY (Fig. 7).

The microarray chip design utilized an Agilent 8 × 60K array including the Agilent Whole Human Genome probe set with multiple probes for 21,245 human genes. Cy3-labeled cRNA (600 ng) was applied to each array, hybridized, and scanned. The raw expression values correspond to the fluorescence optical intensity of the Cy3-cRNA, or control Cy3-RNAs, that hybridized to the complementary oligonucleotide probes on the chip. Probe sequences utilized for the protease and pro-neuropeptide gene microarray experiments of this study are provided in Supplemental figure S1. The microarray data processing and normalization processes are described in the technical white paper #2 “Microarray Survey” and technical white paper #3 “Microarray Data Normalization” at the Allen Brain Institute, (http://human.brain-map.org/, ‘Document’ tab) (see supplemental information). Microarray data was normalized across the entire set of microarray samples, and data analyzed were downloaded from the Allen Human Brain Atlas site (http://human.brain-map.org/) as log_2_ of expression values. The normalization steps aligned mean expression intensity distributions of all genes across 169 brain regions (Fig. 3).

### 2.4 Analyses of normalized microarray data with median absolute deviation (MAD)

**Fig. 4:**
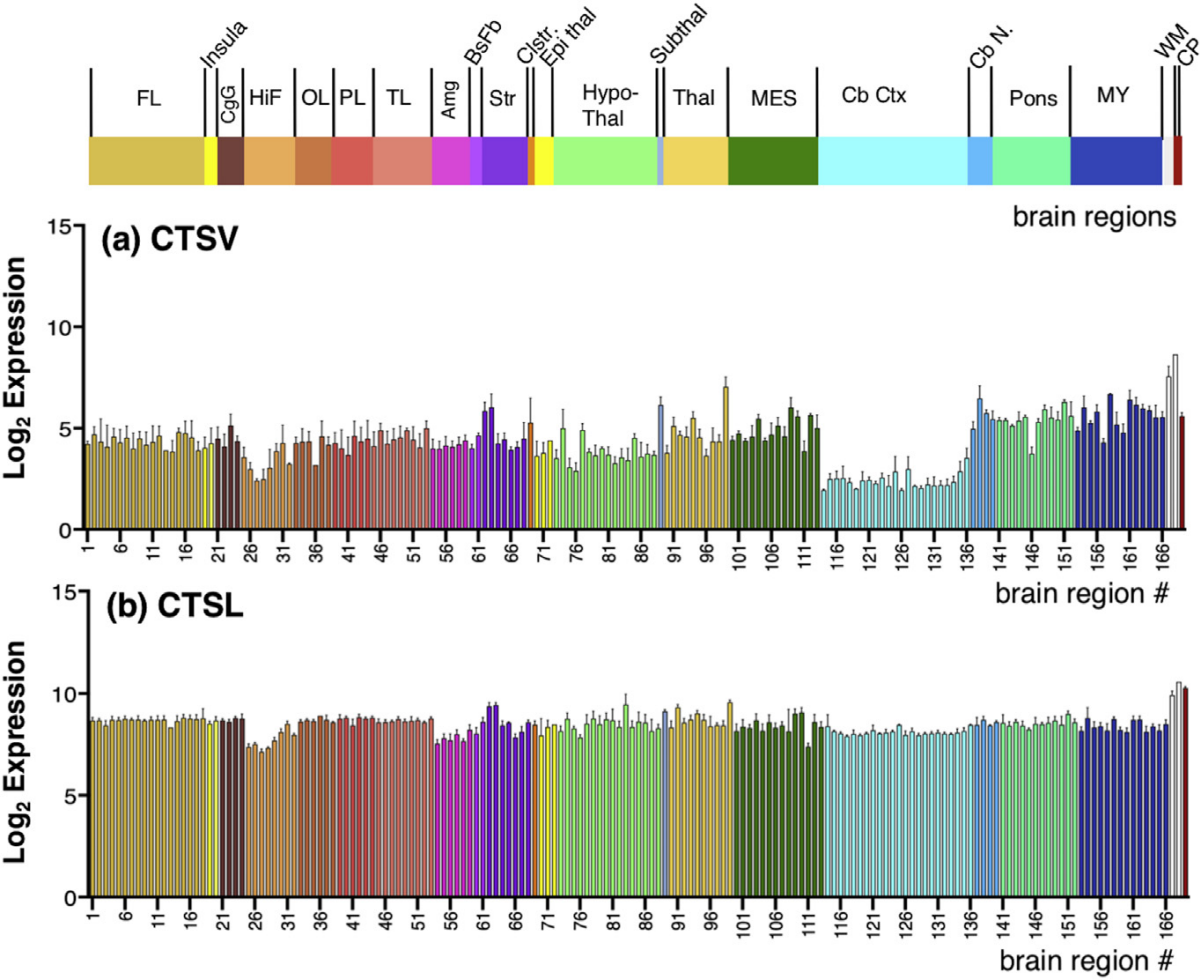
Gene expression of cathepsin V and cathepsin L cysteine proteases in human brain. Quantitative gene expression values for (a) cathepsin V (CTSV), and (b) cathepsin L (CTSL) are illustrated for 169 adult human brain regions. Microarray probes used for gene expression analyses are listed in Fig. S1. Log_2_ quantitative gene expression values were calculated for 169 human brain regions, by normalization to the average expression of all genes human genes analyzed by the Allen Human Brain Atlas resource utilized for this study, and expressed as the average log_2_ ± MAD (median absolute deviation) (n = 6 human donor brains). Brain regions are indicated by the colored bars (at top) indicating the main regions of: FL: Frontal lobe, Insula; CgG: Cingulate gyri; HiF: Hippocampal formation; OL: Occipital lobe; PL: Parietal lobe; TL: Temporal lobe; Amg: Amygdala; BsFb: Basal forebrain; Str: Striatum; Clstr: Claustrum; Epithal: Epithalamus; Hypothalamus; Thal: Thalamus; Subthal: Subthalamus; MES: Mesencephalon; CbCtx: Cerebellar cortex; CbN: Cerebellar nuclei; Pons; MY: Myelencephalon; WM: White matter structures; CP: Choroid plexus of the lateral ventricles. Color keys for these regions, with subregions numbered 1–169 are shown in Fig. 3.

**Fig. 5:**
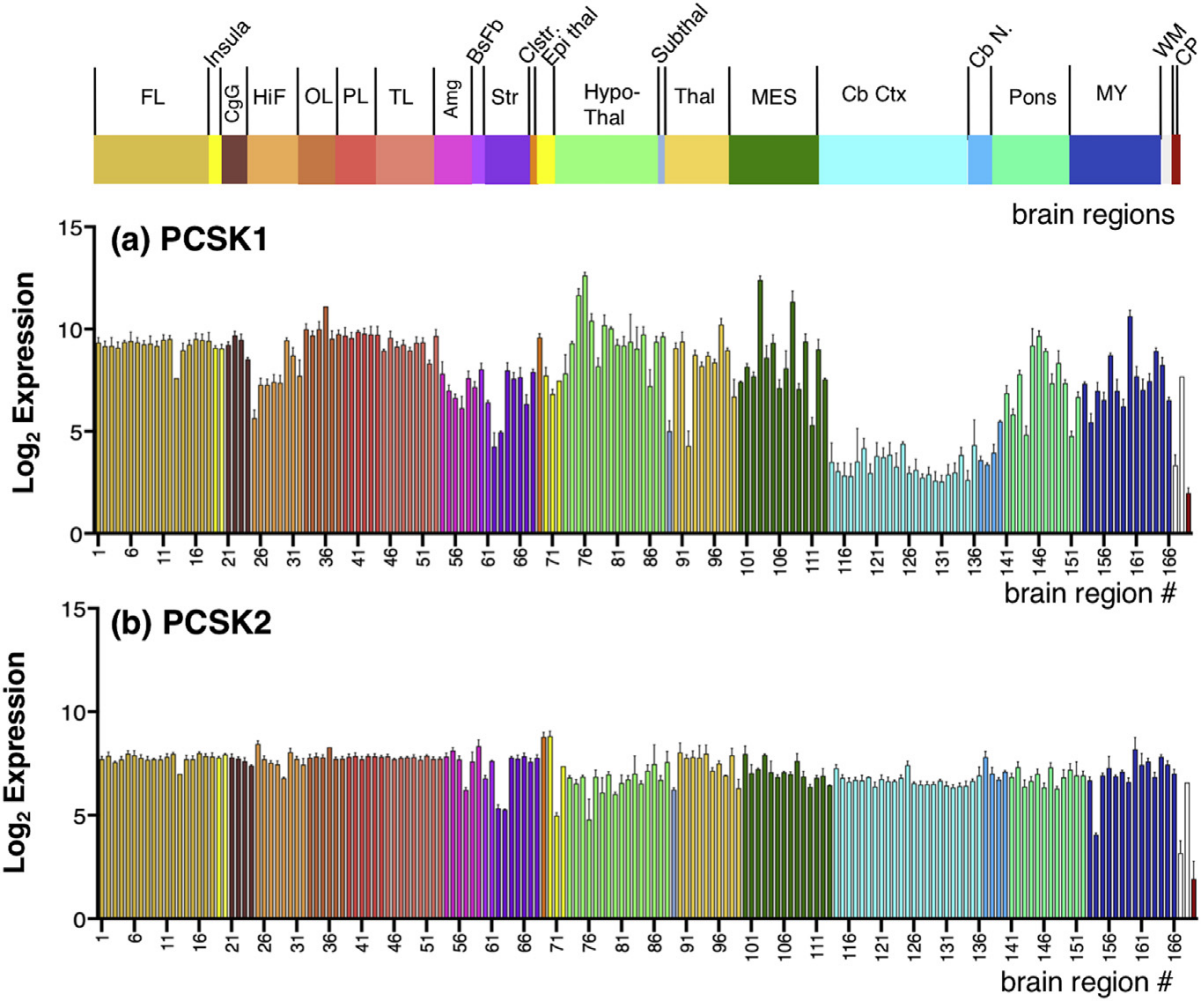
Gene expression of the pro-protein convertase PC1/3 and PC2 serine proteases in human brain. Quantitative log_2_ gene expression values for (a) pro-protein convertase PC1/3 (PCSK1) and (b) pro-protein convertase PC2 (PCSK2) are illustrated for 169 adult human brain regions. Values shown are the average of median log_2_ expression ± median absolute deviation (MAD) (from six donor brains). The 169 brain regions and subregions numbered 1–169 are shown in Fig. 3, with color keys.

**Fig. 6:**
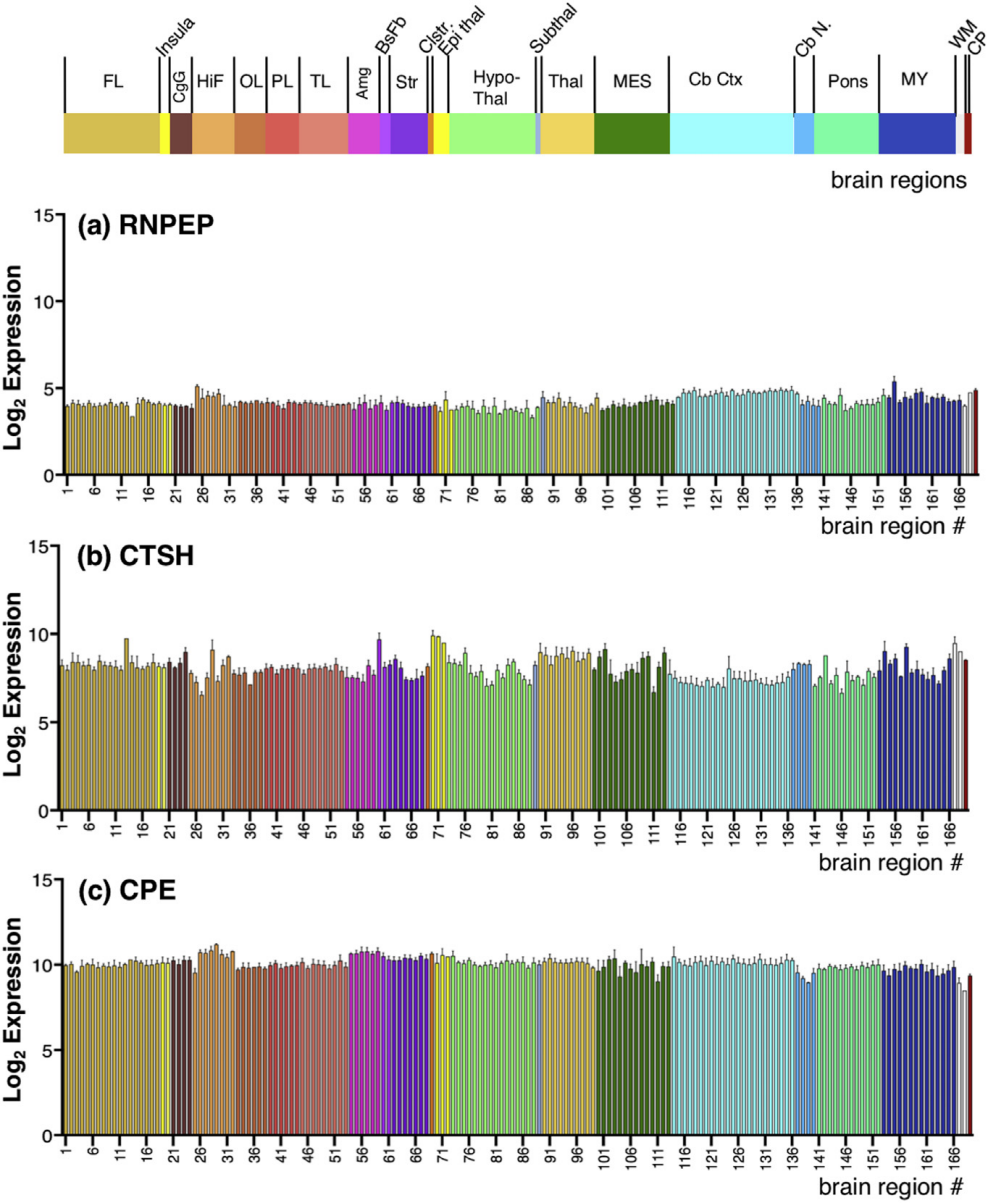
Gene expression of aminopeptidase B (RNPEP), cathepsin H (CTSH), and carboxypeptidase E (CPE) exopeptidases in human brain. Quantitative log_2_ gene expression values for (a) aminopeptidase B (RNPEP), (b) cathepsin H (CTSH), and (c) carboxypeptidase E (CPE) are illustrated for 169 human brain regions. Values shown are the average of median log_2_ expression ± median absolute deviation (MAD) (from six donor brains). The 169 brain regions and subregions numbered 1–169 are shown in Fig. 3, with color keys.

**Fig. 7:**
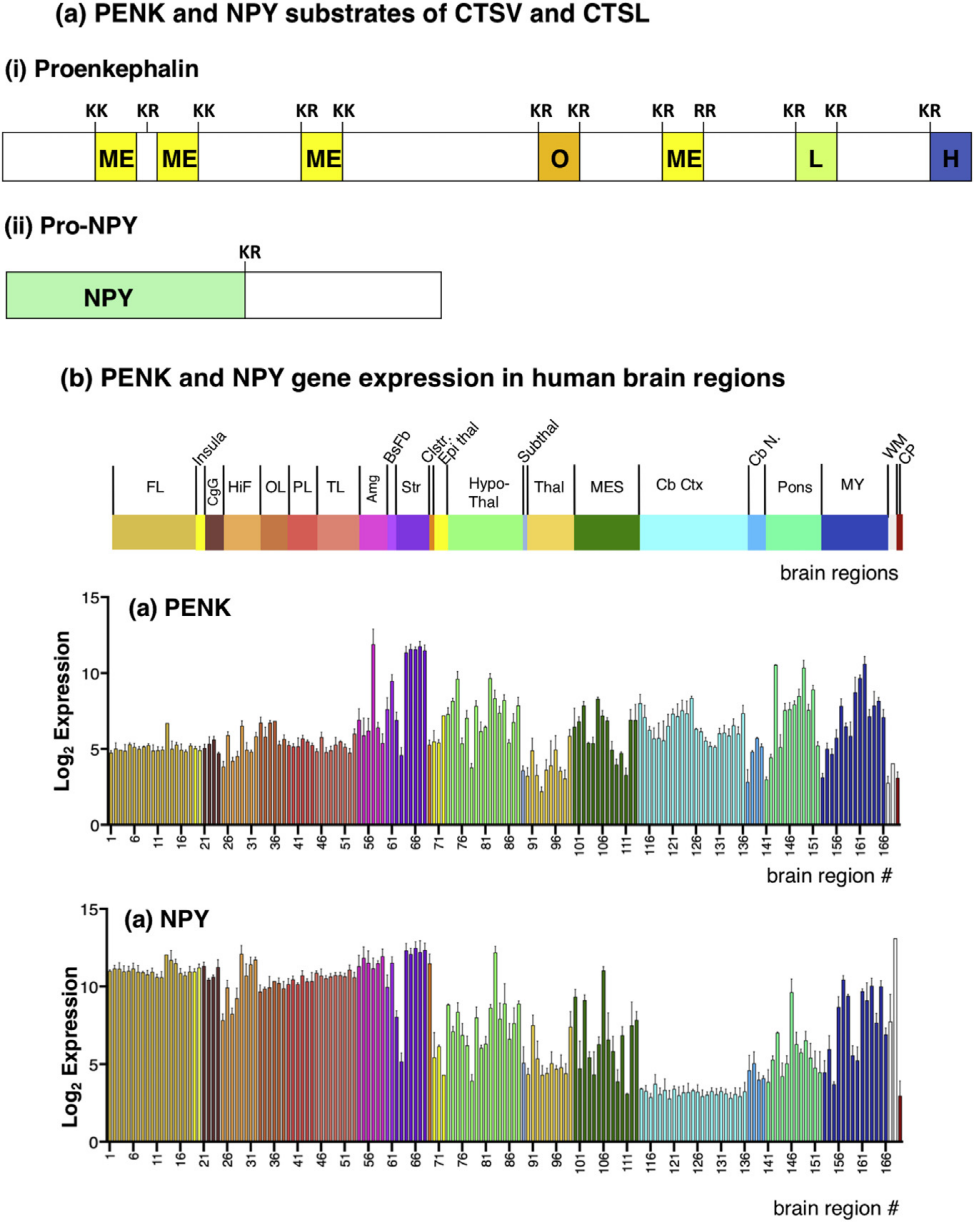
Expression of proenkephalin and pro-NPY, substrates of CTSV and CTSL. (a) Illustration of the proenkephalin and pro-NPY pro-neuropeptide substrates of cathepsin V (i) and cathepsin L (ii) are shown. (b) Quantitative log_2_ gene expression values for the proenkephalin (PENK) (i) and the NPY (NPY) (ii) genes are illustrated for 169 brain regions (brain regions listed in Fig. 3). Values shown are the average of median log_2_ expression ± median absolute deviation (MAD) (from six donor brains).

The Allen Human Brain Atlas project conducted multiple quality control steps for technical and environmental variation to normalize expression among batches (several brain region dissections from a single donor), and across all six donor brains using control RNA samples, with internal and external control standards (details provided in the technical white paper #2 “Microarray Data Normalization”, human.brain-map.org, see supplemental Information). The final normalization step was to align mean expression intensity distributions across all brains using the global brain mean. The normalization process adjusted log_2_ expression values over several steps such that the log_2_ expression intensity at the 75th percentile of expression was approximately log_2_ = 5, and the 95th percentile of expression was approximately log_2_ = 7. Official gene symbols were queried for the gene expression values presented in this study (CTSV, CTSL, PCSK1, PCSK2, RNPEP, CTSH, CPE, PENK, NPY). Complete log_2_ datasets contained values for each probe for each donor in each brain region. All of these values were used to calculate the average and variation shown in bar graphs of expression (Figs. 4, 5, 6, and 7). The mean expression values for each gene were calculated as described by Rizzo et al. (2014).

Log_2_ expression values presented in graphs of this study are averages ± MAD (median absolute deviation) (n = 6 donors), which determines the data dispersion point where half of the data is closer to the median and half of the data is further from the median.

## 3 Results

### 3.1 Expression analyses of cysteine and serine protease pathway genes involved in neuropeptide production

Gene expression profiles of pro-neuropeptide processing proteases in adult human brain regions were analyzed by quantitative gene expression data obtained from the Allen Human Brain Atlas resource (http://www.brain-map.org/). The Atlas provides quantitative microarray expression analyses of 21,245 human genes (Hawrylycz et al., 2012; Sunkin et al., 2013) for 169 human brain regions (brain regions are listed in Fig. 3), provided as log_2_ expression values in reference to normalized global mean gene expression values (as explained in methods, and illustrated in Fig. 2).

### 3.2 Expression of the human cathepsin V (CTSV) cysteine protease gene in 169 regions of human brain

The profile of CTSV log_2_ expression values ranged from ∼2–8, with the majority (80%) of the brain regions exhibiting values of 2.5–5.7 (Fig. 4a). CTSV was expressed at similar expression values of 4–5 in frontal lobe (consisting of 18 subregions, 1–18 listed in Fig. 3), the insula (subregions, 19, 20), and cingulate gyrus (subregions #21–24).

The hippocampal formation (HIF) (8 subregions 25–32) showed somewhat lower CTSV expression values in the range of 2.4–4.3. Within the HIF area, the dentate gyrus, CA1, CA4, subiculum, parahippocampal gyrus, and piriform cortex had CTSV expression values of 3.0–4.2 (regions 25, 26, 29–32), and the HIF regions of CA2 and CA3 (region 27, 28) displayed lower CTSV expression values of ∼2.4.

The occipital lobe (regions 33–38) displayed expression values of ∼3.2–4.6, and the parietal lobe (regions 39–44) showed expression of ∼3.7–4.6. The temporal lobe (regions 45–53) showed values of 4.0–5.0, the amygdala (regions 54–59) had expression values of 4.0–4.3, and the basal forebrain (regions 60, 61) had values of 4.0–4.6.

In the striatal area (regions 62–68) CTSV was expressed at values of 3.9–6.0. The globus pallidus region (external and internal, regions 62, 63) expressed high levels of CTSV at log_2_ values of ∼6. The other striatal regions of the caudate nucleus, nucleus accumbens, and putamen, (regions 64–68) had expression values between 3.9 and 4.5. The claustrum (region 69) showed CTSV log_2_ expression of 5.3.

The epithalamus (regions 70–72) expressed CTSV at values of 3.6–4.4. The hypothalamus area (regions 73–88) expressed CTSV at values of 2.9–4.9. The subthalamus (region 89) had a higher level of CTSV expression, with value of 6.1. The thalamus region (regions 90–99), expressed CTSV at a broader range of values of 3.6–7.0. The mesencephalon area (regions 100–113) showed expression values of 3.9–6.0.

The cerebellar cortex area (consisting of regions 114–136) showed somewhat lower CTSV log_2_ expression values of 1.9–3.5. Cerebellar nuclei (regions 137–140) showed higher CTSV expression values of 5.0–6.5. The pontine area (regions 141–152) showed CTSV expression with values between 5.1 and 6.5, with the exception of the locus coeruleus (region 146) of 3.7. The myelencephalon area (regions 153–166) displayed expression values of 4.3–6.7.

The white matter (corpus callosum, and cingulum bundle, regions 167 and 168) showed higher levels of CTSV expression with log_2_ values of 7.5–8.6. The choroid plexus (region 169) displayed CTSV expression with log_2_ value of 5.6.

Some of the highest expression levels of CTSV were in white matter (corpus callosum and cingulum bundle), perithalamic areas (ventral thalamus and subthalamus), globus pallidus, emboliform nucleus of the cerebellum, the reticular formation (central medullary and gigantocellular groups), and nuclei of the mesencephalon and myelencephalon (gracile nucleus, superior olivary complex, red nucleus and the central glial substance).

### 3.3 Expression of the cathepsin L (CTSL) cysteine protease gene in human brain regions

The profile of CTSL gene expression in human adult brain regions was examined (Fig. 4b). The CTSL log_2_ expression values ranged from ∼7-10, showing that CTSL expression was generally higher than CTSV among the 169 human brain regions. The majority of brain regions, 80%, displayed CTSL log_2_ expression values of 8.0–8.8, consisting of the brain areas of frontal lobe (regions #1–18), insula (regions 19, 20), cingulate gyrus (regions 21–24), occipital lobe (regions 33–38), parietal lobe (regions 39–44), temporal lobe (regions 45–53), basal forebrain (regions 60, 61), epithalamus (regions 70–72), hypothalamus (regions 73–88), thalamus (regions 89–99), mesencephalon (regions 100–113), cerebellar cortex (regions 114–136), cerebellar nuclei (regions 137–140), pons area (regions 141–152), and myelencephalon (regions 153–166). CTSL expression in the hippocampal formation (HIF) (regions 25–32) and amygdala (regions 54–59) displayed expression values of ∼7.1–8.5. CTSL in the striatum (regions 62–68) had expression values of 8.1–9.4. The highest levels of CTSL expression were observed in the white matter (regions 167 and 168) and choroid plexus (region 169), with expression values of 9.9–10.5.

### 3.4 Expression of the PC1/3 and PC2 serine protease genes in human brain

The PC1/3 gene (PCSK1) displayed a broad range of expression levels among the 169 brain regions, with expression values ranging from 2.0 to 12.6 (Fig. 5a). Several areas had similar PCSK1 expression values in the range of ∼8.5–10. The frontal lobe (regions 1–18) displayed expression values of 9.0–9.5, with the exception of the paraterminal gyrus that had a value of 7.6. The insula (regions 19, 20) and cingulate gyrus (regions 21–24) areas showed expression values of 8.5–9.7. The occipital lobe (regions 33–38) and parietal lobe (regions 39–44) had similar values of 9.5–10, with the exception of the occipital pole (region 36) having a value of 11.1. The temporal lobe (regions 45–53) showed PCSK1 expression values of 8.3–9.7.

The hippocampal formation area (regions 25–32) showed more varied levels of PCSK1 expression with log_2_ expression values of 5.6–9.4. The amygdala area (regions 54–59) and basal forebrain (regions 60, 61) showed expression values of 6.1–8.0. The striatum (regions 62–68) had varied levels of expression among its subregions, ranging from 4.2 to 7.9. The claustrum (region 69) expressed PCSK1 at a value of 9.6.

The epithalamus (regions 70–72) showed expression values of 6.8–7.7. The hypothalamus (regions 73–88) displayed abundant PCSK1 with expression values of 7.8–12.6. The thalamus area (regions 90–99) had low to high expression values of 4.3–10.2. The mesencephalon (regions 100–113) showed a wide range of expression levels of 5.3–12.4. The pons (regions 141–152) and mylencephalon (regions 153–166), had varied ranges of expression with values of 4.8–10.6. The white matter (regions 167, 169) showed expression of 3.3–7.2.

Compared to other brain regions, the cerebellar cortex (regions 114–136) and the cerebellar nuclei (regions 137–140) had the lowest levels of PCSK1 expression with values of 2.5–5.4. The choroid plexus (region 169) had low expression a value of 2.0.

The PCSK2 gene showed more consistent levels of expression throughout the brain regions (Fig. 5b) compared to the wider ranges of PCSK1 expression (Fig. 5a). Approximately 80% of the brain regions showed PCSK2 expression values of 6.4–7.9. In addition, several areas had lower PCSK2 expression values of 4.0–5.3 that included the globus pallidus (region 62, 63), paraventricular nucleus of the thalamus (region 71), supraoptic nucleus (region 76), and central glial substance of the mylencephalon (region 154). Lowest PCSK2 expression was observed in the corpus callosum (region 167) and choroid plexus (region 169) with expression values of 3.2 and 1.9, respectively.

### 3.5 Expression of the exopeptidases aminopeptidase B (RNPEP), cathepsin H (CTSH), and carboxypeptidase (CPE) in human brain

Aminopeptidase B (RNPEP gene) displayed a fairly uniform level of expression throughout the brain regions, with expression values of 3.3–5.4 (Fig. 6a). CTSH gene expression among the brain regions was observed as expression values of 6.5–9.9, with a majority (80%) with values of 7.2–8.9 (Fig. 6b). CPE expression was quite constant among the 169 brain regions, with values ranging from 8.5 to 11.2 (Fig. 6c).

### 3.6 Gene expression profiles of the CTSV and CTSL substrates, proenkephalin (PENK) and pro-NPY

Processing of proenkephalin and pro-NPY precursors (illustrated in Fig. 7a) by cathepsin V has been demonstrated in human neuroblastoma cells *in vitro* (13). Processing by cathepsin L of the PENK and NPY precursors has been demonstrated in cathepsin L knockout mice (Funkelstein et al., 2012). Therefore, the expression patterns of PENK and NPY genes in human brain regions was analyzed with CTSV and CTSL, as well as with the other protease gene components involved in neuropeptide production.

PENK expression values of 5–10 were observed in the striatum, hypothalamus, cerebral cortex, pons, and mylencephalon brain regions (Fig. 7b). Higher PENK expression in the caudate nucleus, nucleus accumbens, and putamen of the striatal area (region 64–68) was observed as expression values of ∼11.5. The central nucleus of the amygdala (region 57) also showed a high expression value of 11.9. These PENK expressing regions showed CTSV and CTSL gene expression (Fig. 4), as well as expression of the PCSK1 and PCSK2 genes (Fig. 5), and the exopeptidases RNPEP, CTSH, and CPE (Fig. 6).

The NPY gene displayed expression at values of 5–10 and greater in the frontal lobe, insula, cingulate gyrus, hippocampal formation, occipital lobe, parietal lobe, temporal lobe, amygdala, and the striatum (with the exception of the globus pallidus that had lower expression of 5–8) (Fig. 7b). More moderate NPY expression with values of ∼5–8 was observed in epithalamus, hypothalamus, thalamus, mesencephalon, cerebellar nuclei, pons, and mylencephalon. Lower NPY expression was observed in the cerebellar cortex with values of 2.9–3.4. These regions displayed expression of CTSV as well as CTSL, along with PCSK1, PCSK2, and the exopeptidases RNPEP, CTSH, and CPE genes.

## 4 Discussion

This study evaluated the human brain gene expression profiles of the neuropeptide-producing proteases consisting of the CTSV and CTSL cysteine proteases, the PCSK1 and PCSK2 serine proteases, and the exopeptidases RNPEP, CTSH, and CPE. Quantitative microarray gene expression data for 169 human brain regions was obtained from the Allen Human Brain Atlas resource, an open access database of all genes expressed in human brain (Hawrylycz et al., 2012; Sunkin et al., 2013). Gene expression values were normalized to the global reference mean among all human genes analyzed (21,245 genes), and log_2_ ratios of each gene and the reference mean were utilized for analyses. These data represent initial findings based on the small cohort of subjects (n = 6) selected by the Allen Hunan Brain Atlas program, likely due to their strict quality criteria for mRNA analyses among the numerous brain regions. This dataset provides new insight into the expression patterns of pro-neuropeptide processing proteases throughout the human brain that are necessary for production of essential peptide neurotransmitters.

Overall, the data show that the human brain globally expresses the neuropeptide biosynthetic protease machinery, demonstrated by all protease gene expression values of ∼ log_2_ ≥ 2 in the 169 human brain regions studied (Table 1). The human-specific CTSV gene was expressed at lower levels than mammalian CTSL, but globally in the human brain. The known CTSV and CTSL pro-neuropeptide substrates PENK and NPY were co-expressed with CTSV and CTSL, consistent with participation of CTSV in processing these precursors in human neuroblastoma cells (Funkelstein et al., 2012), and participation of CTSL for production of these neuropeptides in mouse brain shown by cathepsin L gene knockout (Yasothornsrikul et al., 2003; Funkelstein et al., 2008a,b; Minokadeh et al., 2010; Beinfeld et al., 2009). Further the cysteine and serine processing proteases were expressed throughout the brain, suggesting their involvement in producing numerous neuropeptides as demonstrated in animal models. The highly peptidergic hypothalamus region expressed moderate (log_2_ ∼4) to very high (log_2_ > 13) levels of these protease genes, consistent with roles of these proteases in neuropeptide production. These data suggest that because these protease genes are expressed globally among human brain regions, it is possible for these processing proteases to participate in neuropeptide production in human brain.

**Table 1:** Human brain expression of protease genes involved in neuropeptide biosynthesis.

Gene	Gene Expression Values (Log_2_)		
	Range	80% Range	Median
CTSV	1.9–8.6	2.5–5.7	4.3
CTSL	7.1–10.6	8.0–8.8	8.5
PCSK1	2.0–12.6	3.5–9.7	8.1
PCSK2	1.9–8.8	6.4–7.9	7.3
RNPEP	3.3–5.4	3.8–4.7	4.1
CTSH	6.5–9.9	7.2–8.9	8.0
CPE	8.5–11.2	9.6–10.5	10.0

The range for log_2_ expression values for the 169 human brain regions is shown for each protease gene. 80% of the brain regions displayed a range of expression for each gene (brain regions are indicated in Fig. 3). The median expression value among all 169 brain regions for each gene is indicated.

Expression of the processing protease genes is the first step in the cascade of gene expression, mRNA translation, and post-translational processing required to generate active proteolytic enzymes. Levels of gene expression do not always correspond to the level of protein translated and made into active protein by the cell. Therefore, the gene expression levels evaluated here may or may not indicate relative levels of active protein generating neuropeptides in secretory vesicles. Proteolytic processing of pro-enzymes is required to generate active proteolytic enzymes for neuropeptide production. Nonetheless, the gene expression data here provide evidence that expression of the cysteine and serine protease pathways for production of neuropeptides occurs in all 169 human brain regions since all log_2_ of gene expression values were >2, indicating that it is possible for these proteases to process pro-neuropeptides globally in the brain where peptidergic neurons are present.

The very high expression of CTSV and CTSL in white matter is intriguing. White matter consists of myelinated axons that mediate communication between neuronal cell bodies and distant nerve terminals for connections and communication among brain regions and is surprisingly consistent with pro-neuropeptide processing in axonal secretory vesicles. Significantly, the expression of CTSV and CTSL in white matter is consistent with neuropeptide production in secretory vesicles as they undergo axonal transport from neuronal cell body to nerve terminals. Neuropeptide production during axonal transport has been demonstrated for pro-vasopressin and pro-oxytocin in the hypothalamo-neurohypophysial neural circuit in rat (Gainer et al., 1977; Brownstein et al., 1980). Further, mRNA expression occurs in axons to provide local protein synthesis for axonal functions (Jung et al., 2012; Preitner and Flanagan, 2012); thus, expression of CTSV and CTSL genes can occur in axons where pro-neuropeptide processing occurs. The finding of CTSV and CTSL expression in white matter is fascinating as these data are the first in human brain to illustrate these recently discovered pro-neuropeptide processing proteases in axonal structures of white matter in human brain. It will, therefore, be important in future studies to evaluate localization of cathepsin V and cathepsin L mRNAs in axons of white matter, as well as to assess the presence of these cathepsin proteins in secretory vesicles of axons in white matter.

It must be realized that CTSV and CTSL in secretory vesicles is a recently identified subcellular compartment for their functions in neuropeptide production. These cysteine proteases are also present in the lysosome organelle that is required for cellular protein homeostasis through protein degradation. Thus, the expression of CTSV and CTSL in human brain also represent their lysosomal functions (Barrett et al., 2004; Stoka et al., 2016), including white matter since lysosomes are present in axons and undergo axonal transport (Gatzinsky and Berthold, 1990). Furthermore, lysosomes are necessary for all cellular functions include the oligodendroglial cells that form the myelin sheath of axons in white matter.

The dual functions of the CTSV and CTSL cysteine cathepsin proteases in secretory vesicles and lysosomes also occur for other proteases, such as the CPE exopeptidase involved in C-terminal trimming of peptides to generate neuropeptides. It is of interest that CPE displayed the highest levels of gene expression throughout the brain compared to the other proteases examined in this study. In addition to the function of CPE in neuropeptide production, it also has non-protease functions in trafficking and sorting of proteins to secretory vesicles (Cawley et al., 2016; Cool and Loh, 1998) and has neurotrophic functions (Cheng et al., 2013). The multiple functions of CPE may represent its high expression throughout the human brain.

## 5 Conclusions

In summary, this study demonstrates the human brain gene expression patterns of the cathepsin V and cathepsin L cysteine protease genes, the PC1/3 and PC2 serine protease genes, and the exopeptidases CPE, CTSH, and CPE throughout human brain regions that are involved in neuropeptide production. These data support a role for the human cathepsin V and cathepsin L in human brain for neuropeptide production. High levels of expression of CTSV and CTSL in white matter composed of myelinated axons is an exciting finding in human brain, since pro-neuropeptide processing has been shown to occurs in axons during transport of neuropeptide secretory vesicles to nerve terminals in rat. These results illustrate the global expression profiles of neuropeptide-producing proteases in human brain, including their presence in axonal structures of white matter. These findings support the hypothesis that these proteases may participate in human brain neuropeptide biosynthesis.

## Declarations

### Author contribution statement

Sonia Podvin: Performed the experiments; Analyzed and interpreted the data; Contributed reagents, materials, analysis tools or data; Wrote the paper.

Aneta Wojnicz: Performed the experiments; Analyzed and interpreted the data.

Vivian Hook: Conceived and designed the experiments; Analyzed and interpreted the data; Contributed reagents, materials, analysis tools or data; Wrote the paper.

### Funding statement

This work was supported by a grant from the National Institutes of Health (NIH) (R01NS094597) to V. Hook.

### Competing interest statement

The authors declare no conflict of interest.

### Additional information

No additional information is available for this paper.

## Appendix A Supplementary data

Fig.S1 Probe sequences for protease and pro-neuropeptide gene expression microarray analyses. Probes for microarray analyses of the human protease and pro-neuropeptide genes investigated in this study are listed. The majority of the protease and pro-neuropeptide genes were analyzed by two different probes. Fig.S1

Technical White Paper _spl_1 Technical white paper #1 “Case Qualification and Donor Profiles” October 20132 v.7. Available for download at https://help.brain-map.org/display/humanbrain/Documentation. Technical White Paper _spl_1

Technical White Paper _spl_2 Technical white paper #2 “Microarray Survey” October 2013 v.7. Available for download at https://help.brain-map.org/display/humanbrain/Documentation. Technical White Paper _spl_2

Technical White Paper _spl_3 Technical white paper #3 “Microarray Data Normalization” March 2013 v.1. Available for download at https://help.brain-map.org/display/humanbrain/Documentation. Technical White Paper _spl_3
