# Supplementary material for: Human brain gene expression profiles of the cathepsin V and cathepsin L cysteine proteases, with the PC1/3 and PC2 serine proteases, involved in neuropeptide production
Source: Heliyon. 2018 Jul 3;4(7):e00673. doi: 10.1016/j.heliyon.2018.e00673 (PMC6037879; doi:10.1016/j.heliyon.2018.e00673)
Supplement: Fig.S1 — Probe sequences for protease and pro-neuropeptide gene expression microarray analyses. Probes for microarray analyses of the human protease and pro-neuropeptide genes investigated in this study are listed. The majority of the protease and pro-neuropeptide genes were analyzed by two different probes. [file mmc1.pdf]

<u>Fig. S1 Microarray Probe Sequences for Proteases and Pro-Neuropeptide Gene Expression Studies</u>

Probes for microarray analyses of the human protease and pro-neuropeptide genes investigated in this study are listed. The majority of the protease and pro-neuropeptide genes were analyzed by two different probes.

<u>Proteases</u>

CTSV Gene ID 1504 Chromosome 9

PROBE ID	Probe name	Sequences (5'-3'):
(1) 1057831	A_23_P146456	GAAGGACTTAAGGACAGCATGTCTGGGGAAATTTTATCTTGAAACTGACCAAACGCTTAT
(2) 1057832	CUST_7879_PI416261804	GGTTGGCTACGGCTTTGAAGGAGCAAATTCGAATAACAGCAAGTATTGGCTCGTCAAAAA

CTSL Gene ID 1503 Chromosome 9

PROBE ID	Probe name	Sequences (5'-3'):
(1) 1030114	A_23_P94533 9	AAGACATGGATCATGGTGTGCTGGTGGTTGGCTACGGATTTGAAAGCACAGAATCAGATA
(2) 1030115	CUST_15547_PI416261804	ACTGTGGGGCCCATTTCTGTTGCTATTGATGCAGGTCATGAGTCCTTCCTGTTCTATAAA

PCSK1 Gene ID 5090 Chromosome 5

PROBE ID	Probe name	Sequences (5'-3'):
(1) 1053306	A_23_P213508	CACAGAGACGACCGGCTGCTTCAAGCTCTGGTGGACATTCTGAATGAGGAAAATTAAAAT
(2) 1053305	A_24_P174793	GAGTTTAACATGTGTGGTCTTGGTATTCTTAAGGGAACTTCCACATTATACATTTGATGT

PCSK2 Gene ID 5094 Chromosome 20

PROBE ID	Probe name	Sequences (5'-3'):
(1) 1053300	A_23_P79968 20	TGTCTCGCTAGTGATGTTTTTATGATATCCCTGATCCTAACTGAAGAGACAGTTATTTAT
(2) 1053301	CUST_13562_PI416261804	AGAAGTTTCTGCTGCCGCCAACAACAATATCTGTGGAGTTGGAGTAGCATACAACTCCAA

RNPEP Gene ID 6019 Chromosome 1

PROBE ID	Probe name	Sequences (5'-3'):
(1) 1052146	A_23_P97770 1	ACCAGGAAGATTTCTGGAAAGTGAAGGAGTTCCTGCATAACCAGGGGAAGCAGAAGTATA
(2) 1052147	A_24_P68649	GCAACAGGAGAGAAGCTTTTTGGACCTTATGTTTGGGGAAGGTATGACTTGCTCTTCATG

CTSH Gene ID 1501 Chromosome 15

PROBE ID	Probe name	Sequences (5'-3'):
(1) 1023745	A_23_P14774	ACCAGCCATGTGCCTTAGTGTCCTTCTTAACAGACTCAAACCACATGGACCACGAATATT
(2) 1023746	CUST_10722_PI416261804	AAGTTCCAACCTGGAAAGGCCATCGGCTTTGTCAAGGATGTAGCCAACATCACAATCTAT

CPE Gene ID 1354 Chromosome 4

PROBE ID	Probe name	Sequences (5'-3'):
(1) 1057996	A_23_P259442	TCTTGTGCTGACTAACTATAAGCATGATCTTGTTAATGCATTTTTGATGGGAAGAAAAGG
(2) 1057997	A_32_P34372	ACTCCGAGCTGTATCTGTTAACCTTTTCAATAGCATTCATTATGTGACATTGTCACCAAT

Pro-Neuropeptides

PENK Gene ID 5179 Chromosome 8

PROBE ID	Probe name	Sequences (5'-3'):
(1) 1018602	A_23_P417918	TTTGCTAGCCAAAAGGTATGGGGGCTTCATGAAAAGGTATGGAGGCTTCATGAAGAAAAT
1053200	CUST_11041_PI416261804	TCTCTCCTCGCAGTCCATGGCGCGGTTCCTGACACTTTGCACTTGGCTGCTGTTGCTCGG
1013153	CUST_11043_PI416261804	TTCCAATTGGCCTGCTCCATCCGAACAGCGTCAACTCCATGGCGCGGTTCCTGACACTTT
(2) 1010876	CUST_15_PI416558187	TTCATTGTCTGGATAACTATACAACCTGAAAACTGTCATTTCAGGTTCTGTGCTCTTTTT

NPY Gene ID 4821 Chromosome 7

PROBE ID	Probe name	Sequences (5'-3'):
(1) 1053652	A_23_P256470	TTGAAGACCCTGCAATGTGGTGATGGGAAATGAGACTTGCTCTCTGGCCTTTTCCTATTT
(2) 1053653	CUST_16166_PI416261804	AGAGACACTGATTTCAGACCTCTTGATGAGAGAAAGCACAGAAAATGTTCCCAGAACTCG
