# Supplementary material for: Human brain gene expression profiles of the cathepsin V and cathepsin L cysteine proteases, with the PC1/3 and PC2 serine proteases, involved in neuropeptide production
Source: Heliyon. 2018 Jul 3;4(7):e00673. doi: 10.1016/j.heliyon.2018.e00673 (PMC6037879; doi:10.1016/j.heliyon.2018.e00673)
Supplement: Technical White Paper _spl_1 — Technical white paper #1 “Case Qualification and Donor Profiles” October 20132 v.7. Available for download at https://help.brain-map.org/display/humanbrain/Documentation. [file mmc2.pdf]

ALLEN INSTITUTE *for* BRAIN SCIENCE
Fueling Discovery

ALLEN **Human Brain Atlas**

TECHNICAL WHITE PAPER: CASE QUALIFICATION AND DONOR PROFILES

The case review process described here was employed for three components of the ALLEN **Human Brain Atlas**: (1) the Microarray Survey; (2) the Neurotransmitter Study; and (3) the Subcortex Study. Data for all other components of the Allen Human Brain Atlas were generated using banked tissue that underwent a separate screening process (see *In Situ Hybridization in the Allen Human Brain Atlas* white paper).

In general, postmortem tissue from males and females between 18 – 68 years of age and no known history of neuropsychiatric or neurological conditions ('control' cases) were eligible for inclusion in the Microarray Survey, Neurotransmitter Study, and Subcortex Study components of the Allen Human Brain Atlas. Key conditions for exclusion were:

- Brain injury or disease
- Epilepsy
- Drug/alcohol dependency
- > 1 hour on ventilator
- Positive for infectious disease
- Prion disease
- Chronic renal failure
- Cancer deaths
- Brain cancer
- Time since death > 30 hours

Brain tissue, cerebrospinal fluid and blood samples were collected after obtaining informed consent from decedent's next-of-kin. Institutional Review Board (IRB) review and approval was obtained for collection of tissue and non-identifying case information at the tissue banks and repositories that provided tissue for this project. Following tissue collection and freezing, additional tests and quality measures were performed to ensure the tissue and RNA met quality control (QC) criteria, and to rule out any previously undetected conditions incompatible with a 'control' diagnosis.

A Case Review Committee (CRC) of internal and external advisors reviewed all data and approved cases for inclusion in each study. The schematic in Figure 1 shows a timeline of formal CRC activities in relation to availability of screening data. A summary of screening tests and quality control measures and criteria is provided in Table 1. Specific donor profiles are provided in subsequent tables.

For additional detailed methodological information regarding these studies, please access the following technical white papers:

- *Microarray Survey in the Allen Human Brain Atlas*
- *In Situ Hybridization in the Allen Human Brain Atlas*

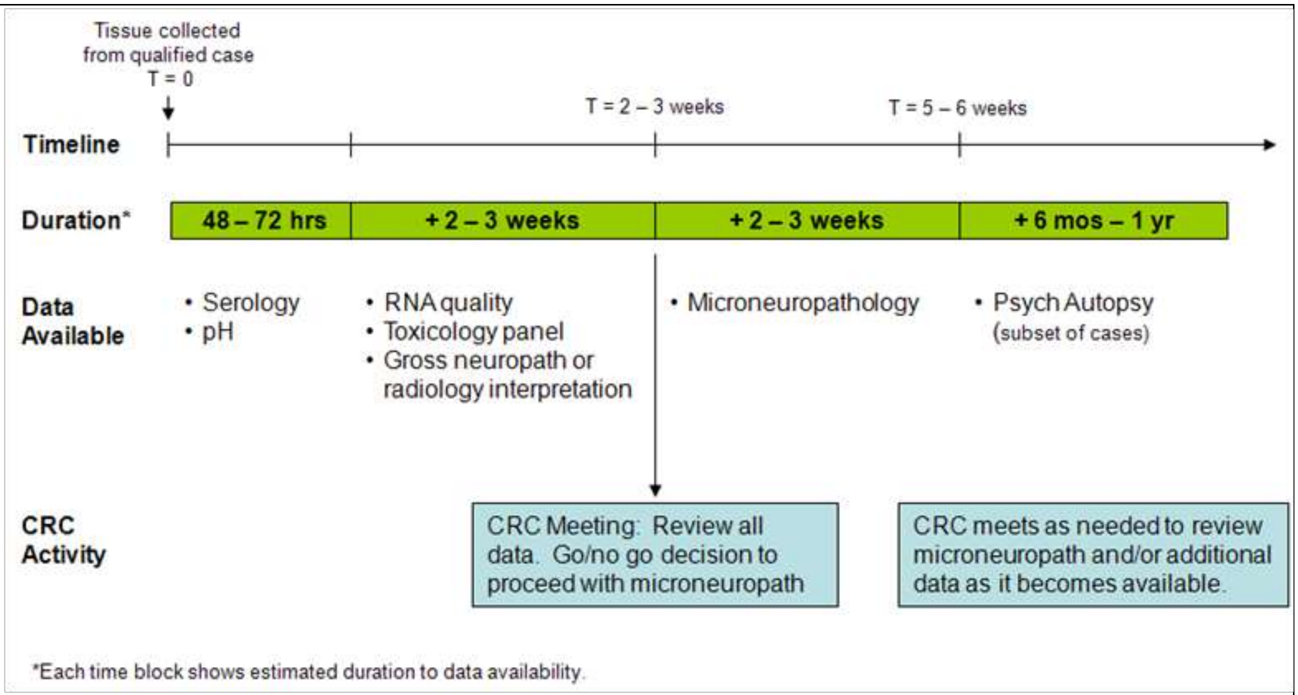

Figure 1. Timeline for receipt of case screening data and case review committee activities.

Table 1. Summary of case screens and quality control tests and criteria.

Test	Description	Passing Criteria
**Serology**	A safety precaution to evaluate blood serum for presence of antigens or antibodies for Hepatitis B, Hepatitis C or HIV1/HIV2.	Negative for all three tests.
**pH**	Measured in brain tissue homogenate and/or cerebrospinal fluid (CSF). Low pH levels are correlated with poor RNA quality.	pH ≥ 6.0
**RNA quality**	Assessed using Bioanalyzer-generated RNA Integrity Number (RIN) and Bioanalyzer electropherograms for 18s/28s ratios.	RIN ≥ 6.0, RNA amount ≥ 50ng, no obvious RNA degradation, no noticeable DNA or other contamination.
**Toxicology**	Postmortem blood is assessed for presence and concentration (when possible) of a broad range of therapeutic drugs and drugs with abuse potential.	Absence of drugs prescribed for neuropsychiatric disorders; absence of drugs at toxicologically significant levels (as reported by testing lab).
**Gross neuropathology**	Assessment of brain for gross morphological abnormalities indicating neuropathology (e.g. stroke, tumor, atrophy) by a radiologist using MRI data or by a pathologist using digital images of fresh brain sections.	'Normal' assessment by consulting radiologist or pathologist.
**Microneuropathology**	Analysis of histologically stained tissue sections to assess microscopic indications of pathology such as local ischemic events, abnormal levels of amyloid plaques or neurofibrillary tangles, or indications of abnormal cell morphology.	'Normal' assessment by consulting pathologist.

Table 2. Donor profile: H0351.1009.

Donor H0351.1009 – Microarray Survey, Neurotransmitter Study (ISH)	
**Tissue Receipt Date**	2/8/2011
**Sex**	Male
**Age**	57 years
**Race/Ethnicity**	Caucasian
**Handedness**	Cross-dominant
**Postmortem Interval**	25.5 hours (estimated time of death to time that tissue is frozen)
**Serology**	Pass
**Toxicology**	Positive for caffeine and theobromine, at levels usually not toxicologically significant
**Tissue pH**	6.9 (measured in frontal pole)
**RNA Quality**	Pass
**Neuropathology**	Gross pathology: NormalMicroneuropathology: Normal
**Tissue Received**	12 left hemisphere 1 cm cerebral slabs in coronal orientation<br>7 right hemisphere cerebral slabs in coronal orientation<br>7 cerebellar slabs in sagittal orientation; 1 cm thickness<br>1 brainstem, whole
**Additional Medical Information**	History of atherosclerotic cardiovascular disease
**Available Datasets**	See below

RNA Quality:

**Region Tested**	**RIN value (Mean ± SD)**
Frontal poles	6.4 ± 0.4
Occipital poles	6.1 ± 0.8
Cerebellum (left & right)	7.1 ± 0.5
Brainstem	5.6 ± 1.0

Available Datasets:

**MRI, DTI, Photodocumentation**	
MRI	Viewable online, available for download
Blockface images	Left hemisphere
**Histology**	
Nissl	Coronal left hemisphere, intact (6x8) and as mosaic reconstructions from 2x3 sections; individual 2x3 sections
SMI-32	2x3 sections
**Gene Expression**	
Microarray	~400 samples from left cerebral cortex, striatum and white matter structures, including controls and sample replicates
ISH	88 and 176 genes in subcortex and cortex, respectively; right hemisphere

Table 3. Donor profile: H0351.1010.

**Donor H0351.1010 – Neurotransmitter Study (ISH)**			
**Tissue Receipt Date**	2/23//2011		
**Sex**	Male		
**Age**	26 years		
**Race/Ethnicity**	Hispanic		
**Handedness**	Right		
**Postmortem Interval**	30 hours (estimated time of death to time that tissue is frozen)		
**Serology**	Pass		
**Toxicology**	Positive for atropine, caffeine, guaifenesin and theobromine, at levels usually not toxicologically significant		
**Tissue pH**	6.6 (measured in frontal pole)		
**RNA Quality**	Pass	**Region Tested**	**RIN value (Mean ± SD)**
		Frontal poles	6.4 ± 0.3
		Occipital poles	6.3 ± 0.6
		Cerebellum (left & right)	6.9 ± 0.4
		Brainstem	5.4 ± 0.0
**Neuropathology**	Gross pathology: Normal<br>Microneuropathology: Normal, hemosiderosis noted		
**Tissue Received**	16 left hemisphere 1 cm cerebral slabs in coronal orientation<br>8 right hemisphere cerebral slabs in coronal orientation<br>7 cerebellar slabs in sagittal orientation; 1 cm thickness<br>1 brainstem, whole		
**Additional Medical Information**	No clinically remarkable history.		
**Available Datasets**	**Histology**		
	Nissl	Neurotransmitter Study histology (2x3)	
	SMI-32	Neurotransmitter Study histology (2x3)	
	**Gene Expression**		
	ISH	88 and 176 genes in subcortex and cortex, respectively; right hemisphere	

Table 4. Donor profile: H0351.1012.

<table>
<tr><th colspan="4">Donor H0351.1012 – Microarray Survey, Neurotransmitter Study (ISH)</th></tr>
<tr><td>Tissue Receipt Date</td><td colspan="3">5/24/2011</td></tr>
<tr><td>Sex</td><td colspan="3">Male</td></tr>
<tr><td>Age</td><td colspan="3">31 years</td></tr>
<tr><td>Race/Ethnicity</td><td colspan="3">Caucasian</td></tr>
<tr><td>Handedness</td><td colspan="3">Right</td></tr>
<tr><td>Postmortem Interval</td><td colspan="3">17.5 hours (estimated time of death to time that tissue is frozen)</td></tr>
<tr><td>Serology</td><td colspan="3">Pass</td></tr>
<tr><td>Toxicology</td><td colspan="3">Positive for atropine, caffeine, ibuprofen and theobromine, at levels usually not toxicologically significant</td></tr>
<tr><td>Tissue pH</td><td colspan="3">6.8 (measured in frontal pole)</td></tr>
<tr><td rowspan="5">RNA Quality</td><td rowspan="5">Pass</td><td>Region Tested</td><td>RIN value (Mean ± SD)</td></tr>
<tr><td>Frontal poles</td><td>6.3 ± 0.3</td></tr>
<tr><td>Occipital poles</td><td>5.8 ± 0.3</td></tr>
<tr><td>Cerebellum (left & right)</td><td>6.9 ± 0.2</td></tr>
<tr><td>Brainstem</td><td>6.4 ± 0.0</td></tr>
<tr><td>Neuropathology</td><td colspan="3">Gross pathology: Normal
Microneuropathology: Normal</td></tr>
<tr><td>Tissue Received</td><td colspan="3">18 left hemisphere 1 cm cerebral slabs in coronal orientation
8 right hemisphere cerebral slabs in coronal orientation
8 cerebellar slabs in sagittal orientation; 1 cm thickness
1 brainstem, whole</td></tr>
<tr><td>Additional Medical Information</td><td colspan="3">Sudden cardiac arrest. Benign spindle cell proliferation and dystrophic calcification in temporal horn of lateral ventricle, ~5 mm, possibly an old choroid plexus infarct or degenerated xanthogranuloma.</td></tr>
<tr><td rowspan="9">Available Datasets</td><td colspan="3">MRI, DTI, Photodocumentation</td></tr>
<tr><td>MRI</td><td colspan="2">Viewable online, available for download</td></tr>
<tr><td>Blockface images</td><td colspan="2">Left hemisphere</td></tr>
<tr><td colspan="3">Histology</td></tr>
<tr><td>Nissl</td><td colspan="2">Coronal left hemisphere, intact (6x8) and as mosaic reconstructions from 2x3 sections; individual 2x3 sections</td></tr>
<tr><td>SMI-32</td><td colspan="2">2x3 sections</td></tr>
<tr><td colspan="3">Gene Expression</td></tr>
<tr><td>Microarray</td><td colspan="2">~500 samples from left cerebral, cerebellar and brainstem structures, including controls and sample replicates</td></tr>
<tr><td>ISH</td><td colspan="2">88 and 176 genes in subcortex and cortex, respectively; right hemisphere</td></tr>
</table>

Table 5. Donor profile: H0351.1015.

<table>
<tr><th colspan="4">Donor H0351.1015 – Microarray Survey</th></tr>
<tr><td>Tissue Receipt Date</td><td colspan="3">10/11/2011</td></tr>
<tr><td>Sex</td><td colspan="3">Female</td></tr>
<tr><td>Age</td><td colspan="3">49 years</td></tr>
<tr><td>Race/Ethnicity</td><td colspan="3">Hispanic</td></tr>
<tr><td>Handedness</td><td colspan="3">Right</td></tr>
<tr><td>Postmortem Interval</td><td colspan="3">30 hours (estimated time of death to time that tissue is frozen)</td></tr>
<tr><td>Serology</td><td colspan="3">Pass</td></tr>
<tr><td>Toxicology</td><td colspan="3">Positive for caffeine, at levels usually not toxicologically significant</td></tr>
<tr><td>Tissue pH</td><td colspan="3">6.9 (measured in frontal pole)</td></tr>
<tr><td rowspan="5">RNA Quality</td><td rowspan="5">Pass</td><td>Region Tested</td><td>RIN value (Mean ± SD)</td></tr>
<tr><td>Frontal poles</td><td>7.0 ± 0.2</td></tr>
<tr><td>Occipital poles</td><td>5.8 ± 1.2</td></tr>
<tr><td>Cerebellum (left & right)</td><td>7.5 ± 0.2</td></tr>
<tr><td>Brainstem</td><td>6.1 ± 0.4</td></tr>
<tr><td>Neuropathology</td><td colspan="3">Gross Pathology: Normal
Microneuropathology: Normal; modest numbers of hemosiderin laden macrophages noted in Virchow-Robin spaces in parietal and occipital lobes, mild arteriosclerosis</td></tr>
<tr><td>Tissue Received</td><td colspan="3">16 left hemisphere 1 cm cerebral slabs in coronal orientation
8 right hemisphere cerebral slabs in coronal orientation
8 cerebellar slabs in sagittal orientation; 1 cm thickness
1 brainstem, whole</td></tr>
<tr><td>Additional Medical Information</td><td colspan="3">Splenectomy, hypothyroidism treated with Levothroid</td></tr>
<tr><td rowspan="9">Available Datasets</td><td colspan="3">MRI, DTI, Photodocumentation</td></tr>
<tr><td>MRI</td><td colspan="2">Viewable online, available for download</td></tr>
<tr><td>Blockface images</td><td colspan="2">Left hemisphere</td></tr>
<tr><td colspan="3">Histology</td></tr>
<tr><td>Nissl</td><td colspan="2">Coronal left hemisphere, intact (6x8) and as mosaic reconstructions from 2x3 sections; individual 2x3 sections</td></tr>
<tr><td>SMI-32</td><td colspan="2">2x3 sections</td></tr>
<tr><td colspan="3">Gene Expression</td></tr>
<tr><td>Microarray</td><td colspan="2">~500 samples from left cerebral, cerebellar and brainstem structures including controls and sample replicates</td></tr>
</table>

Table 6. Donor profile: H0351.1016.

<table>
<tr><th colspan="4">Donor H0351.1016 – Microarray Survey, Neurotransmitter Study (ISH)</th></tr>
<tr><td>Tissue Receipt Date</td><td colspan="3">10/25/2011</td></tr>
<tr><td>Sex</td><td colspan="3">Male</td></tr>
<tr><td>Age</td><td colspan="3">55 years</td></tr>
<tr><td>Race/Ethnicity</td><td colspan="3">Caucasian</td></tr>
<tr><td>Handedness</td><td colspan="3">Right</td></tr>
<tr><td>Postmortem Interval</td><td colspan="3">18 hours (estimated time of death to time that tissue is frozen)</td></tr>
<tr><td>Serology</td><td colspan="3">Pass</td></tr>
<tr><td>Toxicology</td><td colspan="3">Positive for caffeine and theobromine, at levels usually not toxicologically significant</td></tr>
<tr><td>Tissue pH</td><td colspan="3">6.8 (measured in frontal pole)</td></tr>
<tr><td rowspan="5">RNA Quality</td><td rowspan="5">Pass</td><td>Region Tested</td><td>RIN value
(Mean ± SD)</td></tr>
<tr><td>Frontal poles</td><td>6.4 ± 0.5</td></tr>
<tr><td>Occipital poles</td><td>6.7 ± 0.7</td></tr>
<tr><td>Cerebellum (left & right)</td><td>7.4 ± 0.3</td></tr>
<tr><td>Brainstem</td><td>6.6 ± 0.2</td></tr>
<tr><td>Neuropathology</td><td colspan="3">Gross Pathology: Normal
Microneuropathology: Normal</td></tr>
<tr><td>Tissue Received</td><td colspan="3">16 left hemisphere cerebral slabs in coronal orientation
8 right hemisphere cerebral slabs in coronal orientation
9 cerebellar slabs in sagittal orientation; 1 cm thickness
1 brainstem, whole</td></tr>
<tr><td>Additional Medical Information</td><td colspan="3">Coronary artery atherosclerosis, prescriptions for clotting and high cholesterol.</td></tr>
<tr><td rowspan="9">Available Datasets</td><td colspan="3">MRI, DTI, Photodocumentation</td></tr>
<tr><td>MRI</td><td colspan="2">Viewable online, available for download</td></tr>
<tr><td>Blockface images</td><td colspan="2">Left hemisphere</td></tr>
<tr><td colspan="3">Histology</td></tr>
<tr><td>Nissl</td><td colspan="2">Coronal left hemisphere, intact (6x8) and as mosaic reconstructions from 2x3 sections; individual 2x3 sections</td></tr>
<tr><td>SMI-32</td><td colspan="2">2x3 sections</td></tr>
<tr><td colspan="3">Gene Expression</td></tr>
<tr><td>Microarray</td><td colspan="2">~500 samples from left cerebral, cerebellar and brainstem structures, including controls and sample replicates</td></tr>
<tr><td>ISH</td><td colspan="2">88 and 176 genes in subcortex and cortex, respectively; right hemisphere</td></tr>
</table>

Table 7. Donor profile: H0351.2001.

<table>
<tr><th colspan="4">Donor H0351.2001 – Microarray Survey</th></tr>
<tr><td>Tissue Receipt Date</td><td colspan="3">7/29/2009</td></tr>
<tr><td>Sex</td><td colspan="3">Male</td></tr>
<tr><td>Age</td><td colspan="3">24 years</td></tr>
<tr><td>Race/Ethnicity</td><td colspan="3">African American</td></tr>
<tr><td>Handedness</td><td colspan="3">Left</td></tr>
<tr><td>Postmortem Interval</td><td colspan="3">23 hours (estimated time of death to time that tissue is frozen)</td></tr>
<tr><td>Serology</td><td colspan="3">Pass</td></tr>
<tr><td>Toxicology</td><td colspan="3">Positive for atropine and caffeine, at levels usually not toxicologically significant</td></tr>
<tr><td>Tissue pH</td><td colspan="3">6.72</td></tr>
<tr><td rowspan="5">RNA Quality</td><td rowspan="5">Pass</td><td>Region Tested</td><td>RIN value
(Mean ± SD)</td></tr>
<tr><td>Frontal poles</td><td>7.1 ± 0.4</td></tr>
<tr><td>Occipital poles</td><td>6.5 ± 0.6</td></tr>
<tr><td>Cerebellum (left & right)</td><td>8.1 ± 0.4</td></tr>
<tr><td>Brainstem</td><td>7.1 ± 0.2</td></tr>
<tr><td>Neuropathology</td><td colspan="3">MRI-based Radiology Report: Normal
Microneuropathology: Normal</td></tr>
<tr><td>Tissue Received</td><td colspan="3">32 cerebral slabs in coronal orientation; 5 mm thickness
20 cerebellar slabs in sagittal orientation; 5 mm thickness
1 brainstem, whole</td></tr>
<tr><td>Additional Medical Information</td><td colspan="3">History of asthma</td></tr>
<tr><td rowspan="9">Available Datasets</td><td colspan="3">MRI, DTI, Photodocumentation</td></tr>
<tr><td>MRI</td><td colspan="2">Viewable online, available for download</td></tr>
<tr><td>DTI</td><td colspan="2">Viewable online, available for download</td></tr>
<tr><td></td><td colspan="2"></td></tr>
<tr><td colspan="3">Histology</td></tr>
<tr><td>Nissl</td><td colspan="2">Full coronal mosaic reconstructions from 2x3 sections; individual 2x3 sections</td></tr>
<tr><td colspan="3">Gene Expression</td></tr>
<tr><td>Microarray</td><td colspan="2">~1000 samples from > 300 left and right cerebral, cerebellar and brainstem structures, including controls and sample replicates</td></tr>
</table>

Table 8. Donor profile: H0351.2002.

<table>
<tr><th colspan="2">Donor H0351.2002 – Microarray Survey</th></tr>
<tr><td>Tissue Receipt Date</td><td>8/25/2009</td></tr>
<tr><td>Sex</td><td>Male</td></tr>
<tr><td>Age</td><td>39 years</td></tr>
<tr><td>Race/Ethnicity</td><td>African American</td></tr>
<tr><td>Handedness</td><td>Left</td></tr>
<tr><td>Postmortem Interval</td><td>10 hours (estimated time of death to time that tissue is frozen)</td></tr>
<tr><td>Serology</td><td>Pass</td></tr>
<tr><td>Toxicology</td><td>Positive for atropine, caffeine, lidocaine and monoethylglycinexylidide (MEGX) at levels usually not toxicologically significant</td></tr>
<tr><td>Tissue pH</td><td>6.86</td></tr>
<tr><td>RNA Quality</td><td>Pass
<table>
<tr><th>Region Tested</th><th>RIN value (Mean ± SD)</th></tr>
<tr><td>Frontal pole (left & right)</td><td>7.5 ± 0.2</td></tr>
<tr><td>Occipital pole (left & right)</td><td>7.1 ± 1.0</td></tr>
<tr><td>Cerebellum (left & right)</td><td>8.6 ± 0.6</td></tr>
<tr><td>Brainstem</td><td>7.3 ± 0.0</td></tr>
</table></td></tr>
<tr><td>Neuropathology</td><td>MRI-based Radiology Report: Normal; possible small pituitary adenoma
Microneuropathology: Normal; single neurofibrillary tangle in entorhinal cortex</td></tr>
<tr><td>Tissue Received</td><td>25 cerebral slabs in coronal orientation; 5 mm thickness
17 cerebellar slabs in sagittal orientation; 5 mm thickness; 1 broken and irreparable
1 brainstem, whole</td></tr>
<tr><td>Additional Medical Information</td><td>None known</td></tr>
<tr><td>Available Datasets</td><td>
<table>
<tr><th colspan="2">MRI, DTI, Photodocumentation</th></tr>
<tr><td>MRI</td><td>Viewable online, available for download</td></tr>
<tr><td>DTI</td><td>Viewable online, available for download</td></tr>
<tr><td>Blockface images</td><td>Left and right hemispheres</td></tr>
<tr><th colspan="2">Histology</th></tr>
<tr><td>Nissl</td><td>Full coronal 6x8 sections and full coronal mosaic reconstructions from 2x3 sections; individual 2x3 sections</td></tr>
<tr><td>SMI-32</td><td>2x3 sections</td></tr>
<tr><th colspan="2">Gene Expression</th></tr>
<tr><td>Microarray</td><td>~1,000 samples from > 300 left and right cerebral, cerebellar and brainstem structures, including positive controls and sample replicates</td></tr>
</table></td></tr>
</table>

Table 9. Donor profile H0351.2003 .

<table>
<tr><th colspan="4">Donor H0351.2003 – Subcortex Study</th></tr>
<tr><td>Tissue Receipt Date</td><td colspan="3">4/1/2010</td></tr>
<tr><td>Sex</td><td colspan="3">Female</td></tr>
<tr><td>Age</td><td colspan="3">48 years</td></tr>
<tr><td>Race/Ethnicity</td><td colspan="3">Caucasian</td></tr>
<tr><td>Handedness</td><td colspan="3">Right</td></tr>
<tr><td>Postmortem Interval</td><td colspan="3">24 hours (estimated time of death to time that tissue is frozen)</td></tr>
<tr><td>Serology</td><td colspan="3">Pass</td></tr>
<tr><td>Toxicology</td><td colspan="3">Positive for caffeine and theobromine at levels usually not toxicologically significant, acetone (1.6 mg/dL) consistent with low level fasting or diabetes.</td></tr>
<tr><td>Tissue pH</td><td colspan="3">6.65</td></tr>
<tr><td rowspan="5">RNA Quality</td><td rowspan="5">Pass</td><td>Region Tested</td><td>RIN value (Mean ± SD)</td></tr>
<tr><td>Frontal pole (left & right)</td><td>5.9 ± 0.7</td></tr>
<tr><td>Occipital pole (left & right)</td><td>7.7 ± 0.4</td></tr>
<tr><td>Cerebellum (left & right)</td><td>8.2 ± 0.3</td></tr>
<tr><td>Brainstem</td><td>7.5 ± 0.1</td></tr>
<tr><td>Neuropathology</td><td colspan="3">MRI-based Radiology Report: Normal; incidental 4mm angioma in left thalamus
Microneuropathology: Normal; moderate arteriosclerosis and perivascular hemosiderosis.</td></tr>
<tr><td>Tissue Received</td><td colspan="3">6 cerebral slabs in coronal orientation, anatomy-based cuts
10 cerebellar slabs in sagittal orientation, average thickness: 7 mm</td></tr>
<tr><td>Additional Medical Information</td><td colspan="3">Enlarged heart, history of sleep apnea and morbid obesity</td></tr>
<tr><td rowspan="10">Available Datasets</td><td colspan="3">MRI, DTI, Photodocumentation</td></tr>
<tr><td>MRI</td><td colspan="2">Available for download</td></tr>
<tr><td>DTI</td><td colspan="2">Available for download</td></tr>
<tr><td colspan="3">Histology</td></tr>
<tr><td>Nissl</td><td colspan="2">2x3 sections</td></tr>
<tr><td>AchE</td><td colspan="2">2x3 sections</td></tr>
<tr><td>Cytochrome Oxidase</td><td colspan="2">2x3 sections</td></tr>
<tr><td colspan="3">Gene Expression</td></tr>
<tr><td rowspan="2">ISH</td><td colspan="2">Right hypothalamus/amygdala: 10 genes</td></tr>
<tr><td colspan="2">Left subcortical region extending from head of caudate nucleus posteriorly to the posterior aspect of the substantia nigra: 55 genes</td></tr>
</table>

Table 9. Donor profile: H0372-006.

Donor H0372-006 – Subcortex Study			
**Tissue Receipt Date**	12/04/2009		
**Sex**	Male		
**Age**	44 years		
**Race/Ethnicity**	Caucasian		
**Handedness**	Right		
**Postmortem Interval**	24 hours (estimated time of death to time that tissue is frozen)		
**Serology**	Pass		
**Toxicology**	Positive for atropine, caffeine, lidocaine, theobromine, and dextro/levo-methorphan; at levels usually not toxicologically significant		
**Tissue pH**	6.85		
**RNA Quality**	Pass	**Region Tested**	**RIN value**
		Frontal pole (left & right)	7.4
		Occipital pole (left & right)	6.3
		Cerebellum (left & right)	Not sampled
		Brainstem	6.0
**Neuropathology**	MRI-based Radiology Report: Normal<br>Microneuropathology: Normal		
**Tissue Received**	4 cerebral slabs in coronal orientation<br>Slab thickness: 3.25 - 3.5 mm		
**Additional Medical Information**	Flu-like symptoms prior to death		
**Available Datasets**	**MRI, DTI, Photodocumentation**		
	MRI	Available for download	
	DTI	Available for download	
	**Histology**		
	Nissl	2x3 sections	
	AchE	2x3 sections	
	Cytochrome Oxidase	2x3 sections	
	**Gene Expression**		
	ISH	Right hypothalamus/amygdala: 10 genes<br>Left subcortical region extending from head of caudate nucleus posteriorly to the posterior aspect of the substantia nigra: 55 genes	
