# Supplementary material for: Human brain gene expression profiles of the cathepsin V and cathepsin L cysteine proteases, with the PC1/3 and PC2 serine proteases, involved in neuropeptide production
Source: Heliyon. 2018 Jul 3;4(7):e00673. doi: 10.1016/j.heliyon.2018.e00673 (PMC6037879; doi:10.1016/j.heliyon.2018.e00673)
Supplement: Technical White Paper _spl_2 — Technical white paper #2 “Microarray Survey” October 2013 v.7. Available for download at https://help.brain-map.org/display/humanbrain/Documentation. [file mmc3.pdf]

ALLEN **Human Brain Atlas**

TECHNICAL WHITE PAPER: MICROARRAY SURVEY

The Allen Human Brain Atlas is a publicly available online resource of gene expression information in human brain. Comprising multiple datasets from various projects characterizing gene expression in human tissue, one key component is an 'all genes, all structures' survey that generated genome-wide microarray-based gene expression profiles in human brain with accompanying anatomic and histologic data. These data were further processed and integrated into a resource with the following features:

- Navigable magnetic resonance image (MRI) data for visualization of brain areas sampled for gene expression profiling by microarray.
- A high-resolution histological dataset for visualization of brain tissue at the microscopic level.
- A hierarchical anatomic naming system (an ontology) that is referenced and viewable from various data modalities to provide anatomic context for histological and gene expression data.
- Search functions that allow users to search the database by anatomic region, probe, or gene.
- Expert neuroanatomic annotation of brain structures and delineations of specific areas sampled for microarray analysis.
- Downloadable microarray-based gene expression profiles for brain regions systematically sampled throughout the brain. Each gene expression profile contains information for over 62,000 gene probes with 93% of known genes represented by at least 2 probes.
- Gene expression data viewers that are color-coded and organized by ontology to allow comparison of gene expression data by anatomic structure and gene probe.
- Differential expression queries for statistical comparison of microarray-based gene expression profiles among pre-selected structure sets.

This white paper describes detailed methods and processes used in the creation of this microarray data resource. In addition to microarray datasets, gene expression data using an *in situ* hybridization (ISH) method and presented as cellular resolution images are included and accessible in the Allen Human Brain Atlas. The methods and processes for ISH data generation are described in a separate technical white paper (see the *In situ Hybridization in the Allen Human Brain Atlas* white paper).

PROCESS OVERVIEW

The central challenge to the creation of an 'all genes – all structures' survey of the entire human brain was the development of a process that enabled identification, delineation and sampling of specific anatomic structures for microarray analysis, and that also allowed the mapping of this sampling and resulting data back into three-dimensional neuroanatomic space. The process involved multiple brain and tissue subdivision steps, with collection of anatomic and histological information at each partitioning level, where the resolution of the anatomic and histological information increased as the tissue became further subdivided, ultimately resulting in delineation and dissection of small tissue samples for microarray analysis. A schematic of this process is

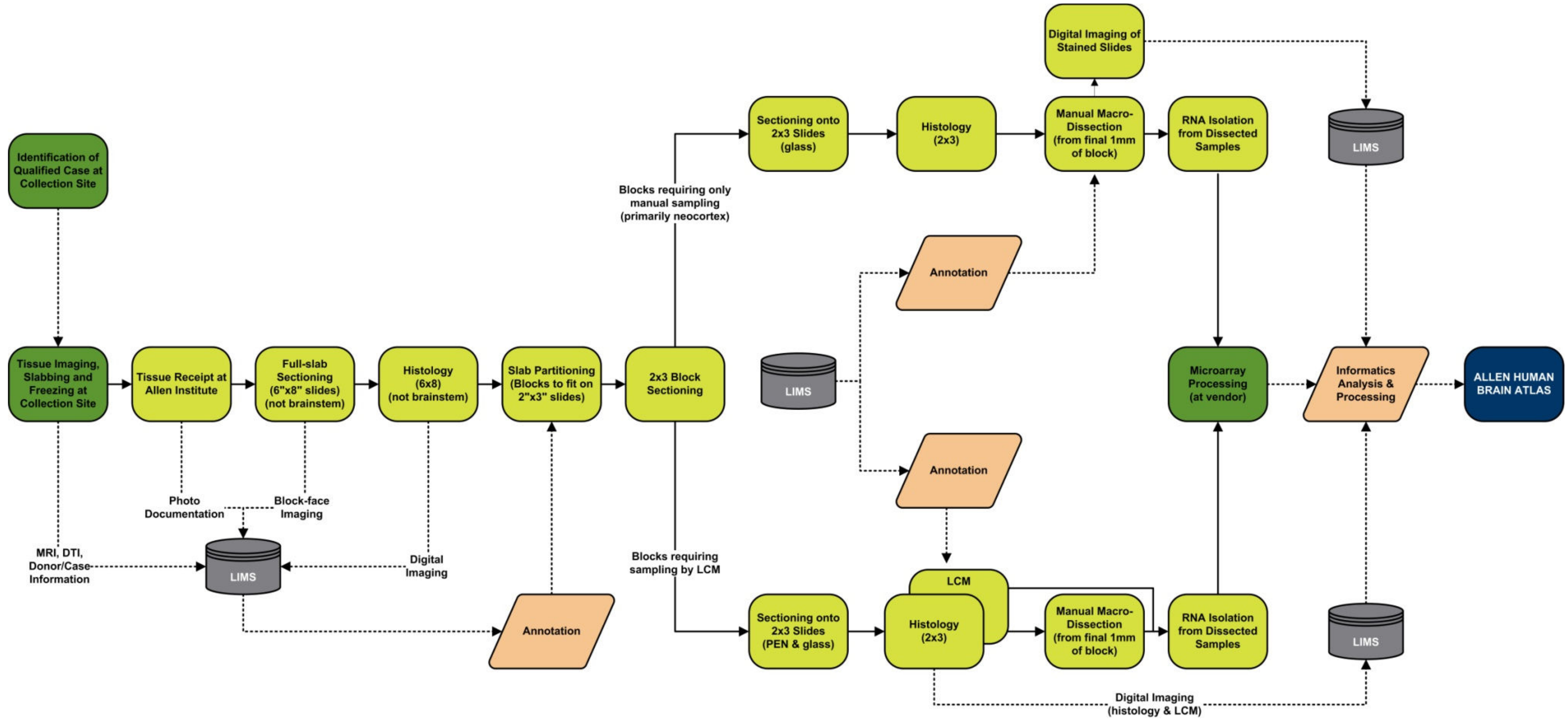

Figure 1. Process schematic of primary steps in the creation of the whole brain microarray survey of the Allen Human Brain Atlas.
LIMS is the Laboratory Information Management System in which all data are tracked and processed for presentation of data online.

shown in Figure 1; and a summary of the quality control (QC) steps throughout the process is provided in Appendix A. On a high level, the steps were as follows:

1. Initial tissue collection and processing occurred at independent tissue banks or tissue repositories, and resulted in fresh frozen tissue slabs shipped to the Allen Institute for further processing.
2. After successful screening and QC of the tissue, a limited set of large format histology data was collected from each tissue slab with 4.65 µm/pixel digital image resolution. A minimum specified amount of tissue in each slab was saved for the next processing step.
3. Each tissue slab was then subdivided into smaller tissue blocks categorized according to whether they contained primarily cortical or subcortical brain structures. These tissue blocks were sectioned for histology data with a final digital image resolution of 1 µm/pixel. If the blocks contained subcortical structures, additional sections were collected onto membrane slides that would allow laser microdissection (LMD) of these structures. A minimum specified amount of tissue in each block was saved for the next processing step.
4. Anatomically defined samples were collected for microarray analysis by either manual macrodissection of the remaining tissue from each block (cortical and some subcortical structures) or by laser-based microdissection (subcortical and brainstem areas).
5. Tissue samples collected for microarray analysis were processed for RNA isolation, quantification, and normalization.
6. RNA samples that passed QC were sent for microarray analysis by an independent service company, Beckman Coulter Genomics.
7. Data returned by Beckman Coulter Genomics were QC'd, normalized and analyzed prior to inclusion in the Allen Human Brain Atlas dataset.

Throughout the process described above, anatomic data (MR, blockface images, histology) were annotated to enable the collection of anatomically defined samples for microarray. Overall, the goal was to collect approximately 500 samples from each hemisphere, representing all structures within the brain in approximate proportion to the volumetric representation of each cortical, subcortical, cerebellar and brain stem structure. For two brains (H0351.2001 and H0351.2002), samples were collected from both hemispheres (cerebral, cerebellar and both sides of brainstem). Otherwise, samples for microarray were collected from the left cerebral and cerebellar hemispheres and left brainstem.

The following descriptions are divided into three main sections:

- Tissue Collection and Processing: provides information about tissue acquisition and screening, specific methods and anatomic criteria used to section and subdivide tissue, including sampling for microarray, and methods for RNA isolation and processing;
- Data Generation: provides specific methods for MRI, histological staining, and microarray experimental design and execution;
- Data Processing: describes processing steps required after data generation to include integrated data modalities in the online resource. This includes digital imaging of histological data, microarray data quality control and processing, and methods for anatomic visualization and mapping.

TISSUE COLLECTION AND PROCESSING

Postmortem Tissue Acquisition and Screening

Postmortem brain from males and females between 18 and 68 years of age, with no known neuropsychiatric or neuropathological history ('control' cases) was eligible for inclusion in the Atlas. Consent from next-of-kin was obtained in all cases. A screening process was employed to validate, as much as possible, a 'control' diagnosis, as well as to qualify the tissue for cytoarchitectural integrity and acceptable RNA quality. In addition to evidence of known history of neuropathological or neuropsychiatric disease, cases with long-term illnesses or events that resulted in hypoxic conditions lasting more than an hour were also excluded. Recent literature indicates that hypoxia is one primary contributor to detrimental effects on RNA quality (e.g. Atz et al., 2007; Durrenberger et al., 2010). As a safety precaution, a serology screen was performed to exclude cases with Hepatitis B, Hepatitis C, or HIV-1/HIV-2 (ViroMed, Minnetonka, MN). Blood samples from each

case were submitted for toxicology screening (NMS Labs, Willow Grove, PA) to determine presence of medications that might indicate neuropathology or neuropsychiatric disorder, including substance abuse or addiction. Gross neuropathology reports were provided by radiologists based on MRI data or by pathology consultants based on digital images of fresh brain during initial dissection and processing. Microneuropathology assessment was performed by a pathology consultant who viewed selected large format tissue sections stained with hemotoxylin & eosin (H&E), thionin-based Nissl stain, and silver or Thioflavin-S to determine: (1) presence of local ischemic events; (2) evidence of plaques or tangles; or (3) other cytoarchitectural abnormalities. In each case, the selected tissue sections targeted specific regions in which these events or phenomena were likely to occur (see Table 1). All screening and validation data were reviewed by a Case Review Committee (CRC) consisting of Allen Institute staff and external advisors with varying expertise in imaging, genetics, and neuroanatomy. Figure 2 shows the general timing of screening information availability and CRC decision points. For additional donor information and greater detail on screening processes and pass/fail criteria, see *Case Qualification and Donor Profiles.*

Table 1. Anatomic regions routinely assessed for microneuropathology.

Anatomic regions for Microneuropathology Assessment	Section Orientation	Slide Size
At level of amygdala	Coronal	6x8
Frontal lobe at level of anterior temporal pole	Coronal	6x8
Dorsal half of substantia nigra/posterior hippocampus	Coronal	6x8
Posterior parietal lobe	Coronal	6x8
Occipital lobe (towards occipital pole)	Coronal	6x8
Cerebellum, lateral hemisphere, right	Sagittal	6x8
Cerebellum, lateral hemisphere, left	Sagittal	6x8

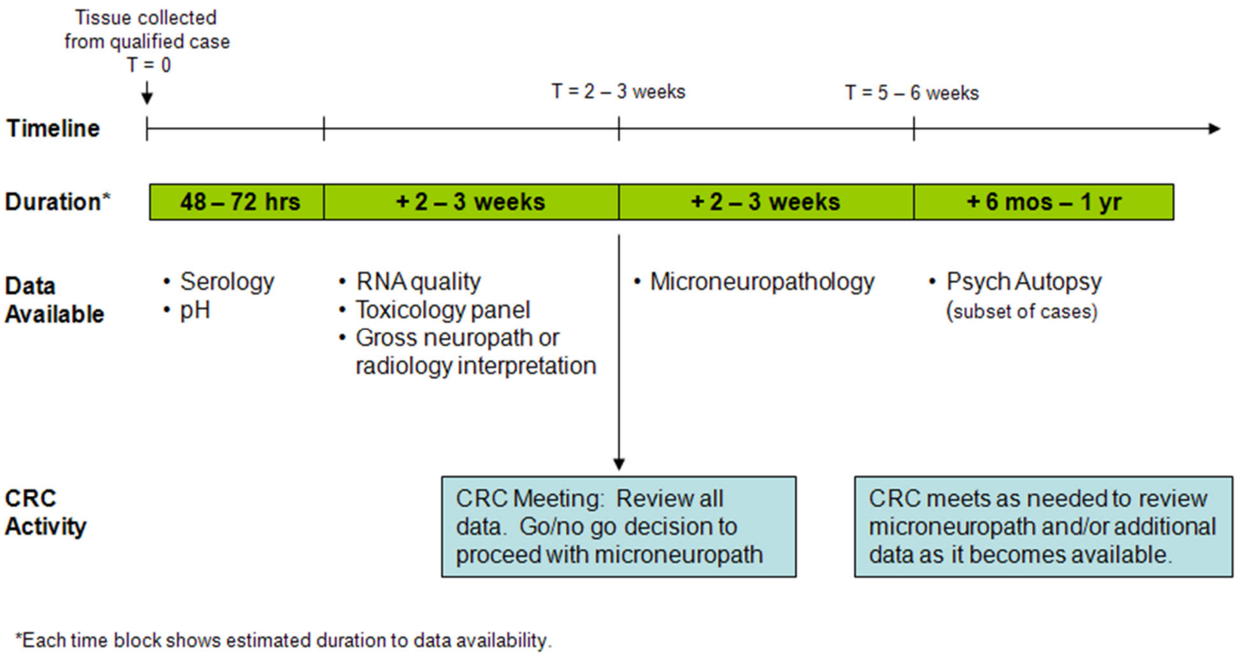

Figure 2. Timeline for availability of screening and quality control data used to qualify tissue for further processing for microarray. The Case Review Committee (CRC) was notified when data became available and met as needed depending on the complexity of data received.

Initial Tissue Dissection and Freezing

Initial collection, dissection and freezing of whole brain were performed at the NICHD Brain and Tissue Bank at the University of Maryland, Baltimore, or at the UCI Psychiatry Brain Donor Program and Functional Genomics Laboratory at the University of California, Irvine, Department of Psychiatry and Human Behavior. A multipart dissection and processing protocol was utilized, resulting in fresh frozen tissue comprising: (1) full cerebral slabs cut in coronal orientation; (2) cerebellar slabs cut in sagittal orientation; and (3) whole brainstem. Slab thicknesses ranged from ~ 0.5 – 1 cm at the time of dissection. This slabbing scheme was established to acquire the most recognizable histology results to define anatomic regions for microarray analysis.

The dissection and freezing protocol involved a number of detailed steps. Briefly, upon completion of MRI (see 'DATA GENERATION - Magnetic Resonance Imaging' below), the whole brain was chilled to approximately 4°C and dissected by separating the cerebellum and brainstem from the cerebrum. A small sample of the frontal pole was taken for pH measurement. The brainstem was then removed from the cerebellum with a transverse cut just dorsal to the pons. The brainstem was immediately placed on a Teflon freezing plate and frozen slowly to avoid cracking.

In the meantime, the cerebrum and cerebellum were prepared for dissection by encasing each structure separately in alginate (Cavex BV, Holland) and chilling the alginate to just above freezing. For some brains, the cerebrum was first hemisected into left and right hemispheres prior to embedding in alginate. Typically used for dental impressions, alginate was used here to preserve overall brain shape during the slabbing process. Liquid alginate was prepared by combining 250 g of alginate powder with 2L of ultra pure water in a blender. The cerebellum or cerebrum was placed in a structure-specific molded cavity created with a brain model (Global Technologies, Sunrise, FL) partially submerged in liquid alginate. Once the alginate hardened the tissue was placed in the molded cavity in place of the model, covered with additional liquid alginate to completely encase the tissue and placed in 4°C or -80°C to cool. Once the alginate cooled sufficiently and the tissue became more firm, the cerebellum and cerebrum were cut into approximately 0.5 – 1 cm thick slabs using a metal apparatus custom-designed to ensure consistency of slab thickness. The apparatus included a sliding platform on which the alginate-encased tissue was placed. For each slab, the platform was advanced in 0.5 or 1 cm increments and tissue was cut using a disposable stainless steel dissection knife (Mopec, Oak Park, Michigan). A knife guide affixed to the apparatus was used to control the path of the knife through the tissue and alginate. Tissue slabs were frozen immediately after cutting by controlled immersion in a bath of dry ice and isopentane. Each slab was placed between two metal plates during freezing to maintain flat surfaces in the cutting plane. Each frozen slab was stored separately in a labeled vacuum-sealed plastic bag at -80°C prior to shipping to the Allen Institute.

Frozen slabs were individually placed in 6"x8" padded envelopes to guard against breakage, snugly packed in a polystyrene foam box containing dry ice and shipped via overnight courier to the Allen Institute. Upon receipt, slabs were photographed and inspected for uniformity of slab thickness, anatomic plane of slabbing, and possible damage such as cracks or breaks in the tissue. Following inspection, slabs were stored at -80°C until future use. Photographic images taken at the time of slabbing and tissue receipt were reviewed to assess gross brain morphology, and symmetry and consistency of the cutting plane. Issues were documented for review and discussion by the CRC.

Large Format Sectioning and Blockface Imaging

In preparation for large format sectioning, frozen coronal and sagittal slabs were embedded and surrounded in mounting medium (OCT or CMC), resulting in flat frozen rectangular slabs comprising mounting media surrounding centrally embedded tissue. This process was done on a metal platform set on dry ice so that the tissue stayed frozen at all times. Care was taken to level the metal platform and tissue as much as possible. Aluminum bars were used as a frame to contain the media when first applied around the tissue and were removed as soon as the edges of the media solidified. Once all of the media solidified, embedded slabs were stored at -20°C for at least 24 hours prior to sectioning.

Large format coronal and sagittal tissue sections were placed on large glass slides (6"x8") for histological staining using a tape transfer method. Using OCT, an embedded slab was mounted on the leveled chuck of a

Reichert-Jung Cryo-Polycut or Leica CM3600(-18°C to -20°C at the chuck) and sectioned with a wedge-shaped steel blade (Leica) until the top layer of mounting media was removed. Coronally cut cerebral slabs were mounted with the anterior surface facing up while sagitally cut cerebellar slabs were mounted with the anatomic left-facing surface facing up. When a full tissue section was achieved, blockface imaging and collection of tissue sections for histology commenced. When it was not possible to obtain a full tissue section due to inconsistencies in tissue flatness, sections were taken at a tissue depth that allowed further processing of the slab for microarray sampling. Tissue sections for histology (25 or 50 μm thick) were placed on chilled (-11°C to -13°C) glass slides coated with Solution A and Solution B (Instrumedics) pretreatment and adhesive, respectively, using Macro-Tape-Transfer System (Instrumedics). Tape flags cut to be slightly longer than the embedded slab were adhered to the tissue surface and smoothed with a brush to eliminate air bubbles. The tape was used to pick up the tissue section after it was cut at approximately half-speed (setting 10 on a 1 – 20 scale). The flag and tissue were then placed tissue side down onto the surface of a glass slide. A brush was used to smooth the tissue and eliminate air bubbles. Tissue was adhered to the slide using a UV polymerizer placed over the glass slide for ~ 2 x 30 sec flashes. After removal of the tape, tissue slides were allowed to dry at room temperature and were subsequently stored at -20°C until histological staining.

Blockface digital images were obtained after every histology tissue section was collected using a Canon EOS Rebel XSi camera with a Canon EF 50 mm macrolens and Canon MT-24EX flash. The camera was mounted in a fixed position to maintain image consistency. Blockface images were used for annotation of anatomic regions and for mapping histological and gene expression information into 3-D MRI space.

Slab Partitioning

Slab partitioning was done after large format sectioning to subdivide full coronal, single hemisphere or full sagittal tissue slabs into pieces that could be further processed for histology data and microarray sampling using smaller (2"x3") microscope slides. These smaller sizes allowed the use of existing high-throughput histology equipment and processes for increased rate of data generation and higher image resolution of histological data relative to processes for 6"x8" data generation.

Each slab was partitioned on a flat metal dissection table housed in a modified temperature-controlled cryostat (-15°C). The slab was placed on a pre-chilled Teflon dissection board and subdivided using a steel blocking blade (Brain Research Laboratories, Newton, MA) and disposable sterile surgical blades (#11, #15, and #21, Feather Safety Razor Company, Osaka, Japan). A partitioning diagram was used to guide the placement of each cut into the slab. This diagram was drawn on the last blockface image collected for each slab, with cutting lines placed so that: (1) all resulting pieces fit onto 2"x3" slides; (2) cuts through cortex were perpendicular through all cortical layers (i.e. there were no transverse cuts separating cortical layers); (3) each 2x3 block contained mostly cortical or mostly subcortical structures; (4) subcortical structures were kept intact as much as possible (cuts through the putamen were almost always necessary); and (5) sufficient tissue support was maintained around periventricular structures in order to minimize tissue warping during processing. Each piece was categorized into one of three subtypes for future

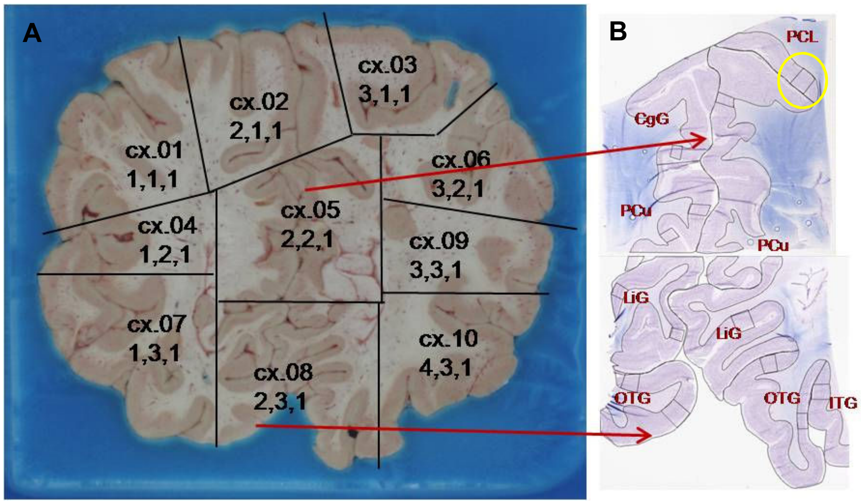

Figure 3. Representative slab partitioning diagram (A) and annotated Nissl image (B). The partitioning diagram shows cx block designations and x, y, z coordinates within the slab to assist in later reconstruction efforts. The Nissl image shows cortical delineations of major gyri labeled according to the Atlas ontology. Red arrows point to corresponding anatomic features in the Nissl images relative to the partitioning diagram. The yellow circle indicates a macrodissection site outlined on the Nissl image as a square shape. Multiple macrodissection sites can be seen in these Nissl images.

processing based on being either (1) primarily cortical (cx), (2) subcortical with small or oddly shaped structures (s1), or (3) subcortical with large structures (s2). A representative partitioning diagram (Figure 3A) shows cutting lines used for a full coronal slab with all cx blocks and associated grid coordinates (x, y, z) for each piece in that slab. Grid coordinates were later used for reconstruction of 2x3 pieces into a slab (see 'Anatomic Visualization and Mapping'). After subdivision, the full slab was reassembled and an image was taken and saved. Each resulting block was stored at -80°C in a separate plastic storage bag with a unique identifying label until sectioning. The entire partitioning process was video recorded to provide historical documentation of the process.

The brainstem did not require subdividing in this same manner because transverse sections already fit onto 2"x3" slides. However, the brainstem was subdivided in the transverse plane by cutting below the pons to create blocks less than 3 cm in length to be compatible with maximum clearance in the cryostat.

Processing and Sectioning of 2x3 Blocks

Slab partitioning resulted in 3 primary tissue type blocks based on anatomic areas contained within each block and the type of dissection required to collect microarray samples of the desired structures at the desired resolution. Cortical (cx) blocks contained primarily cortical regions for which manual macrodissections were done to collect tissue samples for microarray analysis. Subcortical s1 blocks contained primarily subcortical regions with oddly shaped structures or small substructures that required dissection by laser microdissection (LMD). For example, s1 blocks contained structures such as amygdala, hypothalamus and thalamus. Subcortical s2 blocks contained primarily subcortical regions that were designated for manual macrodissection, such as caudate, putamen and globus pallidus. Tissue sections were collected from each block for histological staining for anatomic structure identification and annotation. Histological data for each block type varied depending on the amount of anatomic information required to identify and dissect structures. Back-up slides were collected from each block in the event of process failure during staining. For s1 and brainstem blocks, half of the sections were collected onto PEN (polyethylene naphthalate) slides for downstream LMD. Tissue was sectioned such that ~ 1 – 2 mm of tissue remained from each block for macrodissection. Table 2 below provides a summary of data collected for each block type.

Table 2. Summary of dissection methods used and histological stain frequency for each 2x3 block type.

	Cortical (cx)	Subcortical 1 (s1)	Subcortical 2 (s2)	Brainstem
**Dissection Methods Used***				
**Manual Macrodissection**	Yes	Yes	Yes	No
**Laser Microdissection**	No	Yes	No	Yes
**Histological Stain Frequency**				
**Nissl**	every 250 µm	every 250 µm	every 250 µm	every 250 µm
**Silver or SMI-32****	every 1 mm	every 250 µm	every 250 µm	every 250 µm
**ISH*****	every 1 mm	every 1 mm	every 1 mm	every 1 mm
**PEN**	none	every 50 µm	none	every 50 µm

* Macrodissections were possible for every block except brainstem tissue. LMD was required for s1 blocks containing subcortical structures or cerebellar nuclei and brainstem, necessitating PEN slides for every other section.

** A silver stain was used in earlier stages of the project and was later replaced by SMI-32. The frequency was higher for s1, s2 and brainstem due to the higher resolution of anatomic information required to appropriately annotate structures.

*** Four gene markers, NEFH, GRIN1, GAD1 and GAP43 were characterized in s1, s2 and brainstem blocks; in cx blocks, NEFH and GRIN1 were assessed. The stain frequency listed is the frequency for each gene marker. These markers were used to provide additional information for anatomic annotations and delineation of sampling sites.

Frozen tissue samples were sectioned at 25 µm thickness in Leica CM3050 S cryostats (object temperature, -10°C; chamber temperature, -15°C). The sectioning plane was coronal for cerebral blocks, sagittal for cerebellar blocks and transverse for the brainstem. One section was placed on either a positively charged Superfrost Plus™ 2" x 3" microscope slide (Erie Scientific Co, Portsmouth, NH) or a 2" x 3" PEN membrane slide (Bartels & Stout, Issaquah, WA). All slides were uniquely barcoded and labeled for tracking purposes. After sectioning, tissue slides were processed as appropriate for each histological stain (see 'DATA

GENERATION - Histological Staining' below). PEN slides were not stained and were stored vacuum-sealed at -80°C until processed for LMD.

Microarray Sample Collection by Macrodissection or LMD

Collection of tissue samples that systematically represented all structures throughout the brain was done by either manual macrodissection for structures that were large enough to be easily identifiable, or by LMD, for smaller or oddly shaped structures that required microscopic visualization or handling for certainty regarding annotation or precision for collection of samples.

Manual Macrodissection

Areas sampled by macrodissection included cerebral and cerebellar cortex regions and large, regular nuclei within the subcortex. Areas were identified based on recognized neuroanatomy sources (see *Ontology and Nomenclature in the Allen Human Brain Atlas* white paper) and named according to the Atlas ontology. In the subcortex, macrodissection was limited to structures that were easily identified in non-stained tissue, such as the caudate and putamen. Specific areas for macrodissection were first identified by neuroanatomists using images of Nissl and silver -- or SMI-32-stained tissue sections immediately adjacent to the sampled tissue. Figure 3B shows an annotated Nissl section with outlines of specific cortical gyri and outlines (squares) of macrodissection sample sites. In the cortex, the size of the sample was approximately 5 linear mm along the length of the cortex and extending perpendicularly from the pial surface to the white matter. Macrodissection sites were placed on the bank of the sulcus, avoiding tangential cuts through the cortex in order to maintain proportional ratios of cell types across cortical samples. Between 1 – 4 macrodissection samples were taken per cortical gyrus, depending on the size and structure of the gyrus. Subcortical structures were extracted in their entirety.

Tissue was kept frozen throughout the macrodissection process using a temperature-controlled dissection table maintained at -15°C. All instruments and cutting surfaces were cleaned with 70% EtOH and RNaseZAP (Applied Biosystems/Ambion, Austin, TX) and were chilled to -15°C before use. Using the annotated Nissl and silver images as a guide, the indicated areas of tissue for collection were excised from the final 1 – 2 mm of the appropriate frozen 2x3 tissue block using a scalpel. Between 50 – 200 mg of tissue was excised during macrodissection depending on the region. Cortical sample sizes were 100 mg on average. Subcortical sample sizes were dependent on the size and portion of the nucleus, as well as position in the tissue. After dissection, each sample was immediately transferred using chilled forceps to an individual pre-weighed tube that was pre-chilled on dry ice. Care was taken to avoid unnecessary warming of the tissue or collection tube throughout the process. All surfaces and instruments were cleaned between dissections to prevent cross contamination. Tubes containing tissue were stored at -80°C until processing for RNA isolation. Nissl images with macrodissection sites were stored in a database and made available online.

Laser Microdissection (LMD)

Subcortical structures and cerebellar nuclei were sampled by LMD. For these structures, every other section was collected from appropriate tissue blocks and placed on a PEN slide designed for LMD. As with macrodissection, neuroanatomists drew structural boundaries on Nissl images to provide an LMD dissection guide that was also used to estimate the number and location of PEN slides required to meet minimum tissue requirements for each structure. Criteria for slide selection included systematic sampling throughout the structure using a minimum of 3 PEN slides in order to capture throughout the structure's length. In many cases, several different structures were collected from the same PEN slide or set of slides.

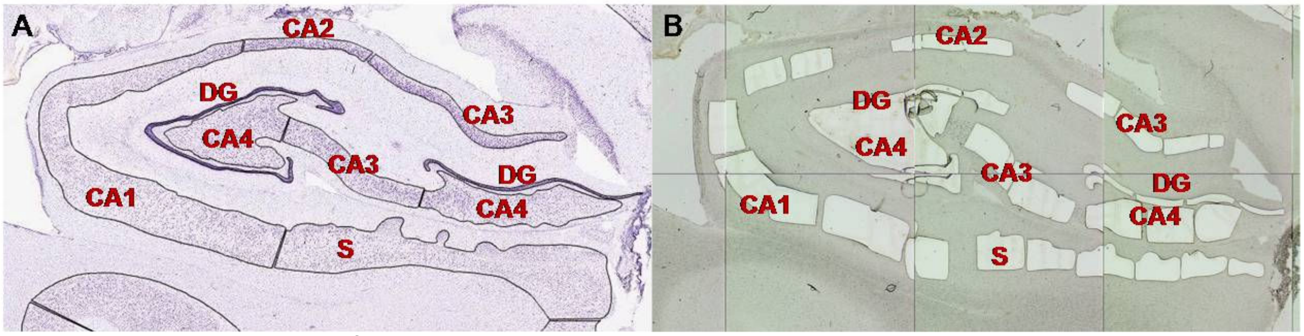

Figure 4. Annotated Nissl image of hippocampal structures (A) that were subsequently dissected by LMD (B). Multiple captures can be seen in various regions such as CA1 and CA3. All captures for a specific structure were collected in a single tube. For example, all CA3 captures were collected in one tube; all CA4 captures were collected in a separate tube. DG = dentate gyrus, S = subiculum.

Leica LMD6000 laser microdissection systems (Leica Microsystems, Wetzler, Germany) were used for laser dissection. Each system included an upright research microscope fitted with a diode laser and a CCD camera to acquire live images of slides. The scope and laser were controlled via a dedicated computer running Leica LMD software (v.6.6.2.3552). At the time of LMD dissection, the appropriate set of unstained PEN slides was removed from the freezer and allowed to equilibrate to room temperature for 30 minutes before being moved to a vacuum dessicator, where they were stored at all times unless on the LMD scope for tissue capture. PEN slides were stable for 8 hours at room temperature and humidity on the scope, or for one week at room temperature under vacuum, without detectable loss in RNA quality.

PEN slides were loaded individually onto the microscope and each structure to be dissected from the slide was identified by examining tissue images and referring to the Nissl dissection guide for landmarks. Specific tissue captures were hand-drawn using a tablet and stylus onto the slide image displayed on a computer monitor. The specified tissue was cut with the laser typically at full power using the 5x objective. Overview images of each slide taken at 1.25x before and after laser dissection were saved as documentation of specific dissections and are displayed online. Captured tissue was dropped into 8-tube PCR strips, where each tube was designated for one structure only and was prefilled with 100 µl of MELT enzyme cocktail (Ambion) prepared fresh daily with beta-mercaptoethanol (BME). Care was taken to avoid capturing tissue with sectioning or preservation artifacts that might affect RNA quality or data. Once sufficient tissue was collected from up to 8 structures, the strip was carefully unloaded from the LMD scope. Samples were vortexed on high for at least 10 minutes and volumized with additional MELT buffer + BME to bring each sample to 1.2mm^2/100µl. For the majority of samples, this resulted in 300 µl of final lysate. Lysates were then stored at -80°C until RNA isolation or for long-term storage.

RNA Isolation

Processes for RNA isolation differed slightly between macrodissected samples and LMD samples (see Table 3). Briefly, RNA from macrodissected samples was isolated using a guanidium-thiocynate-phenol-choloroform-based extraction method (TRI Reagent, Applied Biosystems) while RNA from LMD samples was isolated using the MELT system. For LMD samples, a DNAse step was necessary to remove DNA from the RNA isolates. RNA isolation was done for all samples using a magnetic bead-based system (MagMAX Express 96 Type 710, Applied Biosystems). This system had the advantage of allowing high-throughput processing in 96-well plates.

Table 3. Summary of RNA preparation methods for macrodissected and LMD tissue.

Capture Method	Tissue Amount	Sample per Lysate	Lysis Method	Homogenization	DNA removal	RNA Isolation
Macrodissection	50-200 mg	~10 mg	TRIZOL	Omni-Prep rotor-stator	Phase separation	Bead purification
LMD	36 mm^2 (0.9 mm^3)	12 mm^2 (0.3 mm^3)	MELT enzyme	Vortex	DNase	Bead purification

RNA Isolation from Macrodissected Tissue Samples
Tubes containing tissue were kept on dry ice and weighed to determine the mass of the dissected tissue. TRI was added to achieve a 50 mg/ml concentration, with at least 1 ml of TRI added for tissue samples under 50 mg. Up to 6 samples were lysed and homogenized at a time, using an Omni-Prep Homogenizer (Omni International, Kennesaw, GA). Tissue was lysed and homogenized for 1 – 2 min at 20,000 rpm. Once complete lysis was confirmed by visual inspection, tubes were incubated for 30 min in a 25°C water bath. Using this protocol, up to 100 samples could be lysed and homogenized in one hour. Following incubation, samples were phase separated to isolate RNA or were stored at -80°C.

Phase separation was performed in batches of 24 tubes. First, 500 µl of homogenized TRI lysate was added to labeled 1.5 ml tubes containing 50 µl of bromochloropropane (BCP). The contents were mixed by inversion, allowed to sit at room temperature for 5 minutes, and phase separated by centrifugation at 16,000xg at 4°C for 10 minutes. Two 100 µl aliquots of the acqueous phase from each tube were distributed into the appropriate wells on two different 96-well plates, designated as lysate (LYS) plates. These plates were either sealed and stored at -80°C with LN_2 backup, or were immediately processed for RNA isolation. The remaining TRI lysate that was not phase separated was stored at -80°C in bar-coded 2 ml tubes for future use.

RNA isolation was done using the MagMAX Express 96 (MME) system and reagents from a custom Ambion RNA isolation kit, similar to the MELT Total Nucleic Acid Isolation System by Ambion. LYS plates that were previously frozen were first allowed to thaw. All LYS plates received 45 µl isopropanol (100%, ACS grade) in each well; LYS samples were then pipette-mixed with the added isopropanol. Magnetic beads (10 µl) were then added to each well of the LYS plate. These magnetic beads were coated with a proprietary silica-like substance that bound nucleotides under conditions of high salt or low water concentrations. Two wash steps in Wash Solution 2 (150 µl per wash) were performed to remove any unbound or contaminating material, and RNA samples were eluted with 50 µl of Elution Buffer in new, bar-coded 96-well plates. Two µl of eluted RNA were removed and aliquoted into a 96-well PCR plate for quantification on a Nanodrop 8000 spectrophotometer (Thermo Scientific, Wilmington, DE). The plate containing the eluted RNA was sealed and stored in a -80°C freezer with LN_2 backup until normalization and QC.

RNA sample aliquots of 10 µl and 2 µl were normalized with nuclease free-water to 25 ng/µl and 7 ng/µl, respectively. RNA QC was performed using a Bionanalyzer (Agilent Technologies) with 1 µl of each RNA sample (7 ng/µl) loaded onto a Pico Chip. In general, samples with RNA integrity number scores (RIN; Schroeder et al, 2006) below 5.5, quantities below 50 ng, obvious RNA degradation or significant 28s ribosomal degradation, and/or with noticeable DNA or background contaminants did not pass QC, and were withheld from microarray analysis. Each sample was assessed individually and the final pass/fail call was made after weighing and evaluating all data. Frequent cross evaluation between QC personnel ensured consistency in ranking and assessment of data.

RNA Isolation from LMD Tissue Samples
RNA isolation from LMD tissue samples followed a process similar to that of macrodissected samples with an additional initial wash to remove any enzymes remaining from the MELT lysis step and a post-alcohol wash to remove any remaining DNA. All reagents used in this process were provided in the same custom RNA isolation kit from Ambion used for macrodissected samples. A mixture of Lysis/Binding Solution, Elution Buffer and Bead Mix (200 µl total) was added to each 100 µl sample of a LYS plate. Each mixture was washed in 150 µl Wash 1 Solution, washed in 150 µl Wash 2 Solution and treated with 50 µl DNase solution (1 µl Turbo DNase enzyme in 49 µl TurboDNase Buffer) followed by 100 µl RNA rebinding solution. RNA was finally eluted in 50 µl nuclease-free deionized water ($NFdH_2O$). Because LMD samples typically produced low yields of RNA, samples were immediately transferred to a SpeedVac (Savant IS110) and dried for 40 min. at room temperature to generate a final sample volume of 15 – 20 µl in water.

Two separate 100 µl lysates for each LMD sample went through RNA isolation. The separate RNA isolates were visually confirmed to be of comparable quality after running 1 µl on the Bioanalyzer Pico chip. As long as the RNA looked comparable, the RNA eluants were pooled (under witness), ran on another Pico chip, and

normalized. This was necessary because the microarray labeling protocol required samples to be at a concentration of 25 ng/µl, and a typical yield of LMD samples was 50 – 100 ng RNA from one lysate.

Pooled RNA was concentrated (Speedvac), quantitated (Nanodrop) and manually normalized to 25 ng/µl. While it was generally the case that RNA from LMD samples was of lower quality than RNA from macrodissected samples, the QC evaluation followed the same procedures. For failed LMD samples, the LMD process was repeated on stored backup PEN slides containing the appropriate structure.

Sample Preparation for Microarray Analysis

Throughout the course of this project, multiple batches of RNA samples in 96-well microarray plates (MAPs) were submitted to Beckman Coulter Genomics for microarray analysis. Because of this, specific experimental design choices were made to avoid the introduction of systematic bias in the microarray data. Design choices included initial selection of non-sequential tissue slabs for processing that allowed concomitant sampling of functionally and anatomically distinct areas, randomization of sample order for each MAP (batch) sent to Beckman Coulter Genomics, and randomization of technical replicate samples. Each MAP contained multiple experimental, control, and replicate samples. A total of 50 ng of RNA was prepared for microarray analysis by aliquoting 2 µl normalized RNA (25 ng/µl) to a bar-coded 96-well PCR plate. RNA samples were placed in random order on each plate. Each batch of RNA (~45 – 96 samples) included up to 9 technical replicates of samples. There were several types of technical replicates: (1) the same RNA in two different wells in the same microarray plate; (2) the same RNA in two different MAPs; and (3) the same sample lysate, resulting in an additional RNA isolate, in two different MAPs. Additionally, sample capture replicates were included at the tissue dissection level. About 13% of samples submitted for microarray were replicates. Every submitted MAP included at least two different reference RNA samples as positive controls and markers for batch effects. Initially, Ambion FirstChoice Human Brain Universal Reference RNA (normalized to 25 ng/µl) was used in addition to an internal reference RNA control made from pooling 300 cortical samples from the first brain. For each subsequent brain processed, an additional internal reference RNA control was created by pooling 300 samples from that brain. The internal reference RNA controls were run with each brain, comprising two wells containing the first brain's internal control and two wells containing the subsequent brain's internal control. Finally, a well containing NFdH_2O was provided as a negative control.

The final step in the preparation of samples to submit for microarray analysis was the addition of a selected set of External RNA Control Consortium (ERCC) transcripts to each well of each microarray plate. The ERCC is an ad hoc group that is developing and testing external RNA transcripts for use in assessment of technical performance of microarray or RT-PCR gene expression assays (ERCC, 2005). The use of ERCC transcripts in the Human Brain Atlas project was made possible through a Phase V testing opportunity from the ERCC. A set of 20 unique transcripts were selected for use, with each transcript assigned to every well of one column or every well of a one row in a 96-well plate. Each individual well thus contained a unique combination of two transcripts that provided a 'molecular barcode' that was tracked in the microarray data to ensure proper sample handling. ERCC transcripts (1 µl) were transferred to appropriate wells at the appropriate concentrations. The placement of ERCC transcripts was constant for all microarray plates submitted for analysis.

DATA GENERATION

Magnetic Resonance Imaging

Magnetic resonance images (MRI) were collected prior to dissection for anatomic visualization of each brain. In T1-weighted images, white matter voxels exhibit higher signal intensity than grey matter voxels. Conversely, in T2-weighted MRI images grey matter shows higher intensity than white matter. T2-weighted fluid attenuated inversion recovery (FLAIR) scans are helpful for visualization of lesions in white matter to detect pathology or ischemic changes present in the brain. Inversion recovery images are T1-weighted images often used to suppress the contribution of fat to the MRI signal. Diffusion tensor (DT) imaging techniques are typically used to examine white matter anatomy with respect to direction and local integrity of fibers, thereby providing information regarding overall connectivity of white matter tracts.

Various T1-weighted, T2-weighted, FLAIR and inversion recovery and DT images were acquired on 3T Siemens Magnetom Trio scanners (Erlangen, Germany) at the Department of Radiology, University of

Maryland School of Medicine and University of Maryland Medical Center, Baltimore, MD, or the University of California Irvine Medical Center, Irvine, CA. Specific scan sequences were as follows for each brain:

H0351.1009
- T1-weighted MPRAGE structural MRI with 0.98 x 0.98 x1 mm voxels, three-dimensional acquisition, three averages, TI = 900 ms, TR = 1900 ms, TE = 3.03 ms, 9° flip angle, image matrix = 256 x 256 x 176.
- T2-weighted images were taken in three-dimensions with 0.98 x 0.98 x 1 voxels, TR = 3200 ms, TE = 449 ms, 120° flip angle, image matrix = 256 x 256 x 192 voxels.
- FLAIR images acquired in 0.98 x 0.98 mm axial slices and 5 mm slices, TI = 2500 ms, TR= 10000 ms, TE= 73 ms, 150° flip angle, image matrix = 256 x 256 x 20 voxels.
- T2-weighted gradient echo images taken in axial slices with 0.72 x 0.72 mm and 5 mm slices, TR = 889 ms, TE = 18 ms, 60° flip angle, image matrix = 320 x 290 x 24 voxels.

H0351.1012
- T1-weighted MPRAGE structural MRI with 0.98 x 0.98 x1 mm voxels, three-dimensional acquisition, three averages, TI = 900 ms, TR = 1900 ms, TE = 3.03 ms, 9° flip angle, image matrix = 256 x 256 x 176.
- T2-weighted images were taken in three-dimensions with 0.98 x 0.98 x 1 voxels, TR = 3200 ms, TE = 449 ms, 120° flip angle, image matrix = 256 x 256 x 192 voxels
- FLAIR images acquired in 0.98 x 0.98 mm axial slices and 5 mm slices, TI = 2500 ms, TR= 10000 ms, TE= 73 ms, 150° flip angle, image matrix = 256 x 256 x 20 voxels.
- T2-weighted gradient echo images taken in axial slices with 0.72 x 0.72 mm and 5 mm slices, TR = 889 ms, TE = 18 ms, 60° flip angle, image matrix = 320 x 290 x 24 voxels.

H0351.1015
- T1-weighted MPRAGE structural MRI with 0.98 x 0.98 x1 mm voxels, three-dimensional acquisition, three averages, TI = 900 ms, TR = 1900 ms, TE = 2.63 ms, 9° flip angle, image matrix = 256 x 256 x 176.
- T2-weighted images were taken in three-dimensions with 0.98 x 0.98 x 1 voxels, TR = 3200 ms, TE = 449 ms, 120° flip angle, image matrix = 256 x 256 x 192 voxels
- FLAIR images acquired in 0.98 x 0.98 mm axial slices and 5 mm slices, TI = 2500 ms, TR= 10000 ms, TE= 73 ms, 150° flip angle, image matrix = 256 x 256 x 20 voxels.
- T2-weighted gradient echo images taken in axial slices with 0.72 x 0.72 mm and 5 mm slices, TR = 889 ms, TE = 18 ms, 60° flip angle, image matrix = 320 x 290 x 24 voxels.

H0351.1016
- T1-weighted MPRAGE structural MRI with 0.98 x 0.98 x1 mm voxels, three-dimensional acquisition, three averages, TI = 900 ms, TR = 1900 ms, TE = 2.63 ms, 9° flip angle, image matrix = 256 x 256 x 176.
- T2-weighted images were taken in three-dimensions with 0.98 x 0.98 x 1 voxels, TR = 3200 ms, TE = 449 ms, 120° flip angle, image matrix = 256 x 256 x 192 voxels
- FLAIR images acquired in 0.98 x 0.98 mm axial slices and 5 mm slices, TI = 2500 ms, TR= 10000 ms, TE= 73 ms, 150° flip angle, image matrix = 256 x 256 x 20 voxels.
- T2-weighted gradient echo images taken in axial slices with 0.72 x 0.72 mm and 5 mm slices, TR = 889 ms, TE = 18 ms, 60° flip angle, image matrix = 320 x 290 x 24 voxels.

H0351.2001
- T1-weighted MPRAGE structural MRI with 1 mm isotropic voxels, three-dimensional acquisition, three averages, TI = 900 ms, TR = 1900 ms, TE = 2.63 ms, 9° flip angle, image matrix = 256 x 192 x 256.
- T2-weighted images were taken in three-dimensions with 0.9 mm isotropic voxels, TR = 3210 ms, TE = 540 ms, 120° flip angle, image matrix = 224 x 256 x 160 voxels.
- DT images taken in 64 directions using an Echo Planar Imaging sequence taken in 2 mm axial slices with 1.875 x 1.875 mm in plane voxels, TR = 9300 ms, TE = 94 ms, 90° flip angle, image matrix = 128 x 128 x 68.
- FLAIR images acquired in 0.86 mm x 0.86 mm axial slices and 5 mm slices with a 1 mm gap, TI = 2500 ms, TR= 8000 ms, TE= 67 ms, 170° flip angle, image matrix = 208 x 256 x 26 voxels.
- T2-weighted gradient echo images taken in axial slices with 0.86 mm x 0.86 mm and 5 mm slices, TR = 613 ms, TE = 20 ms, 20° flip angle, image matrix = 256 x 256 x 26 voxels.

- Inversion recovery images taken with sagittal sections with 0.86 mm x 0.86 sagittal sections and 4 mm slices, TI = 185 ms, TR = 5523.2 ms, TE = 62 ms, 170° flip angle, image matrix = 248 x 256 x 33 voxels.

H0351.2002

- T1-weighted MPRAGE structural MRI with 1 mm isotropic voxels, three-dimensional acquisition, three averages, TI = 900 ms, TR = 1900 ms, TE = 2.63 ms, 9° flip angle, image matrix = 256 x 192 x 256.
- T2-weighted images were taken in three-dimensions with 0.9 mm isotropic voxels, TR = 3200 ms, TE = 535 ms, 120° flip angle, image matrix = 224 x 256 x 160 voxels.
- DT images taken in 64 directions using an Echo Planar Imaging sequence taken in 5mm axial slices with 1.875 x 1.875 mm in plane voxels, TR = 9300 ms, TE = 94 ms, 90° flip angle, image matrix = 128 x 128 x 24.
- FLAIR images acquired in 0.94 mm x 0.94 mm axial slices and 5 mm slices with a 1 mm gap, TI = 2000 ms, TR= 8000 ms, TE= 67 ms, 170° flip angle, image matrix = 208 x 256 x 24 voxels.
- T2-weighted gradient echo images taken in axial slices with 0.94 mm x 0.94 mm and 5 mm slices, TR = 665 ms, TE = 20 ms, 20° flip angle, image matrix = 256 x 256 x 24 voxels.
- Inversion recovery images taken with sagittal sections with 0.86 mm x 0.86 mm sagittal sections and 3 mm slices, TI = 185 ms, TR = 4230 ms, TE = 62 ms, 170° flip angle, image matrix = 248 x 256 x 25 voxels.

MRI scans were of either *in cranio* or *ex cranio* brains, depending on where the tissue was collected. *Ex cranio* brains were encased in alginate to provide stability and help the brain maintain shape as much as possible during scanning. De-identified MRI and DT data were electronically transferred in DICOM file format to the Allen Institute for further processing.

Histological Stains

The staining procedures described below were utilized for tissue sections on both 6x8 and 2x3 slides. In general, the protocols were similar for the different slide sizes except that 2x3 slides were processed in automated stainers (Leica Autostainer XL or Microm HMS 760X) whereas 6x8 slides were processed in custom-designed staining containers that held up to six 6x8 slides at a time and required manual movement of slides from one reagent to the next after the appropriate incubation time.

Hemotoxylin and Eosin (H&E)

Tissue sections from selected regions on large format 6x8 slides (see Table 1) were subjected to a regressive H&E stain (Culling et al., 1985) for microneuropathology assessment. Tissue was fixed with 10% neutral buffered formalin (NBF) 30 minutes – 2 hours after sectioning. Slides were then stored for up to two weeks prior to staining. Sections were stained with commercially prepared Harris hematoxylin (VWR), differentiated in 1% HCl in 70% ethanol, blued with 1% lithium carbonate and stained in 1% eosin Y in 1% aqueous calcium chloride. Sections were then dehydrated in a graded series of 50%, 70%, 95% and 100% ethanol, cleared in xylene and coverslipped with either DPX or CureMount mounting media.

Nissl

Nissl stained sections provided cytoarchitectural reference in 6x8 and 2x3 slide formats to help identify anatomic regions. After brain tissue was sectioned, slides were stored at 37°C for 1 – 5 days and were removed 5 – 15 minutes prior to staining. Sections were defatted with xylene or the xylene substitute Formula 83, and hydrated through a graded series containing 100%, 95%, 70%, and 50% ethanol. After incubation in water, the sections were stained in 0.213% thionin, then differentiated and dehydrated in water and a graded series containing 50%, 70%, 95%, and 100% ethanol. Finally, the slides were incubated in xylene or Formula 83, and coverslipped with the mounting agent DPX. After drying, the slides were analyzed microscopically to ensure staining quality. Slides that passed QC were stored at room temperature in slide boxes before being cleaned in preparation for digital imaging.

Modified Bielschowski Silver Stain

Silver staining (Garvey et al., 1987) was used to visualize axons on fresh frozen human tissue mounted on 2x3 and 6x8 slides. Silver staining was included in the microneuropathology screen to assess the possible presence of neurofibrillary tangles or amyloid plaques associated with dementia or Alzheimer's disease. After

brain tissue was sectioned, slides were stored in desiccator cabinets until staining. One day prior to staining, sections were fixed overnight in 10% NBF. The following day, sections were defatted with xylene or Formula 83, and hydrated through a graded series containing 100%, 95%, 70%, and 50% ethanol. After incubation in water, the sections were stained in 0.5% silver nitrate solution heated to 65°C. Following a wash and gum mastic step to stabilize the tissue, sections were placed in a 65°C silver nitrate developing solution for 25 min. Next, the sections were differentiated and dehydrated in water and a graded series containing 50%, 70%, 95%, and 100% ethanol. Finally, the slides were incubated in xylene or Formula 83, and coverslipped with the mounting agent DPX. After drying, the slides were analyzed microscopically to ensure staining quality. Slides that passed QC were stored at room temperature in slide boxes before being cleaned and scanned.

SMI-32
Immunohistochemical staining using SMI-32, an antibody that reacts with an epitope on non-phosphorylated neurofilament H proteins, was used to visualize cell bodies and processes to help delineate anatomic regions. Stored fresh frozen tissue sections on slides were taken out of storage at -80°C, equilibrated to room temperature and fixed with 100% ice-cold acetone. Sections were then rehydrated in 1X PBS with potassium, pH 7.4 (1:10 dilution of 10X PBS, Ambion, Austin, TX). Non-specific binding was blocked with 5% horse serum (Vector Laboratories, Burlingame, CA) in PBS and permeabilized with 0.3% Triton-X. Endogenous peroxidase activity was blocked in 3% hydrogen peroxide in methanol. Sections were then incubated in 1:1000 dilution of mouse anti-SMI-32 (Covance, Inc., Princeton, NJ) for 1 hr. Sections were rinsed in PBS–Tween 20 (0.0005%), then incubated in 1:100 biotinylated horse anti-mouse IgG secondary antibody (Vector Laboratories), rinsed in PBS-Tween, and incubated for 30 min in ABC (Vectastain, Vector Laboratories). The reaction product was visualized with 0.5% DAB (Sigma-Aldrich), activated with 0.003% hydrogen peroxide. Sections were dehydrated through graded alcohols, cleared with Formula 83 and coverslipped with DPX. Slides were QC'd and stored at room temperature prior to digital imaging.

In situ hybridization (ISH)
High-throughput colorimetric ISH methods are described in detail elsewhere (see Lein et al., 2007 and *In Situ Hybridization in the Allen Human Brain Atlas* white paper*)*. Fresh frozen tissue sections on slides were fixed in 4% PFA in PBS, acetylated, and dehydrated through graded alcohols. Endogenous peroxidase activity was blocked with 3% hydrogen peroxide in methanol. Digoxigenin (DIG)-labeled riboprobes were hybridized at 63.5°C for 5.5 hr, followed by stringency washes and a series of enzymatic reaction steps for detection and amplification of DIG signal. Sections were washed with EDTA, fixed in 4% PFA, and washed with acid alcohol (70% EtOH, adjusted to pH = 2.1 with 12 N HCl) to reduce background signal. Slides were coverslipped with Hydromatrix, QC'd and cleaned prior to digital imaging.

Microarray Data Generation

An Agilent 8x60K array, custom-designed by Beckman Coulter Genomics in conjunction with the Allen Institute, was used to generate microarray data. The array design included the existing 4x44K Agilent Whole Human Genome probe set supplemented with an additional 16,000 probes. At least two different probes were available for 93% of genes with EntrezGeneIDs (21,245 genes). Probes were located on different exons as much as possible when multiple probes were available for a gene. Other probes on the microarray were for transcripts with UCSC IDs (1,852 transcripts) and Agilent IDs (1,268 transcripts). A small set of probes mapped to contigs (253) and not to any well-defined transcript. An additional set of probes were included to overlap with the 1,000- and 60-gene sets that were characterized by ISH for the 1,000 Gene Survey in Cortex and the Subcortex Study, respectively, both of which are integrated into the Allen Human Brain Atlas. For the 60 genes of the Subcortex Study, a set of tiling probes was designed and included on the array. Finally, 40 probes (5 replicates per probe) specific to the ERCC transcripts were included for the sample tracking molecular bar-codes.

Total RNA in the amount of 50 ng per sample was sent to Beckman Coulter Genomics for processing on Agilent 8x60K gene expression arrays. Samples were processed using 1.5 µl of the 3 µl submitted (2 µl of 25 ng/µl total RNA and 1 µl ERCC barcodes). Total RNA samples underwent conversion to Cy3-labeled cRNA using Agilent's Ultra-Low Input Quick Amplification kit. E1A spike-in controls were added during the labeling reaction step as another control to measure labeling data quality after hybridization. Labeled cRNA samples were quantified on a Nanodrop and tested on the Bioanalyzer to assess cRNA spreads. Samples with cRNA

yields in excess of 600 ng and spreads in excess of 500 bp were of sufficient quality for hybridization onto the custom array chip (AMADID24915).

600 ng of labeled cRNA was applied to each array and subsequently hybridized and scanned according to Agilent's recommended procedures. The samples were processed in batches of ~45 to 96 samples. The following files for each array were delivered to the Allen Institute: the Agilent Feature Extraction (FE) output file[1], the MAGE-ML formatted file[2], and related Feature Extraction QC reports. The Bioconductor package (http://www.bioconductor.org) "Agi4x44PreProcess" was used to read Agilent FE output expression data for QC, normalization, and further analyses.

RNA-Sequencing Data Generation

RNA-Sequencing (RNA-Seq) data were generated for a selected set of 240 samples (120 from each brain) representing 29 cortical and subcortical regions matched across two brains (H0351.2001 and H0351.2002), using aliquots of the same total RNA isolates used to generate microarray data. Aliquots were from macrodissected tissue samples (see above 'RNA isolation from macrodissected samples'). RNA-Seq library preparation and sequencing were performed using Illumina sequencing technology (Expression Analysis, Durham, NC). In brief, cDNA libraries were constructed from total RNA (250 ng) using the Illumina Tru-Seq v2 preparation kit and subjected to 50 bp paired end sequencing using an Illumina HiSeq2000 platform to a target depth of 30MM reads per sample. Sequences were aligned to the genome using RNA-Seq by Expectation-Maximization (Li *et al.,* 2010) and transcripts defined using the knownGene table from the USCD Genome Browser (Meyer *et al.*, 2013; genome.ucsc.edu; hg19, Feb 2009). Summary expression levels for each gene and each isoform were calculated as counts and as transcripts per mission (TPM). These data are available as downloads in the Allen Human Brain Atlas application.

DATA PROCESSING

Microarray Data Processing

Initial Data Quality Control

Upon receipt, all microarray data were subjected to a standard set of QC steps that included recording and assessment of: (1) the 99% non-control probe signal, (2) visual inspection of array thumbnails, (3) %CV of non-control probes, (4) E1A control signal, and (5) outlier detection. All metrics are available in Agilent's Feature Extraction (FE) output.

Default Agilent GE1 QC thresholds were used as initial quality control criteria of the microarray data (Table 4). Any arrays with values outside of the threshold ranges were further evaluated. If an explanation for values outside of threshold could not be determined the array was failed. Furthermore, visual assessments of the spatial distribution of all outliers, spot finding of the four corners of the array and the histogram of signal plots were performed and recorded for each individual array. This was done to track both hybridization performance and RNA sample quality. (Figures can be found on pages 65-67 of Agilent Feature Extraction Software Reference Guide (v10.5).

Table 4. Evaluation metrics for initial microarray data quality control.

Metric Name	Value	Upper Limit	Lower Limit	Is Mandatory
AnyColorPrcntFeatNonUnif...	0.01	1.0	NA	FALSE
DetectionLimit	1.38	2.0	0.01	FALSE
absGE1E1aSlope	0.99	1.2	0.9	FALSE
gE1aMedCVProcSignal	4.30	8.0	NA	FALSE
gNegCtrlAveBGSubSig	-2.64	5.0	-10	FALSE
gNegCtrlAveNetSig	28.6	40.0	NA	FALSE

1. http://www.chem.agilent.com/Library/usermanuals/Public/G4460-90017_FE_10.5_Installation.pdf
2. http://www.mged.org/Workgroups/MAGE/mage-ml.html

gNegCtrlSDevBGSubSig	2.60	10.0	NA	FALSE
gNonCntrlMedCVProcSignal	4.72	8.0	NA	FALSE
gSpatialDetrendRMSFilter...	3.44	15.0	NA	FALSE

In addition, a heuristic detection rule, Inter-Array Connectivity (IAC) was designed to identify outlier candidates for each batch (Table 5). Samples identified as outliers were resubmitted for microarray data generation.

Table 5. Outlier detection based on Inter-Array Connectivity (IAC).

Sample Capture Method	When, IQD(IAC of batch)	Sample is called an outlier, IF
Macrodissection	IQD(IAC) ≥ 0.02	IAC(s) < 0.92
	IQD(IAC) < 0.02	IAC(s) < 0.92, s is in lowest 5% end
LMD	IQD(IAC) ≥ 0.02	IAC(s) < 0.80, s is in lowest 5% end
	IQD(IAC) < 0.02	Samples in lowest 5% end

IAC of a sample is the mean of correlation values between the sample and all other samples in the batch. Inter-Quartile Distance (IQD) of IAC is used to identify the range of the distribution of IAC.

ERCC transcripts were used to detect possible errors in sample handling related to mixing samples in wells or inadvertently loading an incorrect sample onto a particular array. Microarray data for ERCC specific probes were assessed using a heat map to track placement of samples. The quality control process included confirming that only the expected row and column spiked transcripts showed signal above background. Samples were failed if they did not contain exclusively the ERCC transcripts spiked into the RNA well. Figure 5 below shows an example heat map in which a sample cross-contamination event was detected.

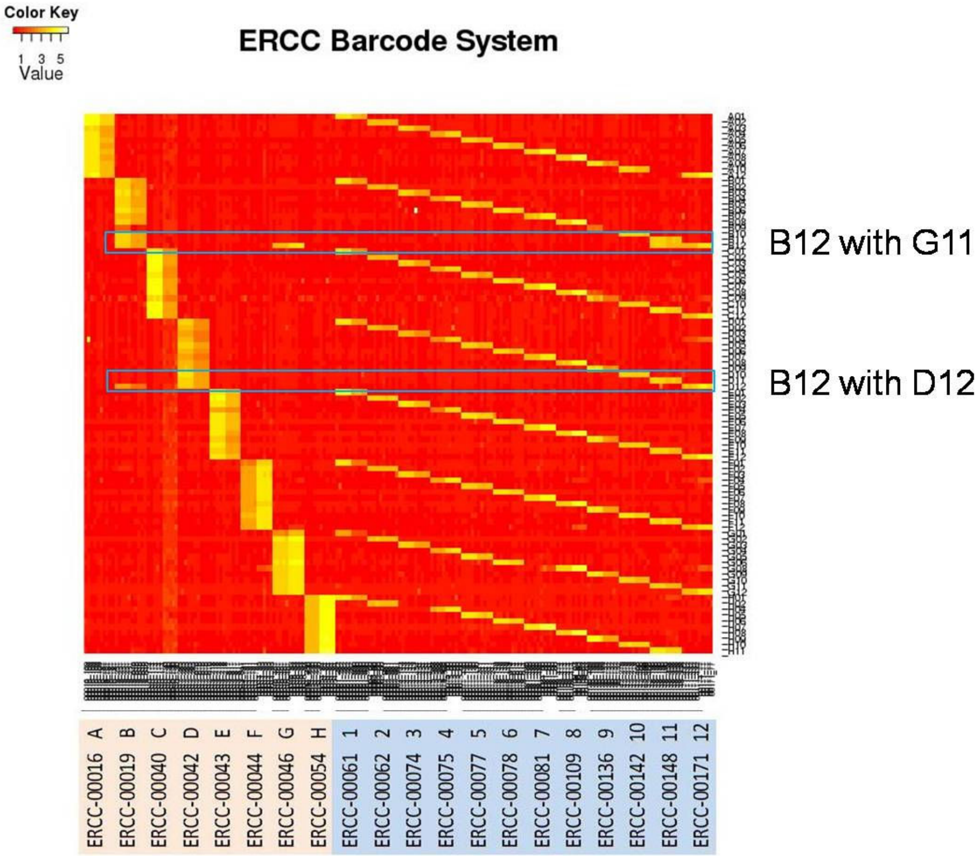

Figure 5. ERCC molecular barcode system for sample tracking throughout the hybridization process.

The 20 ERCC transcripts are listed along the horizontal axis. Well positions on a 96-well plate are listed on the right of the heat map. Highly expressed probes in each sample identify the sample's position in the 96-well plate specified by row (A-H) and column (1-12). Sample swaps or cross-contamination are detected by the presence of signal in an unexpected column or row.

Normalization

A description of normalization processes used for the microarray dataset is provided in more detail in a separate white paper, *Microarray Data Normalization*.

Online Presentation of Microarray Gene Expression Data

Data presented online are normalized data across the entire set of microarray samples and can be viewed in heat map format in z-score or log2 expression value formats using user-specified color maps. Probes were initially mapped to genes using RefSeq version 52, but as of October 2012, are now mapped using RefSeq version 53. Periodic updates to new RefSeq versions may occur in the future. Microarray gene expression data can be downloaded as .csv files containing normalized expresson values and probe and sample metadata or accessed via the API in both raw and normalized formats. The API also allows access to historical normalized data using the original normalization process as well as probe mapping to RefSeq version 52. The ontology structure tree is also available in XML format.

Three types of expression search tools are available on-line: differential, correlative and gene classification. Differential search finds probes that show the greatest difference between two sets (target and contrast) of user-defined structures. For each probe, a 2-sample t-test is performed followed by Benjamini and Hochberg false discovery rate correction. The null hypothesis is that the average expression level of samples in the contrast set of structures is less than the average expression level of samples in the target set of structures. Resulting p-values are sorted in ascending order. Search results can also be sorted by fold-change (log ratio of expression) in descending order. Correlative search (NeuroBLAST) finds probes with similar expression profile to the selected seed probe over all samples within a user-specified structure. Pearson's correlation coefficient is computed for all probes and results ranked in descending order. Gene classification search is available on the microarray landing page, where a number of pre-set categories are available to click and browse. On any heatmap display, Gene Classification search is available by clicking the radio button and selecting from a drop down menu.

Digital Imaging and Processing of Histologically Stained Sections

Digital imaging of 6x8 large format slides was performed the Kodak/Creo flatbed iQsmart3 scanner at 215,000 dpi resolution (4.65 µm/pixel). Endpoint adjustments were done prior to scanning to compensate for variance in light intensity across each slide. Digital imaging of 2x3 slides was done using the ScanScope XT (Aperio Technologies Inc., Vista, CA) with slide autoloader to enable scanning of multiple slides at a time. Gain adjustments were made to lighten background for the darker silver stain. Final resolution of 2x3 images was 1 µm/pixel. All images were databased and preprocessed, then QC'd to ensure optimal focus and that no process artifacts were present on the slide images. Images that passed this initial QC were further assessed to ensure that the staining data were as expected. Once all QC criteria were met, images became available for annotation of anatomic structures as well as for anatomic visualization and mapping processes described below.

MRI Image Postprocessing

All T1-weighted volumes were anatomically segmented using FreeSurfer's *recon-all* pipeline (http://surfer.nmr.mgh.harvard.edu/, Dale et al, 1999; Fischl et al, 1999, 2000; Segonne et al, 2004). Operations include intensity inhomogeneity correction, tissue classification (grey/white matter and cerebrospinal fluid), skull stripping, cortical surface reconstruction, and cortical and subcortical segmentation of structures based on tissue class likelihood estimates, geometric features, and structure-wise likelihood estimates. The pipeline required an initial mapping of each T1-weighted scan to MNI space. FreeSurfer's affine registration was used for H0351.2001 and H0351.2002. A deformable method was needed for the rest of the brains, which were imaged *ex cranio*.. First, a rigid alignment was performed with FMRIB's Linear Image Registration Tool (FLIRT, http://fsl.fmrib.ox.ac.uk/fsl/fslwiki/FLIRT). This step was followed by a nonrigid registration using the Advanced Normalization Tools (ANTS, http://picsl.upenn.edu/ANTS/). To allow mapping to the Nissl images described in the next section, the deformation field needed to be constrained so

that gyri could still be matched between MRI and Nissl. To achieve this aim, a large Gaussian regularizer was imposed to smooth the deformation field.

Anatomic Mapping and Visualization

To enable unified navigation and visualization, spatial correspondences between 2x3 images, 6x8 images and MRI volumes for each brain were obtained via a series of assisted registration processes.

Registration started with the construction of virtual slab images by creating mosaics from representative Nissl images from 2x3 blocks. Typically, the last image from a block Nissl series, where macrodissection sites were annotated, was chosen as the representative image. For each slab, an SVG (scalable vector graphics) file was created containing pointers to downsampled representative block images. Each block was visually placed (rotation and translation) into its correct orientation using Inkscape (http://www.inkscape.org; an open source SVG editor) creating a virtual slab image. The transform parameters for each block was parsed from the SVG file and saved for downstream use.

In the next step, each virtual slab image was placed in context of the T1-weighted MRI volume. The removal of the cerebellum and brain stem from the cerebrum during tissue dissection caused significant deformation compared to the brain in its native conformation in the skull. Further deformation was introduced during the slabbing procedure where the type and severity of the distortion varied from slab to slab. The severity of the overall deformation necessitated an intensive landmark based registration process in order to establish spatial correspondences. For each slab, 50 to 100 corresponding landmark points were manually placed on the virtual slab image and T1 MRI volume using the facility provided by BioImage Suite (http://www.bioimagesuite.org). The landmark information was saved as text files for further processing. Initially, a “best-fit” affine transform was estimated that coarsely oriented a slab within the 3-D MRI volume. A deformation field was then estimated from the remaining displacement using thinplate radial base function interpolation.

As needed other block images were registered to the “representative” block image. Similarly other slab based images (blockface, 6x8 images) were registered to the “virtual” slab image. These additional transforms combined with those described above allowed spatial correspondences to be established between 2x3 images, 6x8 images and MRI volumes.

Results from the registration processes above allowed the construction of MRI-based navigation maps shown in 2-D multiplanar view. For each position in 3-D MRI space, a nearest slab and nearest block were pre-computed allowing the use of the 3-D MRI volumes to navigate through different slabs and blocks. Inversely, information annotated in block images was projected back into MRI space. For example, the “center” location of macro-dissection and LMD sites were mapped back into MRI space and used as representative locations for visualization of the spatial variation in gene expression for each probe.

These processes were done for each brain separately such that spatial correspondences of histology data and mapping of gene expression sampling sites are done and presented online in native MRI space for each brain.

REFERENCES

Atz M et al (2007) Methodological considerations for gene expression profiling of human brain. *J Neurosci Methods* 163:295-309.

Culling CFA, Allison RT, Barr WT (1985) Haemotoxylin and its counterstains. In: *Cellular Pathology Technique, 4th Edition*, pp 160-161. Butterworths, London.

Dale AM, Fischl B, Sereno MI (1999) Cortical surface-based analysis. I. Segmentation and surface reconstruction. *Neuroimage* 9:179-194.

Durrenberger PF et al (2010) Effects of antemortem and postmortem variables on human brain mRNA quality: a BrainNet Europe study. *J Neuropathol Exp Neurol* 69:70-81.

External RNA Controls Consortium (2005) Proposed methods for testing and selecting the ERCC external RNA controls. *BMC Genomics* 6:150.

Fischl BR, Sereno MI, Dale AM (1999) Cortical Surface-Based Analysis II: Inflation, Flattening, and Surface-Based Coordinate System. *NeuroImage*, 9:195-207.

Fischl B, Salat D, Busa E, Albert M, Dieterich M, Haselgrove C, van der Kouwe A, Killiany R, Kennedy D, Klaveness S, Montillo A, Makris N, Rosen B, Dale A (2002) Whole brain segmentation: Automated labeling of neuroanatomical structures in the human brain. *Neuron* 33:341-55.

Garvey W, Fathi A, Bigelow F, Jiminez C, Carpenter B (1987) A reliable silver impregnation technique for the nervous system. *J. Histotechnology* 10:245-247.

Johnson WE, Li C, Rabinovic A (2007) Adjusting batch effects in microarray expression data using empirical Bayes methods. *Biostatistics* 8:118-127.

Lein ES et al (2007) Genome-wide atlas of gene expression in the adult mouse brain. *Nature* 445:168-176.

Li B, Ruotti V, Stewart RM, Thomson JA, Dewey CN: RNA-Seq gene expression estimation with read mapping uncertainty. *Bioinformatics* 2010, 26**:**493-500.

MAQC Consortium et al (2006) The MicroArray Quality Control (MAQC) project shows inter- and intraplatform reproducibility of gene expression measurements. *Nature Biotech* 24:1151-1161

Meyer LR, Zweig AS, Hinrichs AS, Karolchik D, Kuhn RM, Wong M, Sloan CA, Rosenbloom KR, Roe G, Rhead B, et al: The UCSC Genome Browser database: extensions and updates 2013. *Nucleic Acids Res* 2013, 41:D64-69.

Schroeder A, Mueller O, Stocker S, Salowsky R, Leiber M, Gassman M, Lightfoot S, Menzel W, Granzow M, Ragg T (2006) The RIN: an RNA integrity number for assigning integrity values to RNA measurements. *BMC Mol Biol* 7:3.

Segonne, F, Dale, AM, Busa, E, Glessner, M, Salat, D, Hahn, HK, Fischl, B (2004) A hybrid approach to the skull stripping problem in MRI. *Neuroimage* 22:1060-1075.

APPENDIX A:
QUALITY CONTROL AND SCIENTIFIC QUALITY ASSESSMENT

In order to ensure quality data, numerous quality control (QC) checkpoints were incorporated throughout the entire microarray data production pipeline for the Allen Human Brain Atlas, as illustrated diagrammatically in Figure A1. As standard practice at the Allen Institute, such QC checkpoints were used to qualify material to advance further down the pipeline and to measure, trend and improve all phases of laboratory operations.

The major QC processes and steps indicated in Figure A1 are summarized in Table A1. These range from tissue qualification and assessment through generation and final qualification of the data for inclusion in the online public resource.

Table A1. QC steps

QC Step	Description
Q1	**Gross evaluation of the frozen, slabbed brain upon receipt after shipping.** The tissue was evaluated for issues that include breakage, surface irregularities, and freezing and storage artifacts. Each slab is assigned a pass/fail score based on these variables.
Q2	**Evaluation of full-coronal tissue sections.** Each section was checked for adhesion to the glass slide, tissue integrity, wrinkles, and drying artifacts.
Q3	**Histology assessment.** Each stained section was assigned a quality score (1-failing to 5-excellent) based on stain quality, tissue quality and observed artifacts.
Q4	**Evaluation of tissue blocks created by subdivision of frozen slabs.** Each resulting tissue block was evaluated for tissue quality and anatomic accuracy of subdivision.
Q5 (& Q15)	**Evaluation of tissue sections on 2”x3” slides.** Each section was checked for adhesion to the glass slide, tissue integrity, wrinkles, and drying artifacts.
Q6 (& Q13)	**Histology assessment.** Each stained section was assigned a quality score (1-failing to 5-excellent) based on stain quality, tissue quality and observed artifacts.
Q7 (& Q14)	**Post-dissection validation of tissue sampling.** The accuracy and quality of each dissected sample was compared to annotated images of the region to ensure the correct anatomic areas were captured.
Q8 (& Q12)	**Post-scanning assessment of 2”x3” histology images.** Each digital image was checked for orientation, focus, white balance, staining quality and tissue quality. Failed images were excluded from further processing.
Q9 (& Q11)	**RNA assessment.** Quantity and quality of total RNA isolated from dissected tissue samples was evaluated.
Q10	**Microarray expression assessment.** All microarray data were analyzed to identify outliers and to verify that the correct samples were processed and associated with the correct brain region. For samples identified as outliers or incorrectly tracked, microarrays were re-run.
Q11	See Q9.
Q12	See Q8.
Q13	See Q6.
Q14	See Q7.
Q15	See Q5.
Q16	**Assessment of block-face images.** Each block-face image was evaluated for focus, image quality and orientation at the time of image acquisition, thereby allowing real-time adjustments to ensure that each image passed QC.
Q17	**MRI assessment.** Each set of imaging data was evaluated for contrast, consistency of signal across the dataset and signal-to-noise ratio.
Q18	**cRNA QC.** Each cRNA product is evaluated for yield and product size.

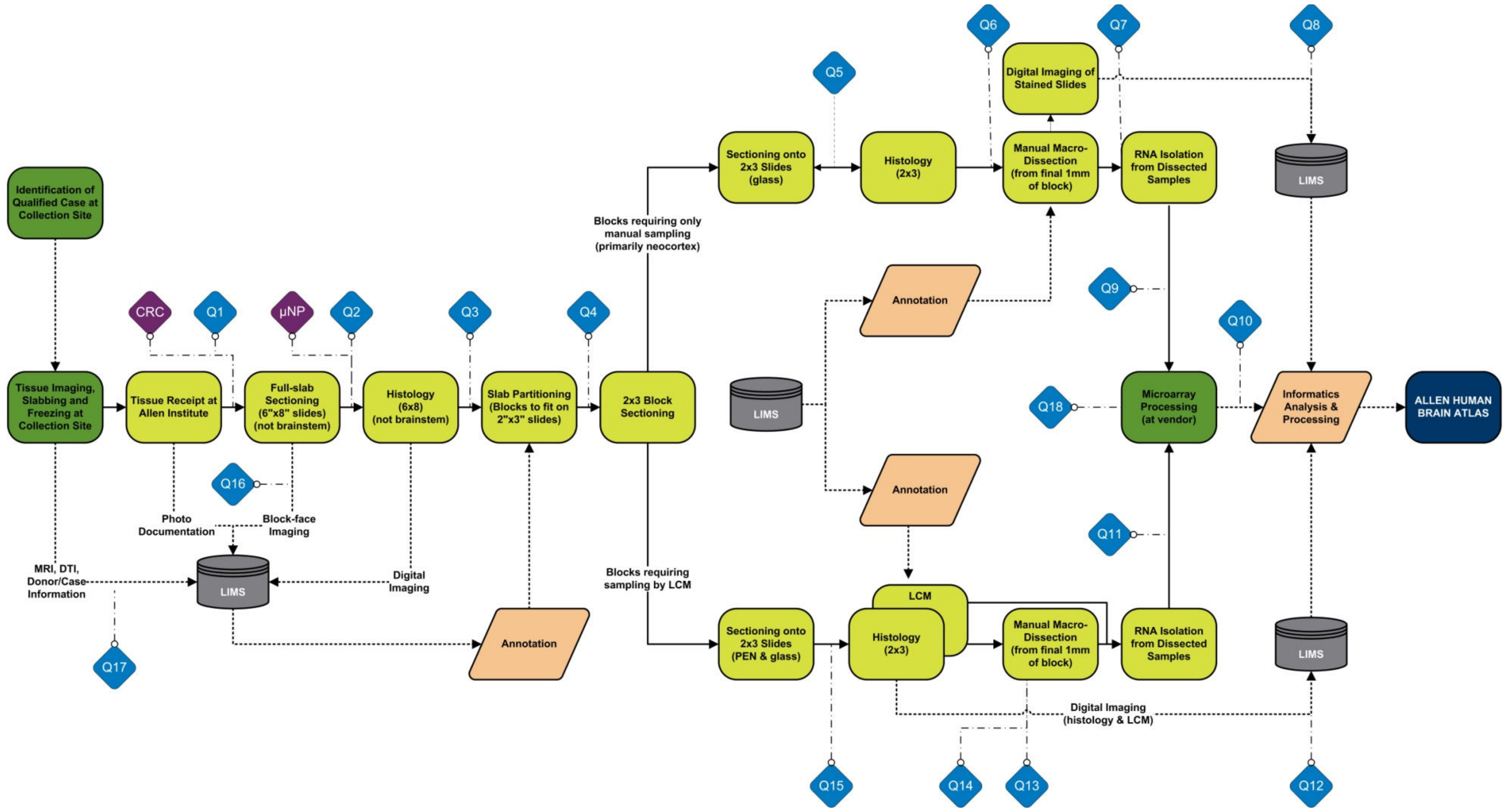

Figure A1. Data production pipeline indicating key quality control (QC) steps for the whole brain microarray survey of the Allen Human Brain Atlas.
CRC is the Case Review Committee, a group of experts that evaluate the available donor and tissue information to qualify cases for data generation. µNP is the microneuropathology screen. LIMS is the Laboratory Information Management System in which all data are tracked and processed for presentation of data online.
