# Supplementary material for: Human brain gene expression profiles of the cathepsin V and cathepsin L cysteine proteases, with the PC1/3 and PC2 serine proteases, involved in neuropeptide production
Source: Heliyon. 2018 Jul 3;4(7):e00673. doi: 10.1016/j.heliyon.2018.e00673 (PMC6037879; doi:10.1016/j.heliyon.2018.e00673)
Supplement: Technical White Paper _spl_3 — Technical white paper #3 “Microarray Data Normalization” March 2013 v.1. Available for download at https://help.brain-map.org/display/humanbrain/Documentation. [file mmc4.pdf]

ALLEN INSTITUTE *for* BRAIN SCIENCE
Fueling Discovery

ALLEN **Human Brain Atlas**

TECHNICAL WHITE PAPER: MICROARRAY DATA NORMALIZATION

The ALLEN **Human Brain Atlas** is a publicly available online resource of gene expression information in the adult human brain. Comprising multiple datasets from various projects characterizing gene expression in human tissue, a major component is the 'all genes, all structures' microarray-based gene expression survey in human brain with accompanying anatomic and histologic data.

The Allen Human Brain Atlas microarray dataset of 6 brains was generated and publicly released over approximately three years. This white paper describes two sets of normalization methods and processes for this dataset: an original normalization strategy used for the first 4 brains and an updated normalization strategy implemented with the complete dataset of 6 brains.

Normalized data values are accessible in multiple ways in the Allen Human Brain Atlas: (1) through displayed data values and 'download this data' links in the interactive web-based application; (2) the application programming interface (API); and (3) downloadable .csv files, including archived files for historical array data processed through the original normalization strategy.

MICROARRAY SURVEY OVERVIEW

The experimental design of the microarray survey is summarized below to provide context for the normalization methods described in this white paper. Detailed operational, technical and quality control (QC) methods and processes for tissue procurement, processing, anatomic sampling, RNA isolation and microarray hybridizations are available in a separate technical white paper (see *Whole Brain Microarray Survey*).

The goal of the microarray survey was to systematically profile gene expression throughout all major regions of neurotypical ("control") adult human brains. Approximately 500 anatomically discrete samples per hemisphere were collected from cortex, subcortex, cerebellum and brainstem of each brain and profiled for genome-wide gene expression using a custom Agilent 8x60K cDNA array chip. Two methods were used to dissect samples: (1) a scalpel-based manual macrodissection method primarily for cortical and other relatively large uniform samples; and (2) laser microdissection (LMD) for small or oddly-shaped structures such as subcortical or brainstem areas. To ensure the highest data quality possible, multiple quality control (QC) gateways (e.g. for RNA quality, cDNA-labeling quality, hybridization and array chip quality) were implemented throughout the experimental workflow. In addition, a number of technical and biological replicates were included to assess reproducibility of data both within a batch of samples and among multiple batches of samples. Over the multi-year course of the project, brains were processed serially (i.e. expression profiles for the first brain were completed before profiling the second brain), and multiple batches of samples were submitted per brain. Due to the nature and timing of dissections, each batch often primarily or entirely comprised a single category of anatomic samples. For example, early batches within each brain comprised macrodissected cortical structures whereas later batches comprised LMD brainstem structures, thus creating different anatomic sample compositions across batches. To assess batch-to-batch variance within a brain and across all brains, two types of control samples were submitted with each batch, an internal control (IC)

and a Human Brain Atlas control (HC), at n = 2 per control per batch. Table 1 below summarizes the composition and purpose for each of these controls.

Table 1. Control samples for each array batch. Each array batch contained experimental samples from a single brain in addition to the control samples described.

	**Internal Control (IC)**	**HBA Control (HC)**
**Composition**	Pooled RNA comprising 150-300 cortical macrodissected samples from each brain.	Pooled RNA from ~300 cortical macro samples from the first brain. (Doubles as IC for brain 1).
**Number per batch**	At least 2	At least 2
**Run with….**	Each batch for the brain from which the IC was derived.	Each array batch across all brains.
**Purpose**	Indicator of batch to batch variance within a brain.	Indicator of batch to batch variance within a brain and among all brains.

NORMALIZATION PROCESSES

In a large scale microarray project such as the Allen Human Brain Atlas, in which thousands of samples were processed during an approximately 3-year time span, systematic biases were likely introduced into the dataset for various reasons such as drifts due to age of reagents or array chips, reagent lot changes, variation in RNA integrity among samples, variation in RNA quality due to different sample capturing methods, and other stochastic variations. In addition, for the first n = 3 brains, array data were generated by Beckman Coulter Genomics whereas the second n = 3 brains were processed by Covance Genomics Laboratory. High comparability of results between service providers was a key metric in selecting Covance Genomics Laboratory to minimize effects of a vendor change. The purpose of normalization is to minimize the effects of these non-biological biases while keeping biological variance intact so that within and across brain comparisons primarily reveal differences and similarities that are biologically relevant.

Two sets of normalization processes have been applied to the Allen Human Brain Atlas microarray dataset (see Table 2). The original normalization strategy was employed for most of the project (through n = 4 brains) and the updated normalization strategy was implemented upon completion of the n = 6 dataset. The updated strategy addressed previously unknown systematic biases and utilized processes that are a better fit for the array dataset.

Table 2. Summary of original and current normalization processes in the Allen Human Brain Atlas. In both processes, normalization methods were first applied within-brain to adjust data within a sample batch and among sample batches, and subsequently applied across multiple brains (Cross Brain) to enable comparisons across two or more brains.

	**Original Normalization Processes**	**Current Normalization Processes**
**Within Brain**	1. 75th percentile alignment of intensity distributions of all samples within a batch.<br>2. Adjustment for batch effects using ComBat.	1. Preprocessing for array-specific biases.<br>2. 75th percentile alignment of intensity distributions of all samples within a batch.<br>3. Adjustment for RNA quality differences among samples within a batch.<br>4. Adjustment for batch effects by alignment of IC and HC across batches.<br>5. Adjustment for dissection method (macrodissection vs. LMD) across batches.
**Cross Brain**	3. 75th percentile alignment of all samples across all available brains, using the first processed brain (H0351.2001) as the reference.	6. Alignment of HC values across all brains.<br>7. Alignment of brain-wise mean expression levels.

Original Normalization Methods

Normalization Within a Single Brain

Gene expression data for samples passing quality control were normalized within a brain by first aligning data within a batch, then by addressing batch effects for all batches within that brain.

Within-batch normalization was performed using a 75% centering algorithm. Expression distributions of all samples in a single batch were normalized to have the same 75th percentile expression values. The effect of this normalization is illustrated in Figure 1 in which the first boxplot (A) shows raw data of all samples in 5 batches of a single brain, the second boxplot (B) shows the effect of the 75% centering algorithm after application to all raw data across all batches, and the third boxplot shows (C) the result of within-batch normalization for 5 different batches where batch differences are clearly seen.

Systematic batch effects were addressed by application of a cross-batch normalization algorithm, ComBat (Johnson et al., 2007; http://statistics.byu.edu/johnson/ComBat/), across an entire dataset from a single brain. The ComBat method applies either a parametric or non-parametric empirical Bayes framework for adjusting data that is robust to outliers in a given data set. The location (mean) and scale (variance) model parameters were specifically estimated by pooling information across genes in each batch to shrink the batch effect parameter estimated toward the overall mean of the batch effect estimates. After the ComBat algorithm was applied, the differences between batches disappear, as shown in Figure 1D. Raw expression values, within-batch normalized expression values, and cross-batch normalized expression values were all uploaded into the database for further data analyses and data visualization.

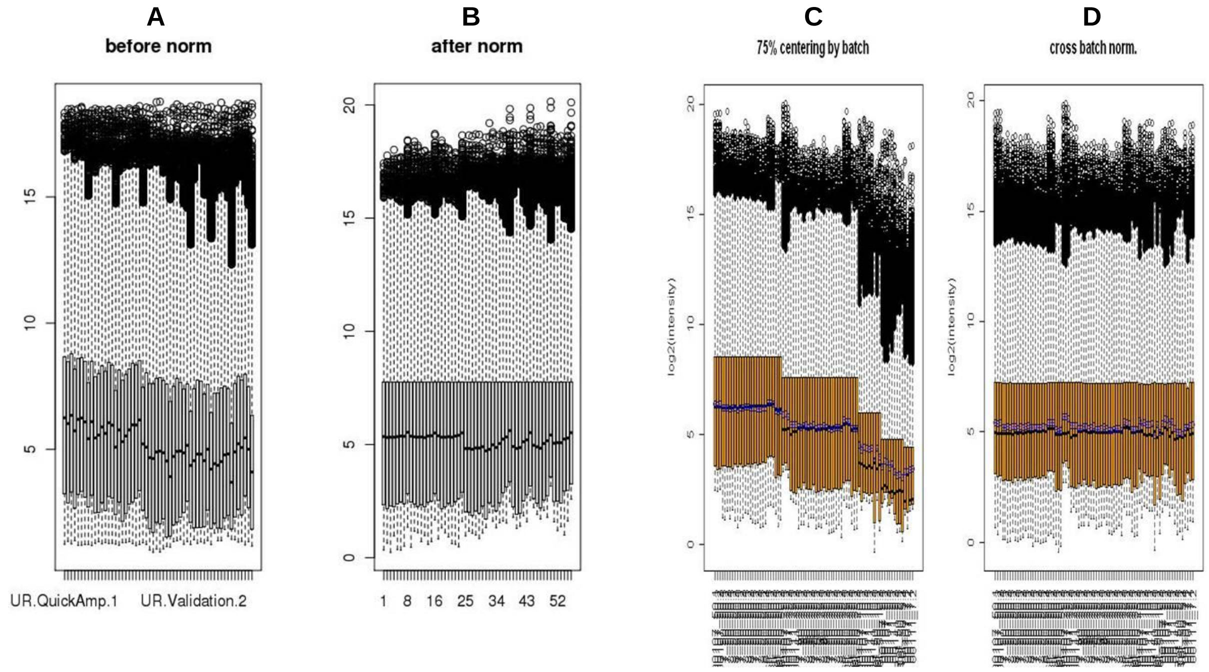

Figure 1. Effect of 75% centering normalization and cross-batch normalization over 5 data batches within a brain. The first boxplot (A) shows the distribution of raw expression values for each sample prior to any normalization. The second boxplot (B) shows the application of the 75% centering algorithm to all samples across all batches. The third boxplot (C) displays the result of applying the 75% centering algorithm to each batch separately, i.e., within-batch normalization. The fourth boxplot (D) shows cross-batch normalization with the ComBat algorithm applied to the within-batch normalized data from (C).

Normalization Across Multiple Brains
To allow comparison of microarray data across 2 or more brains, a final cross-brain normalization was performed by aligning the mean 75th percentile expression values of all internal reference control samples of each brain to that of the first brain.

Current Normalization Methods

As array data from additional brains were added to the dataset, further evaluation of the original normalization strategy and assessment of additional or alternative normalization methods to better address technical biases was performed and an updated overall normalization strategy was adopted and implemented.

Normalization Within a Single Brain
Systematic array-specific technical biases can arise due to factors such as variations in hybridization thermodynamics, RNA and cDNA variation, and regional hybridization inhomogeneity (Reimers, 2010). Probe GC content, location in the chip, and experiment-wise mean intensity for each probe were used as variables to characterize and correct array-specific biases. For each probe, individual sample deviations from a robust batch-wise average were modeled as a function of GC-content, location, and average probe intensity (see Figure 2, upper row). A flexible multivariate local regression (LOESS) surface was fitted to these deviations and a correction applied by subtracting the model-fitted values from the deviations. The effects of this correction are illustrated in the lower row of Figure 2 in a dataset containing groups of structure replicates (dissected from different areas of the same anatomic structure), in which the correlation of deviations from average between structure replicates is increased after correction.

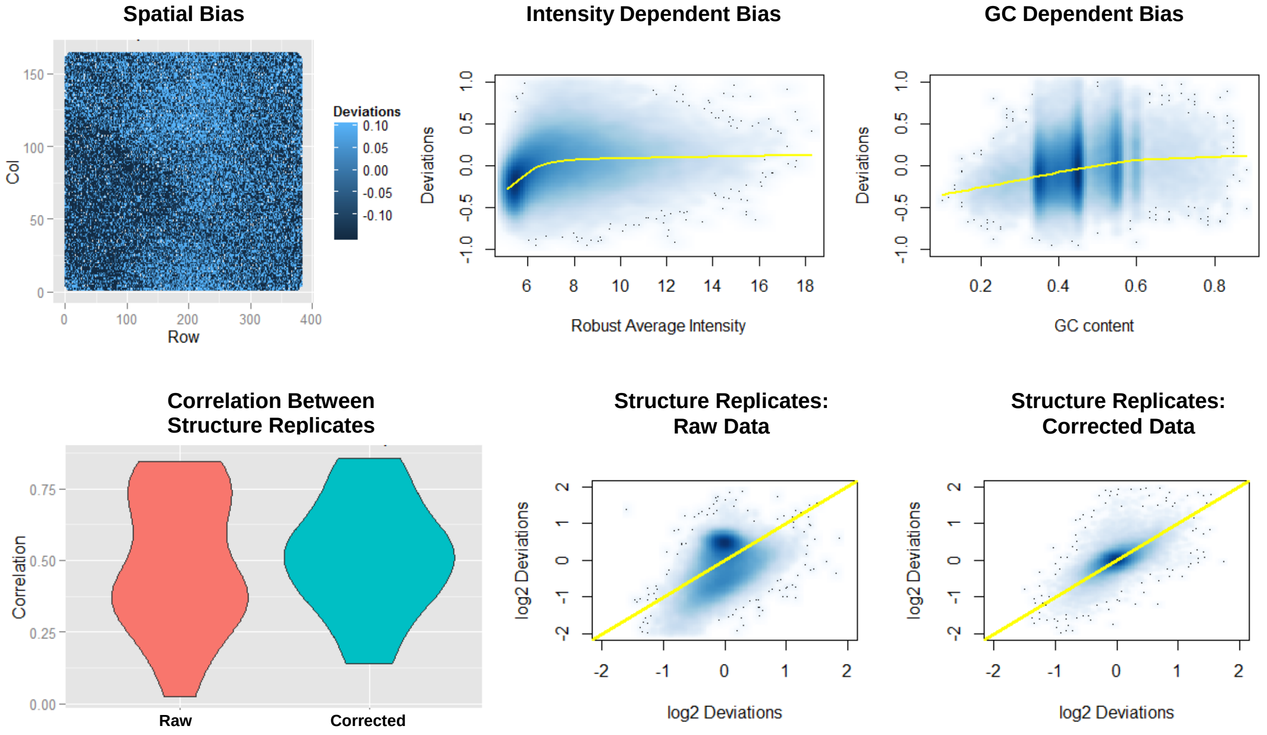

Figure 2. Probe related non-biological biases and their corrections. The upper row demonstrates non-biological variation introduced by probe location in the chip, mean intensity of probe, and GC content of probe. The upper left panel shows deviations of values over the surface of a single chip relative to an experiment-wise average at each location, the upper middle panel shows the deviations from average intensity and the upper right panel shows deviations as a function of GC content. The lower row shows the effects of the correction, with the lower left panel showing the distribution of correlation between structure replicates before and after correction, and the lower middle and lower right panels showing structure replicate correlation of deviations from average before and after correction, respectively. (Figure courtesy Paul T. Manser, Virginia Commonwealth University.)

After preprocessing for array-specific artifacts, a 75th percentile alignment of expresson intensity distributions was performed for all samples within a batch.

In the Allen Human Brain Atlas dataset, a large source of variance was the dissection method (macrodissection or LMD) used to collect the samples. As described above, macrodissection was used for samples from cortical regions and large subcortical nuclei whereas LMD was used for samples from small or oddly shaped structures within subcortex, cerebellar nuclei or brainstem structures. As examples, the supraoptic nucleus and dentate nucleus were dissected using LMD methods. To characterize the effects of dissection method on array data, a subset of data comprising structures with samples obtained by both macrodissection and LMD methods was selected for analysis to enable comparisons of dissection methods independent of differences in brain structure. The analysis first revealed that macrodissected samples have on higher average expression values and better RNA quality, as measured by RNA Integrity Number (RIN), compared to LMD samples (see Figure 3).

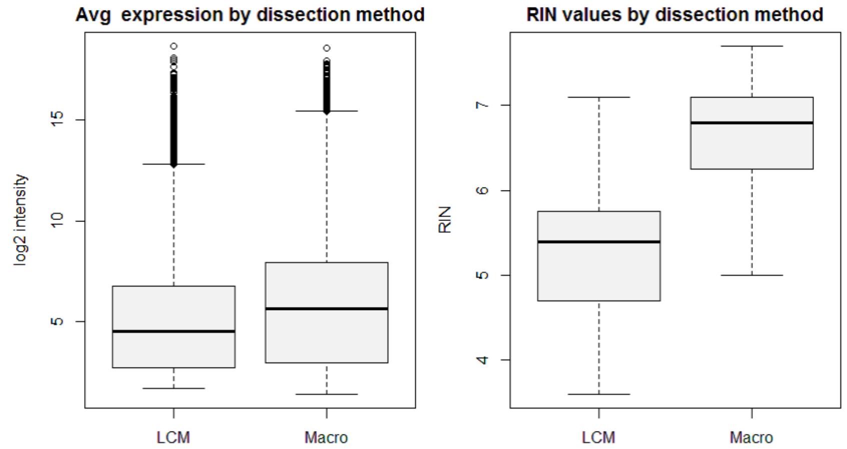

Figure 3. Macrodissected samples have higher average expression and RIN values than LMD samples collected from the same anatomic structure. Left panel: boxplots of expression intensity (log_2 scale) for LMD and macrodissected samples. Right panel: boxplots of RNA quality (RIN) for LMD and macrodissected samples. Whiskers in both plots represent 1.5*IQR from the upper or lower quartile. (Figure courtesy Paul T. Manser, Virginia Commonwealth University.)

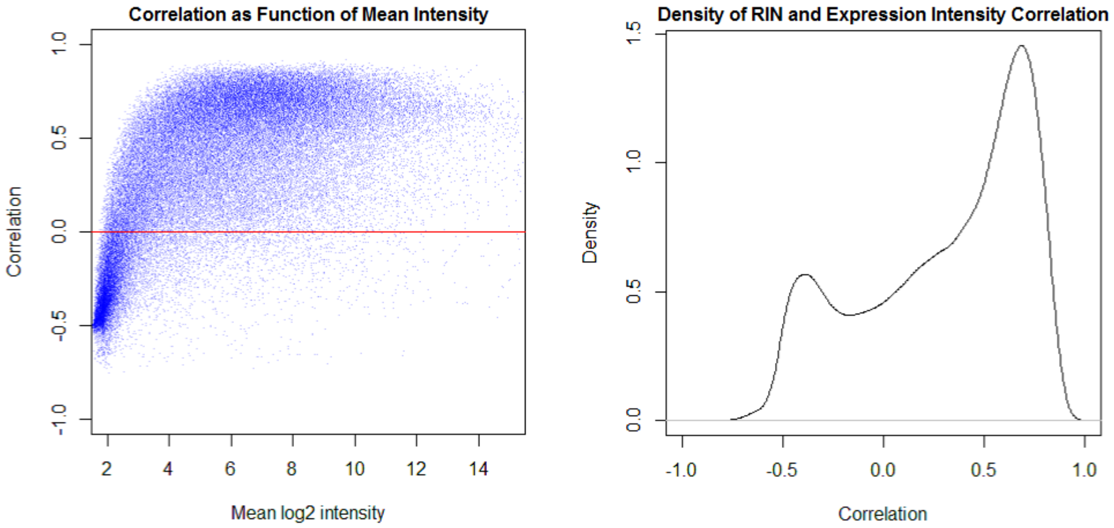

Figure 4. Relationship between RIN and expression. Left panel: relationship of a probe's mean expression intensity (log_2 scale) and the correlation between RIN and expression intensity for that probe. Roughly 25% of probes are negatively correlated with RIN. Low intensity probes tend to be negatively correlated, while higher intensity probes are positively correlated. Right panel: the distribution of probes (measured as density) as a function of correlation between RIN and expression intensity. (Figure courtesy Paul T. Manser, Virginia Commonwealth University.)

Further characterization of the relationship between expression intensity and RIN values revealed that low intensity probes tended to be negatively correlated, while higher intensity probes were generally positively correlated with RIN (Figure 4). However, as shown in Figure 4 (left panel), intensity is not a perfect predictor of RIN correlation since there are probes with a specific intensity level that are negatively correlated with RIN whereas different probes with the same intensity are positively correlated. Based on these findings, normalization was done for each array batch using local regression to construct a model for each probe by fitting expresson deviations from the average expression to a function of RIN values. These probe-wise models were applied to estimate the bias for each probe and the correction made by subtracting the estimated bias.

Corrections for array-specific artifacts, 75th percentile intensity distribution alignment of all samples within a batch, and RIN-related biases were all performed within each batch of array samples. The combined effect of these normalization steps is illustrated in Figure 5, which shows improved alignment of intensity distributions within each batch (represented by a unique color block).

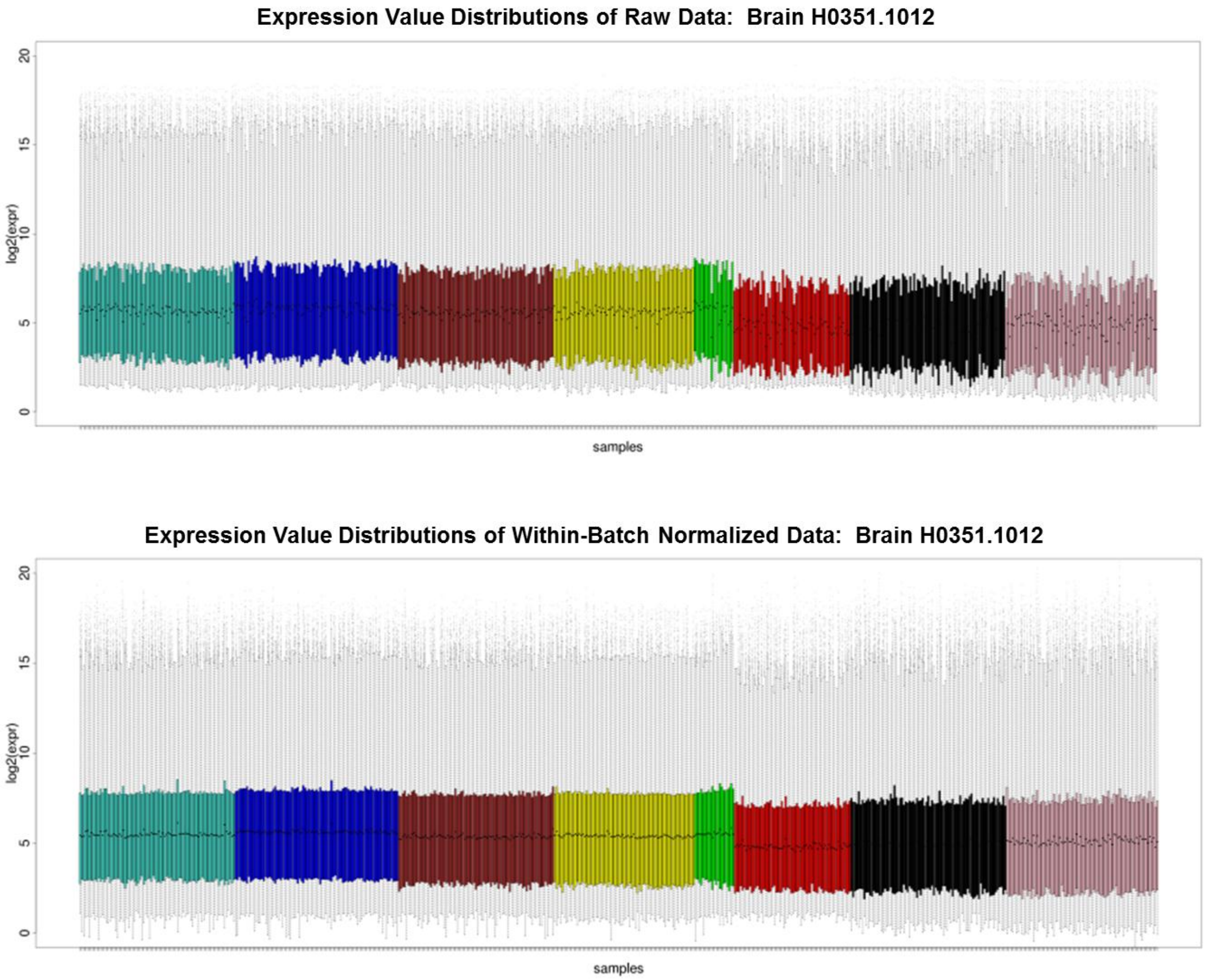

Figure 5. Effects of within-batch normalization in 8 batches of brain H0351.1012. Distribution of raw expression values for each sample in each batch prior to normalization (upper panel) and after within-batch normalization step to correct array-specific artifacts, align intensity distriubtions at 75th percentile and address RIN-associated biases (lower panel). Each batch is represented by a unique color (teal, blue, rust, yellow, green, red, black, or pink) and each vertical boxplot within each color represents the intensity distribution of an individual sample.

Following within-batch corrections, a second set of normalization steps focused on normalization across all batches from a single brain (cross-batch normalization). The original normalization process applied the widely used ComBat method for correcting batch biases. ComBat assumes a model for the mean and variance of data within batches and adjusts each batch to meet the assumed model specification by standardizing the mean and variance over batches. While this is a good approach when the composition of samples from batch to batch is equivalent, it is less appropriate when sample composition varies greatly among batches because the assumption that all batches should have similar means and variances is not necessarily valid. In the case of the atlas Allen Human Brain Atlas array data, the operational workflow resulted in batches that were different from each other with respect to brain structures represented. For example, the first sample batches for a brain typically contained macrodissected samples primarily or exclustively from cerebral cortex, the middle sample batches typically contained subcortical and cerebellar samples and the last sample batches contained primarily or exclusively samples from brainstem. Thus, it is reasonable to expect that the means and variances of different sample batches are dissimilar due to biological (anatomic) variation across the batches. Analyses of the data confirmed the expectation of dissimilar means and variances across batches. In addition, analyses of the effects of ComBat on the array dataset showed that differential expression between structures and expected variation in expression profiles of selected marker genes were diminished or suppressed (data not shown), suggesting that ComBat normalization may have obscured some of the biological variation present in the dataset.

As described above and summarized in Table 1, the two sets of control samples included in each batch were the IC (internal control) samples common to all batches within each brain and the HC (HBA control) samples common to all batches throughout all brains. Because each IC sample is from the same pool of RNA, any variation in IC array data is due to technical and environmental variation rather than biological variation. The same is true for the HC controls. Therefore to correct for batch biases, both IC and HC samples were utilized as references to align data across all batches within a brain. For each batch, an offset was calculated by determining the difference between the 10% trimmed mean of HC and IC samples over all batches and the 10% trimmed mean of HC and IC samples for each batch (see Figure 6). The average of the two offsets for each batch were applied to all samples within that batch to complete the alignment. The offset values from HC and IC controls were highly correlated (R^2=0.941).

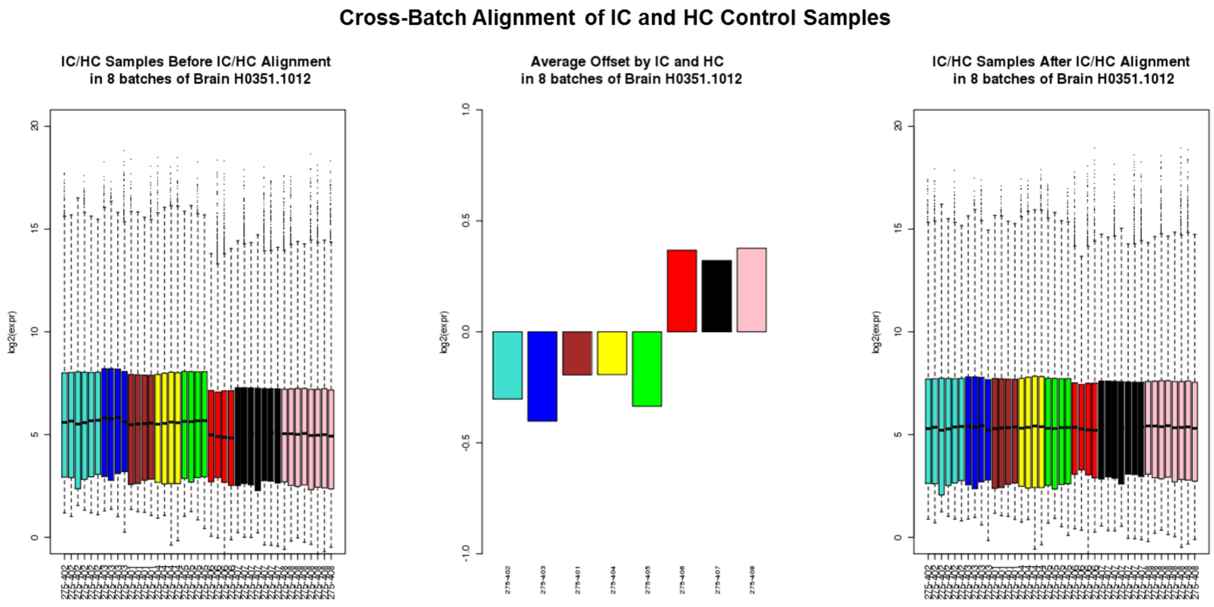

Figure 6. **Control sample data before (left) and after alignment (right) using offset values (middle) for brain H0351.1012.** Each boxplot represents log_2 expression intensity distributions for a single IC or HC sample (left and right panels). At least 2 IC and 2 HC samples were included with each batch. The offset for each batch was determined by subtracting the mean expression intensity of all HC and IC control samples within a batch from the mean expression intensity of control samples across all batches (note the reduced scale for the offset panel). Each batch is represented by a unique color that is consistent across all panels.

After alignment using control samples, significant batch differences were still present due to the effects of dissection method (see Figure 7, middle panel). In most cases, each batch often contained primarily or exclusively samples dissected with one or the other method. A modified quantile normalization was used to reduce the bias introduced by sample dissection methods while still maintaining anatomic structure-based biological variation. For each brain, the average of all macrodissected samples (Avg.Macro), average of all LMD samples (Avg.LMD) and the average of all samples (Avg.all) were calculated. Quantile-quantile (q-q) mapping between Avg.Macro and Avg.all and q-q mapping between Avg.LMD and Avg.all were set. For each macrodissected sample, each probe's value was normalized by first finding the nearest expression value of Avg.Macro and subsequently determining the mapped value in Avg.all. For LMD samples, each probe was mapped to Avg.LMD then to Avg.All. This process was repeated within each individual brain to complete cross-batch within-brain normalization (Figure 7, right panel).

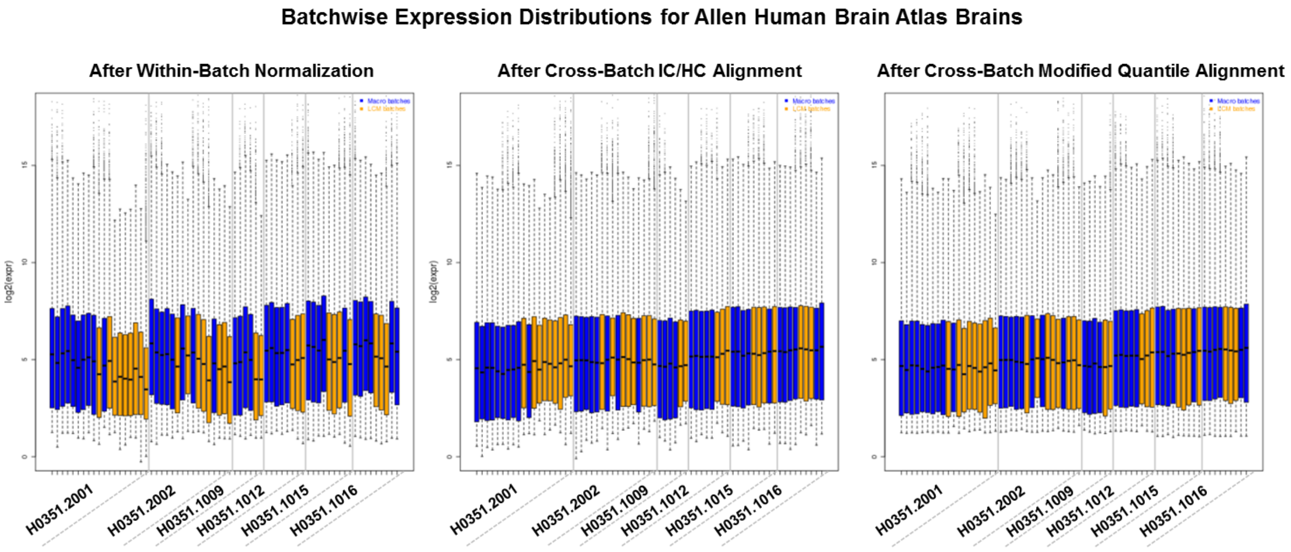

Figure 7. Effects of cross-batch normalization processes on batchwise expression distribution. Mean expression distributions for each batch in six brains are shown in each panel. Blue boxplots correspond to batches primarily or entirely composed of macrodissected samples, gold boxplots correspond to batches primarily or entirely composed of LMD samples, and brain ID numbers are listed on the x-axis. Batchwise expression distributions after completion of within-batch normalization steps are shown in the left panel, and show the nature of the dataset prior to application of cross-batch normalization processes. Batchwise expression distributions within each brain become better aligned after completion of IC and HC control alignment across batches (middle panel), and after completion of cross-batch modified quantile alignment. The number of batches per brain varies depending on brain size and whether both hemispheres (H0351.2001 and H0351.2002) or a single hemisphere (remaining brains) was processed for microarray.

Application of modified quantile normalization lowered expression intensity values of macrodissected samples by 0.6 on average and increased expression intensity values of LMD samples on average by 1.1. To assess whether these normalizations overcorrected values, either lowering macrodissection values too much or increasing LMD values too much and thus resulting in increased false absent and false present calls, respectively, structure samples collected by both LMD and macrodissections from several brains were compared for present and absent calls relative to threshold. Specifically, sample pairs within each brain were selected for analysis when the criteria that the anatomic structure was collected by both macrodissection and LMD dissection were met. Thus, the effects of dissection method could be assessed while controlling for structure. The analyzed dataset contained over 17 sample sets from n = 4 brains. The number of probes above or below threshold were compared in macrodissected vs. LMD samples based on pre-quantile normalized expression values and post-quantile normalized values by building contingency tables, each with a fixed present/absent call threshold. Receiver operating curves (ROC curves) were constructed for data before and after quantile normalization by varying thresholds and calculating the effect on sensitivity and specificity (Figure 8). The ROC curves demonstrated increased sensitivity (true present) with quantile normalization without increased loss of specificity. In addition, analysis of contingency tables at a $\log_2$

expression threshold = 5 (roughly equivalent to the present/absent threshold in the standard quality control report for Agilent arrays), revealed that approximately 40% of probes in LMD samples that were below threshold prior to normalization shifted to above threshold after normalization. These probes were present pre- and post-normalization in the paired macrodissected samples, which were considered to be the better indicator of 'true positive' compared to LMD dissected samples. Thus, the shift in number of probes above threshold in LMD samples following modified quantile normalization step is thought to be a recovery of signal in those samples.

ROC Curves:
Before and After Modified Quantile Normalization

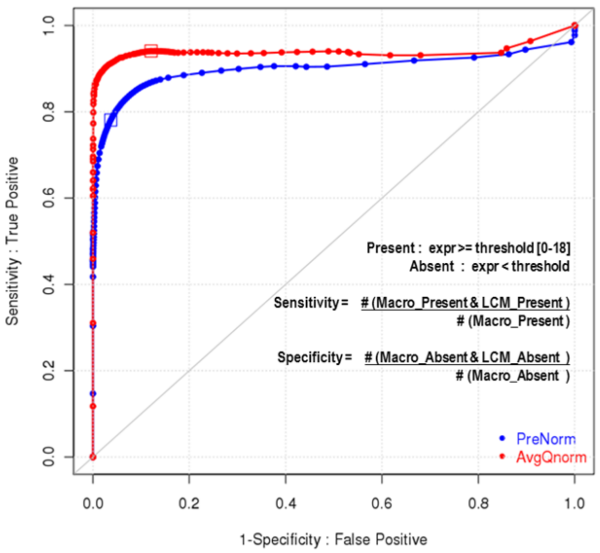

Contingency Table

Number of Probes Above and Below Threshold

Threshold = 5 (log_2 scale)		***Prenormalization***			
		M(-) /L(-)	***M(-)/L(+)***	***M(+)/L(-)***	***M(+)/L(+)***
Postnormalization	***M(-)/L(-)***	***24,340***	***0***	***1,656***	***0***
	M(-)/L(+)	***1,303***	***980***	***806***	***515***
	M(+)/L(-)	***0***	***0***	***1,849***	***0***
	M(+)/L(+)	***0***	***0***	***3,106***	***26,397***

M(-)/L(-): absent in macrodissected & absent in LCM
M(-)/L(+): absent in macrodissected & present in LCM
M(+)/L(-): present in macrodissected & absent in LCM
M(+)/L(+): present in macrodissected & present in LCM

Figure 8. **Modified quantile normalization improved sensitivity and specificity.** Sample pairs from multiple brains were selected for analysis when a specific anatomic structure was collected by both macrodissection and LMD so that the effects of dissection method could be assessed while controlling for structure. The left panel displays ROC curves for pre-normalized (blue) and post-normalized (red) expression values. The number of probes with expression values above threshold ('present') and below threshold ('absent') was calculated for each macrodissected sample and each LCM dissected sample. Sensitivity was the ratio of overlapping probes present in macrodissected and present in LMD samples relative to macrodissected samples. Specificity was the ratio of overlapping probes absent in macrodissected samples and absent in LMD samples relative to probes absent in macrodissected samples. ROC curves were drawn by plotting sensitivity and 1 – specificity at varying thresholds. The right panel gives an example contingency table of number of probes above and below threshold pre- and post-normalization at the present/absent log_2 threshold = 5. 26,397 probes were above threshold in both types of dissections both pre- and post-normalization. Approximately 40% of probes (n = 3,106 probes) absent in LMD samples but present in macrodissected samples (from the same structure) were recovered by normalization (highlighted in green).

Normalization Across Multiple Brains

To allow comparison of microarray data across 2 or more brains, a final cross-brain normalization was performed by first aligning HC control samples (Figure 9) in all batches across all brains, then by aligning brainwise mean expression levels (Figure 10). Alignment of HC control samples was accomplished by first determining the mean of all HC sample values across all brains to determine a reference value to which all brains were aligned. The mean of all HC samples within a brain was then determined and subtracted from the reference value to obtain the amount by which each sample in each brain was adjusted (the offset). The final normalization step was the alignment of mean expression intensity distributions across all brains using a global brain mean.

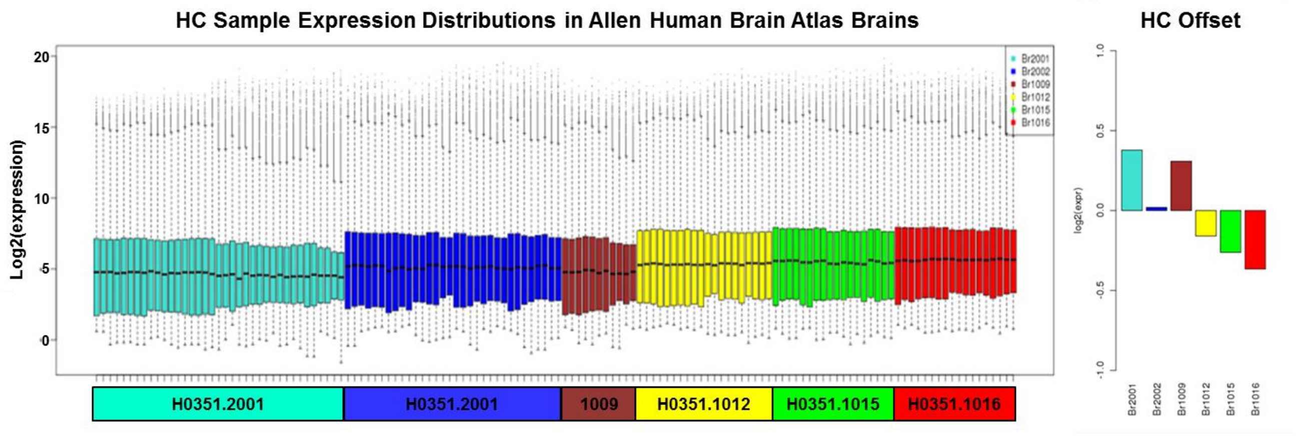

Figure 9. Distributions expression intensity values for each HC sample in the Allen Human Brain Atlas and the mean offset for each brain used to for cross-brain alignment of data. In the left panel, each boxplot represents an individual HC sample, with boxplot colors corresponding to the brain the sample was run with. At leaset two HC samples were included per batch for each brain. Offset corrections for each brain in the Allen Human Brain Atlas (right panel) were calculated by subtracting the mean expression intensity of all HC samples in a brain from the mean expression intensity of all HC samples from all brains.

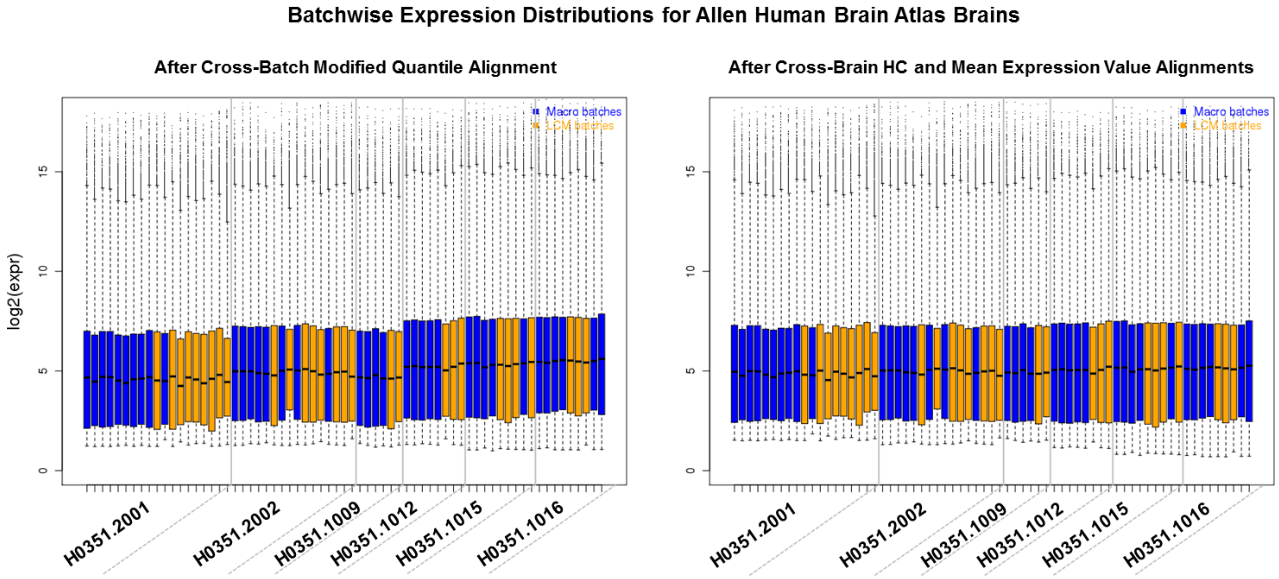

Figure 10. Effects of cross-brain normalization on batchwise expression distributions for all brains in the Allen Human Brain Atlas. The left panel shows batchwise expression distributions after completion of within-brain normalization steps but prior to cross-brain normalization, and is a reproduction of the right panel in Figure 7. The combined effects of cross-brain normalization steps which include alignment of values using HC control samples followed by alignment of global brain means, are shown in the right panel. Each boxlplot is the expression intensity distribution of a macrodissected (blue) or LMD (gold) batch. Brain IDs are indicated on the x-axis. Expression distributions are better aligned among the brains, while variability is still maintained across macrodissected and LMD batches (and thus, primarily different structure types) within a brain.

SUMMARY

The Allen Human Brain Atlas microarray-based ‘all genes, all structures’ gene expression survey of the adult human brain comprises n = 6 brains, with a combined total of approximately 4,000 unique anatomic samples characterized across 60,000 probes per sample. The project spanned approximately three years to process and collect array data on all samples, with data being made available to the public throughout the project.

Original normalization processes used for the atlas array data were relatively simple, involving three major steps to normalize data within brains and across brains. As the project progressed and additional array data collected, various patterns emerged that pointed to technical biases in the data that were not addressed in the original process. Normalization processes were updated to better and more appropriately account for these technical biases while maintaining biological variance.

The complete dataset of six brains with current (updated) normalized values is available online through an interactive application or as downloadable .csv files and via an API (http://human.brain-map.org). Historical datasets available in previous data releases using original normalization processes continue to be available as downloads and via the API for users who wish to access these data for analysis.

REFERENCES

Johnson, WE, Li, C, Rabinovic, A (2007) Adjusting batch effects in microarray expression data using empirical Bayes methods. *Biostatistics* 8:118-127.

Reimers, M (2010) Making Informed Choices about Microarray Data Analysis. *PLOS Comput Biol* 6: 1000786. doi:10.1371/journal.pcbi.1000786
